# Impact of Safety-Related Dose Reductions or Discontinuations on Sustained Virologic Response in HCV-Infected Patients: Results from the GUARD-C Cohort

**DOI:** 10.1371/journal.pone.0151703

**Published:** 2016-03-28

**Authors:** Graham R. Foster, Carmine Coppola, Moutaz Derbala, Peter Ferenci, Alessandra Orlandini, K. Rajender Reddy, Ludovico Tallarico, Mitchell L. Shiffman, Silke Ahlers, Georgios Bakalos, Tarek Hassanein, GUARD-C Study Group

**Affiliations:** 1 Institute of Cellular and Molecular Sciences, Queen Mary University of London, London, United Kingdom; 2 Unità Operativa Epatologia ed Ecografia Interventistica Ospedale Gragnano, Naples, Italy; 3 Hamad Medical Hospital, Doha, Qatar; 4 Medical University of Vienna, Vienna, Austria; 5 Unit of Infectious Diseases and Hepatology, Azienda Ospedaliero-Universitaria di Parma, Parma, Italy; 6 University of Pennsylvania, Philadelphia, Pennsylvania, United States of America; 7 Divisione di Medicina, Ospedale Elena D’Aosta, Naples, Italy; 8 Liver Institute of Virginia, Bon Secours Health System, Newport News/Richmond, Virginia, United States of America; 9 PROMETRIS GmbH, Mannheim, Germany; 10 F. Hoffmann-La Roche, Basel, Switzerland; 11 University of California San Diego, San Diego, California, United States of America; National Taiwan University Hospital, TAIWAN

## Abstract

**Background:**

Despite the introduction of direct-acting antiviral agents for chronic hepatitis C virus (HCV) infection, peginterferon alfa/ribavirin remains relevant in many resource-constrained settings. The non-randomized GUARD-C cohort investigated baseline predictors of safety-related dose reductions or discontinuations (sr-RD) and their impact on sustained virologic response (SVR) in patients receiving peginterferon alfa/ribavirin in routine practice.

**Methods:**

A total of 3181 HCV-mono-infected treatment-naive patients were assigned to 24 or 48 weeks of peginterferon alfa/ribavirin by their physician. Patients were categorized by time-to-first sr-RD (Week 4/12). Detailed analyses of the impact of sr-RD on SVR24 (HCV RNA <50 IU/mL) were conducted in 951 Caucasian, noncirrhotic genotype (G)1 patients assigned to peginterferon alfa-2a/ribavirin for 48 weeks. The probability of SVR24 was identified by a baseline scoring system (range: 0–9 points) on which scores of 5 to 9 and <5 represent high and low probability of SVR24, respectively.

**Results:**

SVR24 rates were 46.1% (754/1634), 77.1% (279/362), 68.0% (514/756), and 51.3% (203/396), respectively, in G1, 2, 3, and 4 patients. Overall, 16.9% and 21.8% patients experienced ≥1 sr-RD for peginterferon alfa and ribavirin, respectively. Among Caucasian noncirrhotic G1 patients: female sex, lower body mass index, pre-existing cardiovascular/pulmonary disease, and low hematological indices were prognostic factors of sr-RD; SVR24 was lower in patients with ≥1 vs. no sr-RD by Week 4 (37.9% vs. 54.4%; P = 0.0046) and Week 12 (41.7% vs. 55.3%; P = 0.0016); sr-RD by Week 4/12 significantly reduced SVR24 in patients with scores <5 but not ≥5.

**Conclusions:**

In conclusion, sr-RD to peginterferon alfa-2a/ribavirin significantly impacts on SVR24 rates in treatment-naive G1 noncirrhotic Caucasian patients. Baseline characteristics can help select patients with a high probability of SVR24 and a low probability of sr-RD with peginterferon alfa-2a/ribavirin.

## Introduction

Several direct-acting antivirals (DAAs) have been approved for the treatment of chronic hepatitis C and interferon-free regimens are now available [1–3]. These new agents increase sustained virologic response (SVR) rates, shorten the required duration of therapy for most patients, and are well tolerated. However, market access for these new therapies is limited in many countries [1,2,4,5]. Thus, despite major advances in the treatment paradigm for chronic hepatitis C, the combination of peginterferon alfa/ribavirin will continue to be used where access to these new DAAs is restricted.

Optimal use of peginterferon alfa/ribavirin involves careful patient selection prior to initiating treatment and close monitoring of the on-treatment virologic response. An important aspect of treatment with peginterferon alfa/ribavirin is the management of adverse events, since these lead to treatment discontinuation in more than 10% of patients [6]. Adverse events and laboratory abnormalities are managed initially by reducing the dose of peginterferon alfa and/or ribavirin and thus the result is reduced exposure to treatment. Reduced exposure to treatment in general, and to ribavirin in particular, is associated with lower SVR rates; thus, adverse events and dose reductions can have a significant impact on treatment efficacy [7–9].

Here, we report the results of a large, international, noninterventional cohort (GUARD-C) that was undertaken with the objective of identifying baseline predictors of safety-related dose reductions or discontinuations (sr-RD) to manage adverse events and laboratory abnormalities and the impact of sr-RD on SVR rates in patients receiving peginterferon alfa/ribavirin in routine clinical practice.

## Materials and Methods

### Study Design

GUARD-C is an international, prospective, non-randomized, observational cohort study in patients with chronic hepatitis C receiving peginterferon alfa/ribavirin combination therapy (ClinicalTrials.gov Identifier: NCT01344889). The first patient was enrolled on October 14, 2009 and the last patient completed follow-up on June 27, 2013. The study was registered with ClinicalTrials.gov on April 27, 2011. The delay in registration occurred because observational studies started before January 1, 2010 were not required to be registered. Thus, the decision to register the trial was taken on a voluntary basis. The authors confirm that all ongoing and related trials of peginterferon alfa-2a/ribavirin are registered. The study was conducted in outpatient hepatology clinics in 25 countries in Europe, Asia, North Africa, the Middle East, and South America.

### Patients

Adult patients (males and non-pregnant females) with chronic hepatitis C and quantifiable serum hepatitis C virus (HCV) RNA levels receiving treatment with peginterferon alfa/ribavirin according to the standard of care and the product license were eligible for enrollment after providing informed consent. Patients with contraindications to peginterferon alfa/ribavirin or with end-stage renal disease, and/or recipients of major organ transplants, were not eligible for the study. Enrollment in the study and the dose and duration of treatment were at the discretion of the investigator. Overall, 4453 patients were recruited into the GUARD-C program and a total of 3181 treatment-naive patients with HCV mono-infection were enrolled and followed up between October 2009 and June 2013 as follows: Albania (n = 46), Algeria (n = 15), Bahrain (n = 23), Belgium (n = 211), Bosnia-Herzegovina (n = 41), Brazil (n = 138), Egypt (n = 92), Greece (n = 175), India (n = 86), Iran (n = 60), Italy (n = 1001), Kuwait (n = 22), Lebanon (n = 28), Macedonia (n = 74), Pakistan (n = 90), Poland (n = 311), Portugal (n = 76), Qatar (n = 145), Romania (n = 333), Serbia (n = 50), Slovakia (n = 50), South Korea (n = 83), and the United Arab Emirates (n = 31). Additional sites in Hungary did not recruit any treatment-naive patients, and the study was suspended in Morocco before recruiting any patients.

The protocol conformed to guidelines for good pharmacoepidemiology practices [10] and the ethical guidelines of the 1975 Declaration of Helsinki. The protocol was approved by local independent ethics committees at each site. Full names of the approving ethics committees for each site can be found in S1 File. The ethics committees approved the protocol between August 14, 2009 and March 28, 2011. All patients provided informed written informed consent.

### Treatment

The planned treatment duration and dosing with peginterferon alfa/ribavirin were determined at the treating physician’s discretion according to the local standard of care and the local summary of product characteristics. Peginterferon alfa-2a solution is administered once-weekly by subcutaneous injection and ribavirin tablets are administered orally twice daily usually for a period of 24 or 48 weeks. Study drugs were self-administered by outpatients enrolled in the study.

### Assessments

Laboratory and clinical assessments were performed according to routine clinical practice and in accordance with the local standard of care. Safety and efficacy data were documented in an electronic case report form (eCRF) during treatment and follow-up.

### Outcomes

The primary efficacy endpoint was SVR24, defined as HCV RNA <50 IU/mL (as measured by a commercially available polymerase chain reaction assay) after 24 weeks of untreated follow-up (≥140 days after last dose of treatment). The analysis was conducted according to the intention-to-treat principle: patients with missing SVR24 values were considered nonresponders. Relapse was defined as HCV RNA ≥50 IU/mL during untreated follow-up in a patient with an end-of-treatment virologic response.

Rapid virologic response (RVR) was defined as a virologic response (HCV RNA <50 IU/mL) by Week 4. Complete early virologic response (cEVR) was defined as a virologic response by Week 12, but no RVR. Partial early virologic response (pEVR) was defined as a ≥2-log_10_ reduction in HCV RNA by Week 12 in a patient with no RVR or cEVR.

The primary safety endpoint of the trial was the time to first sr-RD of peginterferon alfa or ribavirin. The time will be calculated as days from first study treatment to the day of the first dose reduction or discontinuation due to safety reasons. If a patient discontinues the study treatment for any other reasons, then the time will be considered censored at the last treatment day. Patients were categorized by the time to first occurrence of sr-RD (no sr-RD or ≥1 sr-RD in the first 4 or 12 weeks of treatment) in order to assess the impact of sr-RDs on SVR24 rates.

### Statistical Analysis

It was assumed that the rate of sr-RD would be in the range of 20 to 40%; that the standard deviation of explanatory covariates included in the Cox proportional hazard model would range from 0.4 to 0.5 (after conversion of units) and that a risk reduction of 25% (equivalent to a hazard ratio of 0.75) for a 1-unit change in a covariate should be detected with 80% power. Since multiple covariates were to be included into the Cox regression model, an R^2^ of 0.1 to 0.2 was assumed for the multiple regression of a covariate on other covariates. On the basis of these assumptions, a sample size of 2500 evaluable patients would be required to detect a hazard ratio of 0.75 with 80% power at a significance level of 0.05. To account for patients with missing values, a sample size of 3000 patients was planned.

**Fig 1:**
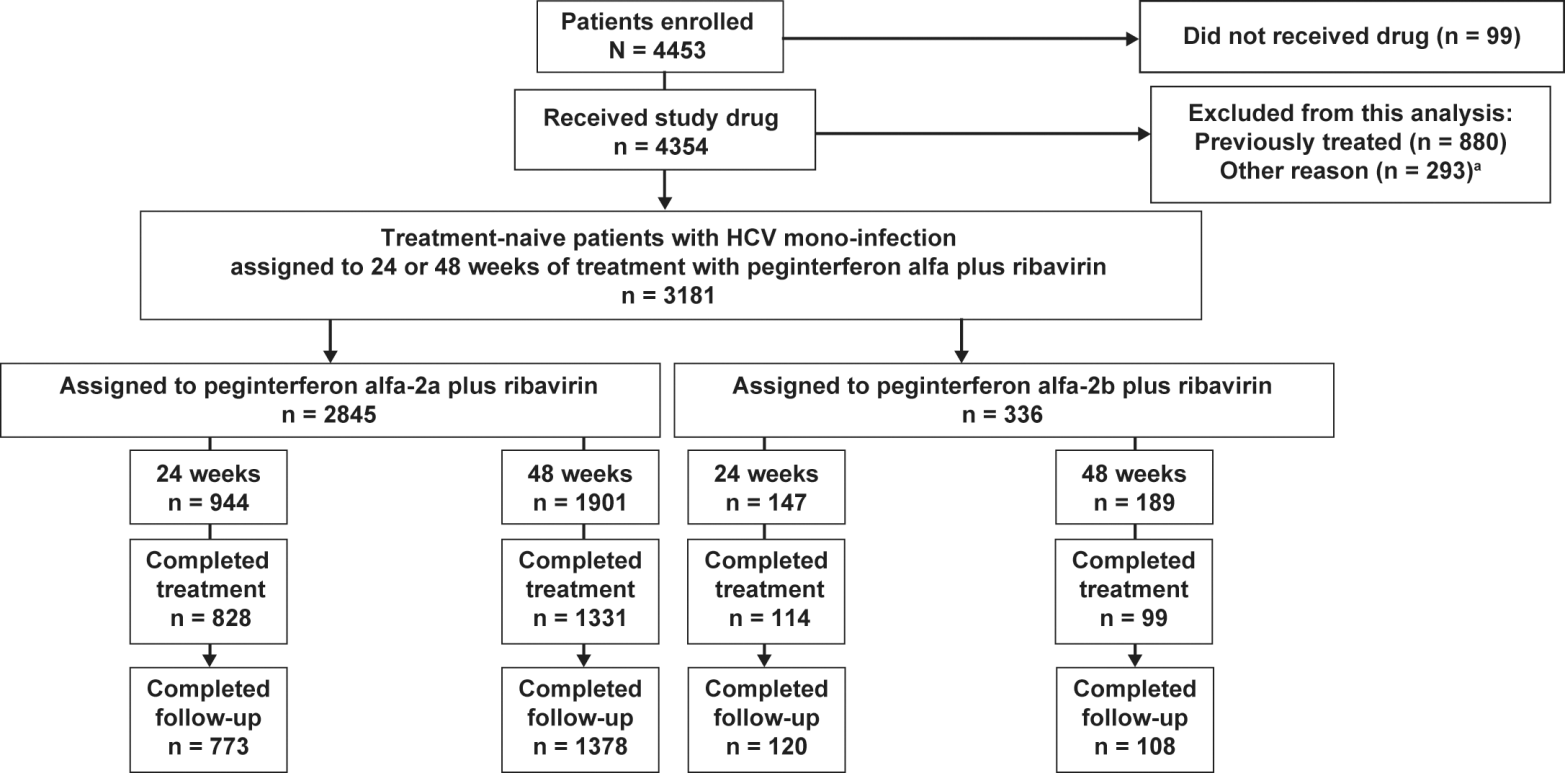
Enrollment and patient disposition. ^a^Other reasons (more than one reason may apply to a given patient): no final confirmation from the investigator (n = 56); contraindications to therapy (n = 15); HCV RNA-negative at screening/baseline (n = 12); end-stage renal disease (n = 7); major organ transplantation (n = 2); not treated with peginterferon alfa (n = 1) or ribavirin (n = 2); acute hepatitis C (n = 1); co-infection with HIV (n = 115); co-infection with HBV (n = 74); treatment with regimen other than peginterferon alfa-2a/ribavirin or peginterferon alfa-2b/ribavirin (n = 14); treatment-naive and intended treatment duration of 72 weeks (n = 6).

A total of 4453 patients were enrolled in GUARD-C and 4354 received at least one dose of study drug; however, this analysis was restricted to treatment-naive HCV mono-infected patients who were assigned to 24 or 48 weeks of treatment with peginterferon alfa-2a or -2b plus ribavirin (Fig 1). Patients with acute hepatitis C were excluded, as were those patients with chronic hepatitis C who were assigned to treatment durations longer than 48 weeks, those who had received prior treatment for chronic hepatitis C, those who had received other treatment regimens or had switched between treatment regimens, and/or those patients who were co-infected with hepatitis B virus or human immunodeficiency virus (HIV). Sub-analyses were performed in two sub-groups: 1) treatment-naive HCV mono-infected genotype (G) 1 patients (cirrhotic and noncirrhotic); and 2) treatment-naive HCV mono-infected Caucasian, noncirrhotic G1 patients, both of which populations were assigned to 48 weeks of treatment with peginterferon alfa-2a/ribavirin. The rationale for these sub-analyses is that G1 is the most common and difficult-to-treat HCV genotype, and that 48 weeks is the recommended duration of treatment with peginterferon alfa/ribavirin in patients with G1 infection [6]. A patient selection tool for Caucasian, noncirrhotic G1 patients treated for 48 weeks with peginterferon alfa-2a/ribavirin has been developed by Ferenci et al. [11]; thus, the analysis in subgroup 2 was intended to evaluate this patient selection tool in a separate population. Moreover, a meta-analysis has shown that there is a significant difference in SVR rates between patients treated with peginterferon alfa-2a and those treated with alfa-2b [12], and between different races (especially if host *IL28B* genotype is unknown) [13].

Subgroups considered in addition to the two subpopulations of G1 patients, included patients who did and did not receive at least 80% of the planned dose of peginterferon alfa-2a and ribavirin and who were treated for at least 80% of the planned treatment duration (“80/80/80”); and patients categorized by the time to first sr-RD using two different cutoffs (by Week 4 and by Week 12). Numbers and frequencies of variables of interest were calculated for subgroup analyses and associations between variables of interest (e.g. SVR) were investigated by tests of association (Pearson chi-square, Fisher’s exact test).

Cox proportional hazards analysis was used to identify baseline characteristics associated with time to the first sr-RD. Time to the first sr-RD was calculated in days from the first day of study treatment to the day at which the first sr-RD occurred. The time was censored at the last treatment day for patients who did not have any sr-RD.

The impact of sr-RD on SVR24 rates was assessed in subpopulation 2 (Caucasian, noncirrhotic G1 patients assigned to 48 weeks of peginterferon alfa-2a/RBV). In addition, the utility of a modified prediction tool for identifying Caucasian, noncirrhotic G1 patients with a high probability of achieving SVR24 with 48 weeks of treatment with peginterferon alfa-2a/ribavirin was assessed [11].

The modified scoring system involves five baseline factors (Table 1) and differs from that of Ferenci et al. [11] in that serum aspartate aminotransferase (AST) levels are not included because this parameter was not collected on the eCRF. The total score for an individual patient is the sum of the scores assigned for each of the five variables and ranges from 0–9, with higher scores indicating more favorable characteristics for achieving an SVR24. In this analysis, patients were categorized as having scores <5 and ≥5. A multiple logistic regression analysis was also performed to identify baseline and on-treatment factors associated with SVR24 in this population. Factors included in this analysis are listed in S1 Table. This analysis was performed after excluding patients who discontinued treatment for efficacy or other non-safety reasons, to avoid the misinterpretation that a shorter duration of treatment was responsible for the lack of an SVR24, when in fact the absence of early virological response was responsible for the short treatment duration.

**Table 1:** Scoring system used to identify patients in subgroup 2^a^ with a high or low probability of achieving SVR24.

Predictor		Score^b^
**Age, years**	≤35	2
	>35 but ≤45	1
	>45	0
**Body mass index, kg/m^2^**	≤20	2
	>20 but ≤22	1
	>22	0
**HCV RNA, IU/mL**	≤100,000	3
	>100,000 but ≤400,000	2
	>400,000 but ≤800,000	1
	>800,000	0
**Platelet count, x 10^9^/L**	>150	1
	≤150	0
**ALT/ULN^c^**	>3	1
	≤3	0

^a^HCV G1, treatment-naive, Caucasian, noncirrhotic patients assigned to 48 weeks of treatment with peginterferon alfa-2a.

^b^The total score for an individual patient is the sum of the scores assigned for each of the five variables and ranges from 0–9 with higher scores indicating more favorable characteristics. If one or more variable scores are missing for a patient, the total score is missing.

^c^Alanine aminotransferase (ALT) value divided by the upper limit of normal (ULN) for the local laboratory.

The analyses were done with SAS version 8.2 software (SAS Institute Inc., Cary, NC, USA) with the exception of the exploratory analyses. These were performed with SAS version 9.2 software.

## Results

### Patients

Overall, a total of 3181 treatment-naive patients with HCV mono-infection were enrolled in 25 countries, assigned to a planned treatment duration of 24 or 48 weeks and treated with peginterferon alfa/ribavirin and followed up between October 2009 and June 2013 (Fig 1). The baseline characteristics for all patients are shown in S2 Table (patients treated with peginterferon alfa-2a/ribavirin) and S3 Table (patients treated with peginterferon alfa-2b/ribavirin).

The majority of patients had HCV G1 infection (1634/3181; 51.7%)–92% of whom (1497/1634) were assigned to 48 weeks of treatment with peginterferon alfa-2a/ribavirin (Table 2). The mean age of these patients was 47.4 years and the majority were male (53.8%), Caucasian (92.7%), and had a baseline HCV RNA level >800,000 IU/mL (53.1%). Approximately 30% of these patients had transition to cirrhosis or cirrhosis, and 19% and 3% had a history of cardiovascular and pulmonary disease, respectively.

**Table 2:** Baseline characteristics of patients included in the analyses.

Characteristic	All patients	Genotype 1 patients assigned to peginterferon alfa-2a/RBV for 48 weeks	
		Subgroup 1 All	Subgroup 2 Noncirrhotic, Caucasian
	N = 3181	n = 1497	n = 951
**Male sex, n (%)**	1930 (60.7)	806 (53.8)	519 (54.6)
**Race, n (%)**			
Caucasian/White	2767 (87.0)	1388 (92.7)	951 (100)
Black	60 (1.9)	20 (1.3)	0
Asian/Oriental	340 (10.7)	76 (5.1)	0
Other	14 (0.4)	13 (0.9)	0
**Mean age ± SD, years**	46.0 ± 12.8	47.4 ± 12.5	45.1 ± 12.8
**Mean body mass index ± SD, kg/m^2^**	26.0 ± 4.3	25.9 ± 4.2	25.7 ± 4.0
**Method to assess liver fibrosis**			
Biopsy	1479 (46.5)	915 (61.1)	570 (59.9)
Noninvasive	907 (28.5)	343 (22.9)	206 (21.7)
Best guess/Not assessed	795 (25.0)	239 (16.0)	175 (18.4)
**Patients with cirrhosis/transition to cirrhosis^a^**	758/3174 (23.9)	460/1494 (30.8)	0
**Mean HCV RNA ± SD, log_10_ IU/mL**	5.78 ± 0.86	5.87 ± 0.79	5.89 ± 0.77
**HCV RNA >800,000 IU/mL, n (%)**	1575 (50.2)	790 (53.1)	506 (53.7)
**History of cardiovascular disease, n (%)**	488 (15.3)	286 (19.1)	144 (15.1)
**History of pulmonary disease, n (%)**	96 (3.0)	46 (3.1)	24 (2.5)
**History of diabetes mellitus, n (%)**	282 (8.9)	139 (9.3)	57 (6.0)
**History of thyroid disease, n (%)**	175 (5.5)	92 (6.1)	57 (6.0)
**History of psychiatric disease/symptoms, n (%)**	233 (7.3)	103 (6.9)	54 (5.7)
**Mean hemoglobin concentration ± SD, g/L**	151.1 ± 13.8	151.6 ± 13.5	152.0 ± 13.2
**Mean neutrophil count ± SD x 10^9^/L**	3.6 ± 1.7	3.6 ± 1.8	3.7 ± 1.4
**Mean platelet count ± SD x 10^9^/L**	205.0 ± 67.6	203.8 ± 67.2	216.5 ± 61.2
**Mean ALT activity ± SD, IU/L**	118.3 ± 99.6	116.5 ± 95.7	109.7 ± 92.9
**Mean ALT ratio ± SD**	2.15 ± 1.81	2.12 ± 1.74	2.00 ± 1.69
**HCV genotype, n (%)**			
1	1634 (51.7)	1497 (100)	951 (100)
2	362 (11.5)	0	0
3	756 (23.9)	0	0
4	396 (12.5)	0	0
5/6	10 (0.3)	0	0
**Planned treatments, n (%)**			
PegIFN alfa-2a/RBV	2845 (89.4)	1497 (100)	951 (100)
PegIFN alfa-2b/RBV	336 (10.6)	-	-
24 weeks	1091 (34.3)	-	-
48 weeks	2090 (65.7)	1497 (100)	951 (100)

^a^Assessed by biopsy, noninvasive testing, or best guess.

ALT, alanine aminotransferase; SD, standard deviation.

The baseline characteristics of the 951 patients in subgroup 2 were similar to those in the overall G1 population with respect to sex, age, weight, body mass index, HCV RNA level, and laboratory test results (Table 2).

The number of patients in whom treatment with peginterferon alfa and ribavirin was prematurely withdrawn is shown in S4 Table for the total patient population (25.4% and 26.0%, respectively), subgroup 1 (28.7% and 29.2%, respectively), and subgroup 2 (24.8% and 25.2%, respectively). Insufficient therapeutic response was the most common reason for premature withdrawal from peginterferon alfa-2a in the total patient population (9.5%), and in subgroups 1 (13.3%) and 2 (10.4%). Rates of premature withdrawal from treatment with peginterferon alfa and ribavirin for safety reasons in the total patient population (5.5% and 6.0%, respectively), and in subgroup 1 (6.4% and 6.9%, respectively) and subgroup 2 (5.6% and 6.0%) were similar.

### Efficacy: SVR24 Rates

In the overall population, stratified by HCV genotype, the SVR24 rates for patients with HCV G1, 2, 3, and 4 infection were 46.1% (754/1634), 77.1% (279/362), 68.0% (514/756), and 51.3% (203/396), respectively. Virologic response and relapse rates by treatment regimen and genotype are presented in S5 and S6 Tables.

In the overall population, SVR24 rates were higher in patients who received at least 80% of the planned dose of peginterferon alfa-2a and ribavirin and who were treated for at least 80% of the planned treatment duration (“80/80/80”) (1496/2182, 68.6%) than in those who did not fulfill the “80/80/80” rule (279/998, 28.0%).

Among the 951 patients in subgroup 2, the SVR24 rate was 52.9% (503/951). For those who fulfilled the “80/80/80” rule, the SVR24 rate was 66.0% (426/645). In contrast, the SVR24 rate was 25.2% (77/305) in patients who did not meet these criteria (exposure data were unavailable for one patient) (Pearson chi-square P<0.0001). However, this latter group includes patients who discontinued treatment prematurely for any reason and, in particular, patients who discontinued treatment for lack of efficacy.

### Safety-Related Dose Reductions or Discontinuations (sr-RD)

The incidences of sr-RD of peginterferon alfa in the overall population, and in subgroups 1 and 2 were 16.9%, 21.3%, and 18.4%, respectively, and the incidences of sr-RD for ribavirin were 21.8%, 29.5%, and 28.5%, respectively (S7 Table). The most common causes of a first sr-RD of peginterferon alfa-2a were neutropenia (6.9%, 9.2%, and 8.1%, respectively), thrombocytopenia (3.5%, 5.3%, and 3.7%, respectively), anemia (1.2%, 1.1%, and 0.9%, respectively), and asthenia (1.0%, 1.5%, and 1.5%, respectively), and the most common causes of a first sr-RD of ribavirin were anemia (13.1%, 18.8%, and 18.2%, respectively), decreased weight (2.0%, 3.1%, and 3.4%, respectively), and asthenia (0.9%, 1.1%, and 1.2%, respectively).

**Fig 2:**
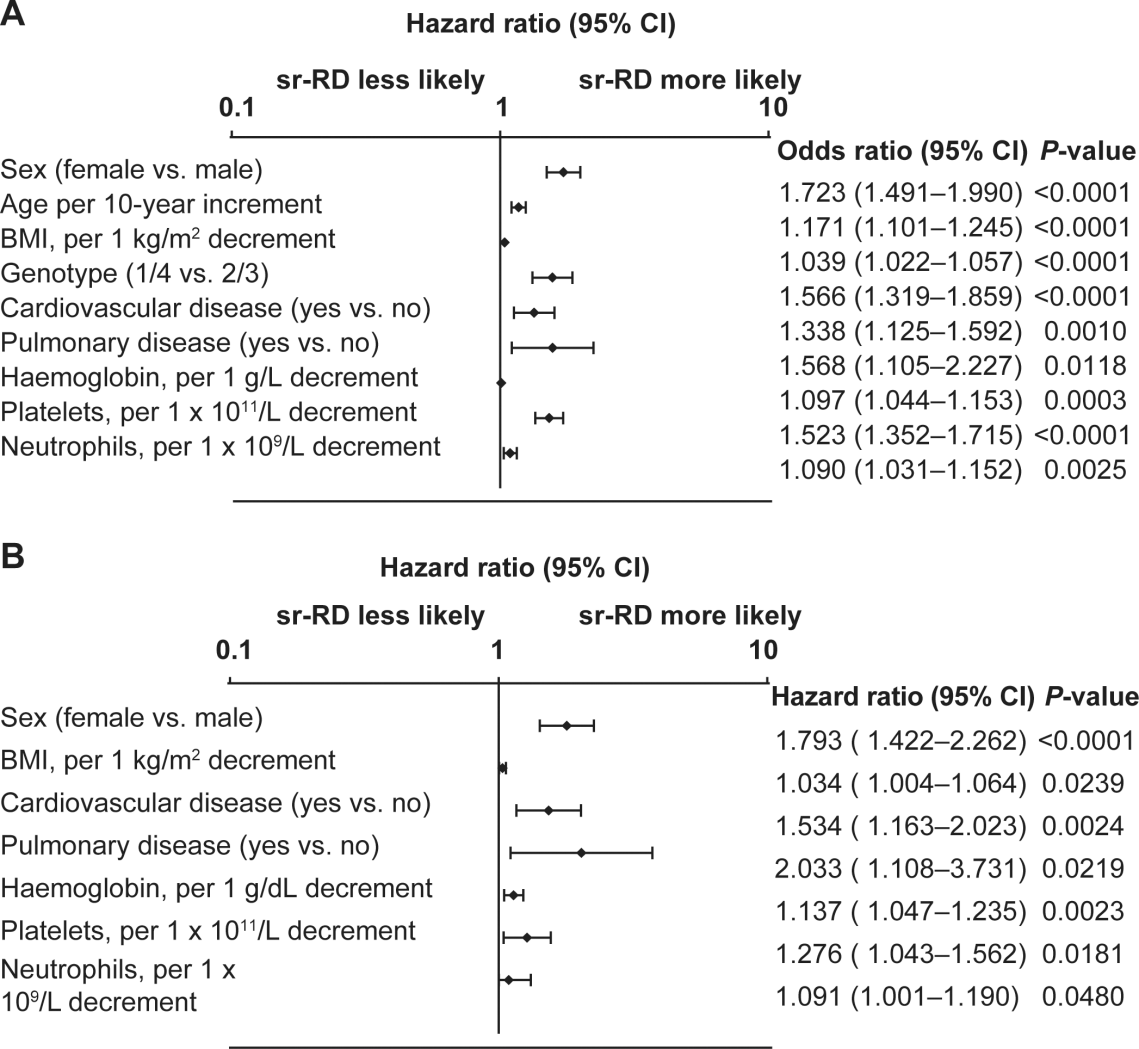
Cox proportional hazards analysis for time to first safety-related dose reductions or discontinuations in patients treated for 24 or 48 weeks with peginterferon alfa-2a or alfa-2b and ribavirin. (A) All treatment-naive patients (G1–6) assigned to 24 or 48 weeks of treatment with peginterferon alfa-2a or alfa-2b/RBV (N = 3181); (B) Subgroup 2: treatment-naive Caucasian, G1 noncirrhotic patients assigned to 48 weeks of treatment with peginterferon alfa-2a/RBV (n = 951).

**Fig 3:**
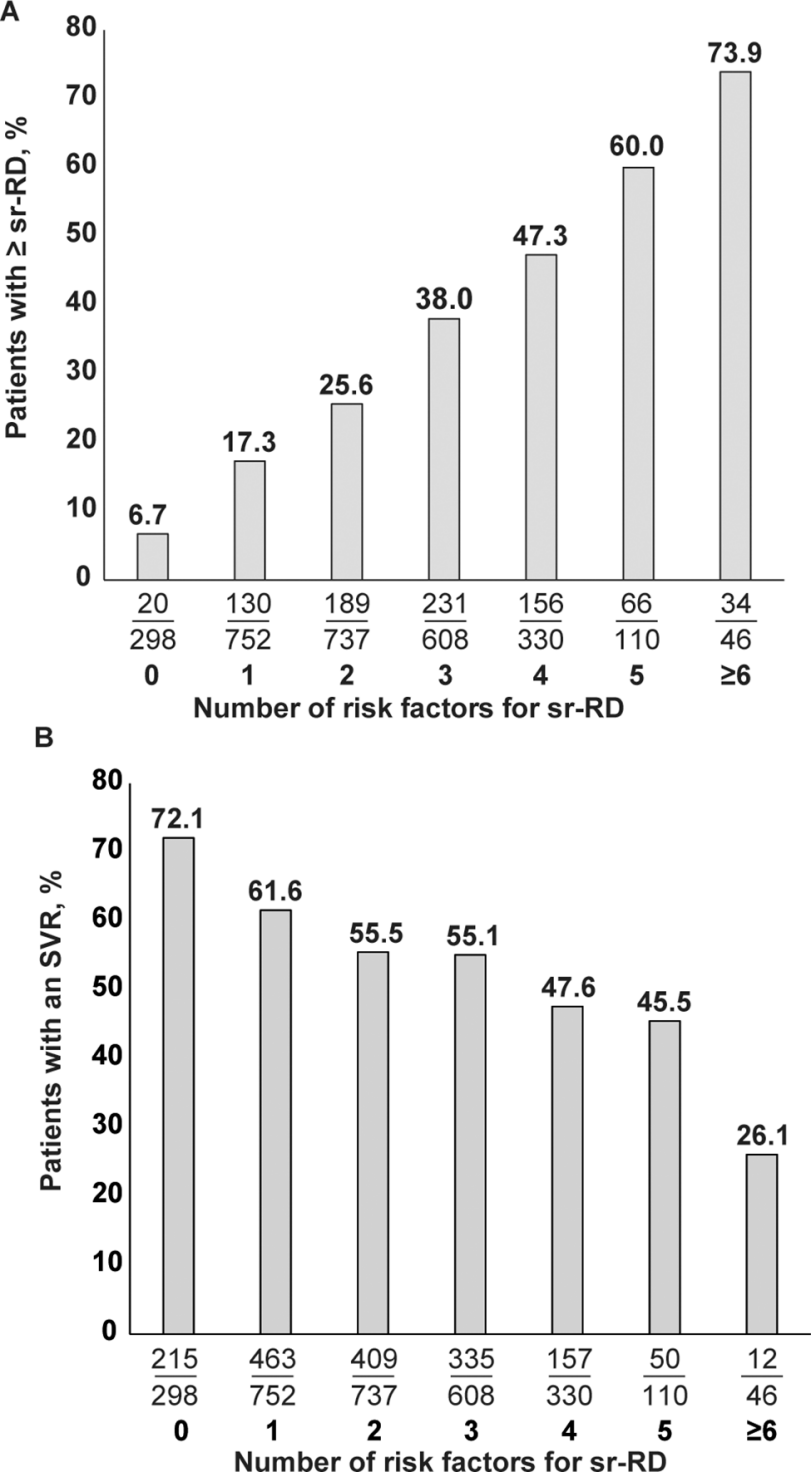
Incidence of safety-related dose reductions or discontinuations (sr-RD) (A) and SVR24 rates (B) according to the number of baseline risk factors for sr-RD in the overall population (N = 2881). Patients with missing data (n = 300) for risk factors were excluded (BMI n = 18; HCV genotype n = 23; baseline hemoglobin n = 45; baseline platelets n = 56; baseline neutrophils n = 245). Baseline risk factors for sr-RD based on results of Cox proportional hazards analysis include: female sex, age >50 years, body mass index ≤22 kg/m^2^, HCV G1 or 4 infection, presence of cardiovascular disease, presence of pulmonary disease, hemoglobin ≤140 g/L, platelets ≤115 x 10^9^/L, neutrophils ≤2.0 x 10^9^/L.

Baseline factors significantly associated with earlier occurrence of a first sr-RD of peginterferon or ribavirin in the overall population included female sex, older age, lower body mass index, G1/4 vs. 2/3, presence of pre-existing cardiovascular or pulmonary diseases, lower hemoglobin concentration, and lower platelet and neutrophil counts (Fig 2A). The incidence of sr-RD increased in proportion to the number of baseline risk factors for sr-RD (Fig 3A). Conversely, the SVR24 rate was highest in patients without risk factors for sr-RD (72.1%) and lowest in patients with ≥6 risk factors for sr-RD (26.1%, Fig 3B).

Baseline factors associated with time to first sr-RD in subgroup 2 were similar to those in the overall population with the exception of genotype (not applicable) and age (Fig 2B).

Exposure to treatment and the time of occurrence of sr-RD have an impact on SVR rates as shown in Table 3. When the timing of the first sr-RD was superimposed on the “80/80/80” rule there was a substantial difference in SVR rates, with the higher SVR rates in patients who did not have an early dose reduction. For example, among patients who fulfilled the “80/80/80” rule SVR rates are higher in those who did not have an sr-RD in the first 4 weeks (68.9% vs. 60.3%) or first 12 weeks (69.7% vs. 57.4%).

**Table 3:** SVR rates according to exposure and time of first sr-RD in the overall population.

Patient subgroup		“80/80/80” rule fulfilled (N = 2182)^a^		“80/80/80” rule NOT fulfilled (n = 998)^b^	
		SVR	No SVR	SVR	No SVR
**sr-RD in first 4 weeks**	**No**	1452/2109 (68.9)	657/2109 (31.1)	228/836 (27.3)	608/836 (72.7)
	**Yes**	44/73 (60.3)	29/73 (39.7)	51/162 (31.5)	111/162 (68.5)
**sr-RD in first 12 weeks**	**No**	1384/1987 (69.7)	603/1987 (30.3)	169/674 (25.1)	505/674 (74.9)
	**Yes**	112/195 (57.4)	83/195 (42.6)	110/324 (34.0)	214/324 (66.0)

^a^Received ≥80% of planned drug dosages and were treated for at least 80% of the planned duration.

^b^Received <80% of planned drug dosages and/or were treated for less than 80% of the planned duration.

### Impact of sr-RD on SVR24 Rates in HCV G1 Patients Treated for 48 Weeks With Peginterferon Alfa-2a/Ribavirin (Subgroups 1 and 2)

**Fig 4:**
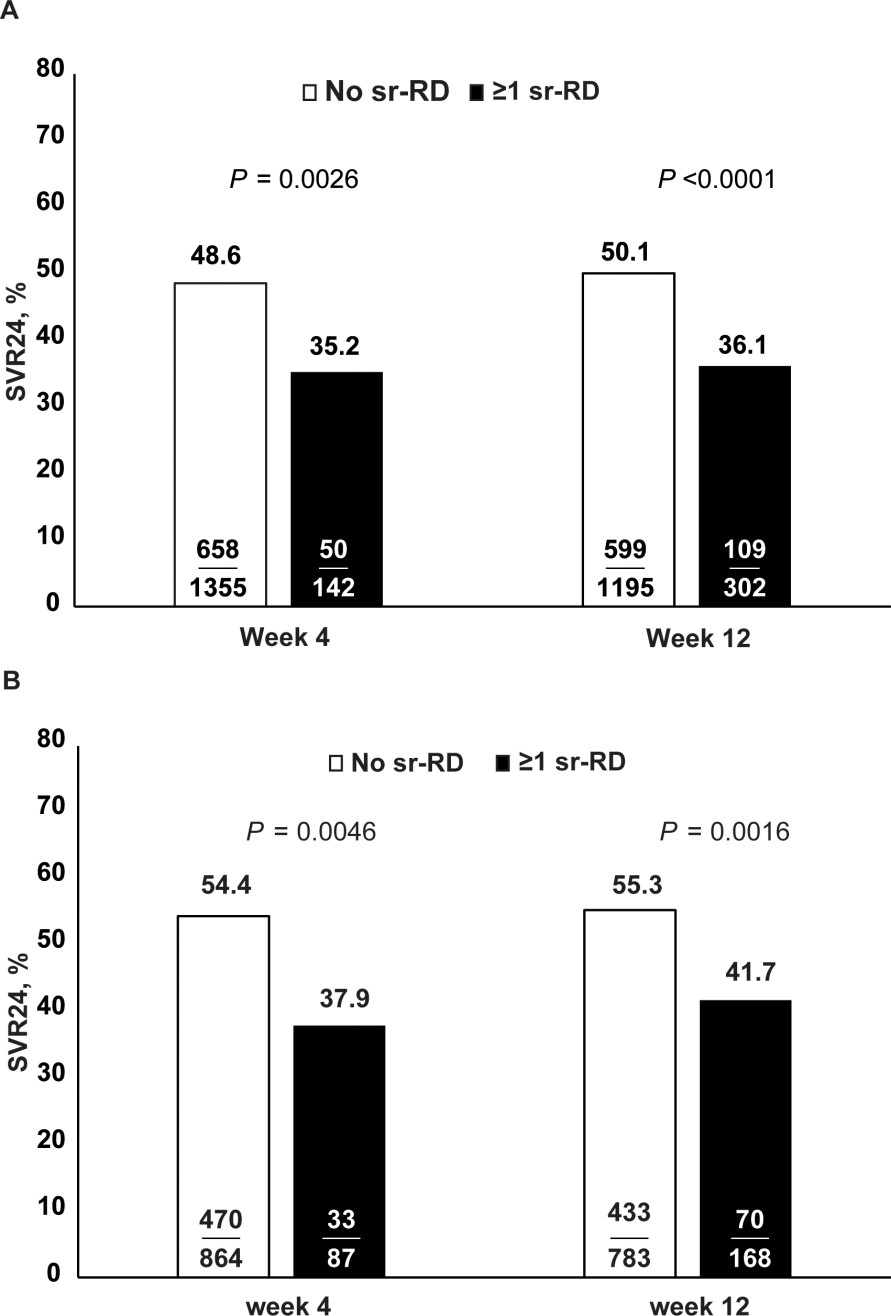
SVR24 rates by incidence of safety-related dose reductions or discontinuations by Week 4 and Week 12 of treatment in genotype 1 patients assigned to 48 weeks of treatment with peginterferon alfa-2a/ribavirin. Fisher’s exact test, two-sided P-value. (A) Subgroup 1: all treatment-naive genotype 1 patients (n = 1497); (B) Subgroup 2: treatment-naive, genotype 1, noncirrhotic Caucasian patients (n = 951).

Among the patients in subgroup 1, the SVR24 rate was significantly higher than in those who did not report an sr-RD by Week 4 of treatment (48.6% vs. 35.2% in patients with ≥1 sr-RD by Week 4, P = 0.0026) or by Week 12 of treatment (50.1% vs. 36.1% ≥1 sr-RD by Week 12, P<0.0001) (Fig 4A).

Similarly, among patients in subgroup 2, the SVR24 rate was significantly higher in those with no sr-RD by Week 4 or 12 than in those with at least one sr-RD (by Week 4: 54.4% vs. 37.9%, P = 0.0046; by Week 12: 55.3% vs. 41.7%, P = 0.0016) (Fig 4B). SVR24 rates in patients with the first sr-RD in Weeks 0–4, >4–12, and >12 weeks were 37.9% (33/87), 45.7% (37/81), and 61.9% (104/168), respectively.

### SVR24 Rates by Baseline Score (Subgroup 2)

**Fig 5:**
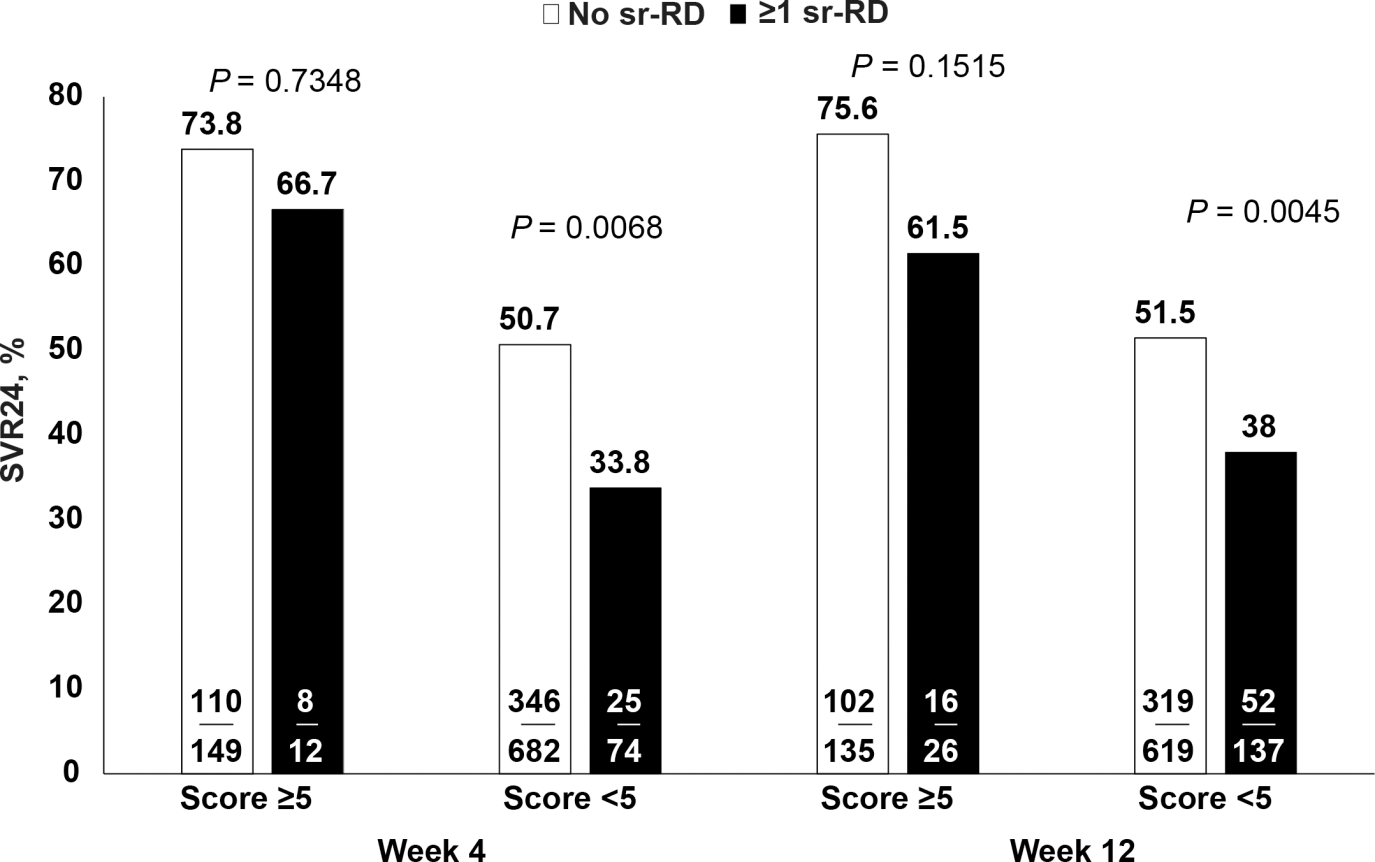
SVR24 rates by baseline predictive score and by incidence of safety-related dose reductions or discontinuations by Week 4 and Week 12 of treatment in subgroup 2 (Caucasian, treatment-naive, G1 noncirrhotic patients assigned to 48 weeks of treatment with peginterferon alfa-2a/ribavirin). Fisher’s exact test, two-sided P-value. Please note that 34 patients had unknown baseline score.

Among the 951 patients included in subgroup 2, the baseline scoring system was useful in stratifying patients with a low and high chance of achieving SVR24 (S7 Table). A total of 161 and 756 individuals had baseline predictive scores ≥5 and <5, respectively. As expected, the SVR24 rate was higher in patients with more favorable predictive scores (≥5) than in those with less favorable predictive scores (<5) (73.3% [118/161] vs. 49.1% [371/756], P<0.0001). Among patients with predictive scores ≥5, SVR24 rates were numerically but not statistically significantly higher in patients who experienced no sr-RD in the first 4 or 12 weeks than in patients who experienced ≥1 sr-RD in the first 4 (73.8% vs. 66.7%) or 12 weeks (75.6% vs. 61.5%) (Fig 5). However, among patients with baseline scores <5, SVR24 rates were significantly higher in those who experienced no sr-RD in the first 4 or 12 weeks than in patients who experienced ≥1 sr-RD by Week 4 (50.7% vs. 33.8%, P = 0.0068) and by Week 12 (51.5% vs. 38.0%, P = 0.0045) (Fig 5).

SVR rates were also higher in patients with baseline scores of ≥5 than <5 regardless of whether the “80/80/80” rule was fulfilled (Table 4). Thus, in patients fulfilling the “80/80/80” rule, SVR rates were 83.0% in those with a baseline prediction score ≥5 and 63.1% in those with a prediction score <5. In patients who did not fulfill the “80/80/80” rule, SVR rates were 51.0% and 20.3% respectively.

**Table 4:** SVR rates according to exposure and baseline prediction score in subgroup 2.

Patient subgroup		“80/80/80” rule fulfilled (N = 621)^a^		“80/80/80” rule not fulfilled (n = 295)^b^	
		SVR	No SVR	SVR	No SVR
**Baseline prediction score**	**<5**	321/509 (63.1)	188/509 (36.9)	50/246 (20.3)	196/246 (79.7)
	**≥5**	93/112 (83.0)	19/112 (17.0)	25/49 (51.0)	24/49 (49.0)

^a^Received ≥80% of planned drug dosages and were treated for at least 80% of the planned duration)

^b^Received <80% of planned drug dosages and/or were treated for less than 80% of the planned duration).

**Fig 6:**
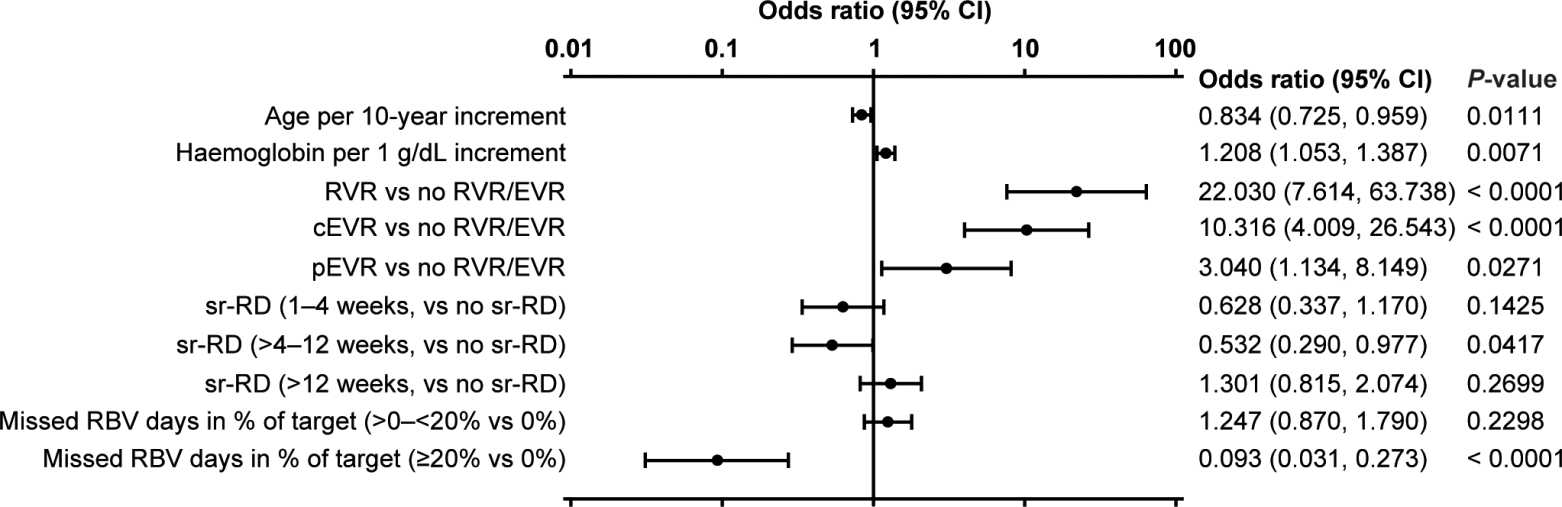
Multiple logistic regression analysis of baseline and on-treatment factors associated with SVR24 in Caucasian, treatment-naive, G1, noncirrhotic patients assigned to 48 weeks of treatment with PegIFN alfa-2a/RBV. Patients who discontinued therapy for efficacy or other non-safety reasons were excluded. ^a^Effect P-value: P = 0.0316 for sr-RD and 0.0001 for missed ribavirin days in percentage of target.

Early sr-RDs (i.e. within the first 12 weeks) were associated with lower SVR24 rates in this population (*P* = 0.0316 for overall effect; Fig 5) (patients who discontinued for non-safety reasons were excluded from the logistic regression analysis). Other factors significantly associated with lower SVR24 rates included older age, lower baseline hemoglobin concentration, no on-treatment virologic response (no RVR, cEVR, or pEVR), and more than 20% missed days of treatment with ribavirin (Fig 6).

### Safety

Among the 3181 treatment-naive patients the overall incidence of adverse events (AEs) and serious adverse events (SAEs) was 71.1% and 5.9%, respectively (S8 Table). A total of 11 patients (0.3%) died during the study. The most common AEs were anemia (25.1%), asthenia (16.2%), decreased weight (15.6%), neutropenia (15.2%), and thrombocytopenia (12.7%) (S9 Table).

The adverse event profile in treatment-naive G1 patients assigned to 48 weeks of treatment with peginterferon alfa-2a/ribavirin profile was similar to that of the overall population, although the incidence of AEs, SAEs, and individual event rates were somewhat higher. The incidences of anemia, asthenia, decreased weight, neutropenia, and thrombocytopenia were 34.5%, 21.1%, 20.3%, 21.4%, and 20.0% respectively (S9 Table).

## Discussion

The safety and efficacy of dual peginterferon alfa/ribavirin therapy observed in GUARD-C are consistent with the results of previous large randomized trials and cohort studies. The overall SVR24 rates of 55.8% (all genotypes combined) and 46.1% in the G1 population is very similar to that achieved in the Phase 3 registration trials [14–16]. The higher SVR24 rate in G1 patients assigned to 48 weeks’ treatment with peginterferon alfa-2a/ribavirin (47.3%) than in those receiving peginterferon alfa-2b/ribavirin (28.8%) supports the results of a comprehensive meta-analysis of randomized trials [12]. The overall rate of treatment withdrawal among G1 patients assigned to 48 weeks of treatment with peginterferon alfa-2a/ribavirin was approximately 25% in the present study, which is similar to the rate reported in Phase 3 studies of peginterferon alfa-2a/ribavirin (≈30%) [15,16]. In this subgroup, the most common reasons for withdrawal of treatment with peginterferon alfa-2a in the present study were lack of efficacy (≈10%) and safety (≈6%).

Importantly, this analysis from GUARD-C shows that baseline factors can be used to identify patients at increasing risk for sr-RD, and that sr-RD has an impact on SVR24 rates. Factors associated with sr-RD in the overall population (G1–6) included female sex, older age, lower body mass index, HCV G1/4 vs. 2/3, the presence of cardiovascular and pulmonary disease and low hematological indices (hemoglobin, neutrophils, platelets). The same factors were associated with sr-RD in Caucasian noncirrhotic HCV G1 patients with the exception of age (and genotype). To our knowledge, this association between sr-RD and pre-existing pulmonary and cardiovascular disease is a novel finding.

Not surprisingly, the incidence of sr-RD increased in proportion to the number of risk factors for sr-RD. Among patients with no risk factors the incidence of sr-RD was approximately 7% and among those individuals with at least six risk factors for sr-RD the incidence was approximately 74%. Conversely, SVR24 rates were highest in patients with no risk factors for sr-RD (72.1%) and were lowest in patients with at least six risk factors for sr-RD (26.1%).

There is a broad overlap between risk factors for sr-RD and factors predictive of lower SVR24 rates. For example, here G1 or 4 infection was identified as a predictor of higher sr-RD rates, while it is also a well-established predictor of lower SVR24 rates. Consistently lower SVR24 rates are achieved in patients infected with G1 or 4 as compared with G2 or 3 [6]. Increasing age was associated with increasing sr-RD rates in this analysis and has been associated with lower SVR24 rates [17]. Conversely, here, higher body mass index was associated with lower sr-RD rates, while higher body mass index is typically associated with lower SVR24 rates [18]. This may be the result of lower exposure to drug in heavier patients. A higher incidence of certain hematological events with increased exposure to ribavirin and peginterferon alfa-2a has been reported elsewhere [19].

On the basis of this analysis, a physician will be able to evaluate a patient for peginterferon alfa-2a therapy by considering, on the one hand, a set of factors associated with efficacy (SVR) and, on the other hand, a set of factors associated with safety (sr-RD). Unique factors related to sr-RD identified in this analysis include hematological parameters and the presence of cardiovascular and pulmonary disease.

Hematological abnormalities are important adverse events associated with peginterferon alfa/ribavirin treatment. Anemia was the most common adverse event reported during the present study and was the most common event associated with a first sr-RD of ribavirin. Neutropenia, thrombocytopenia, and anemia were the three most common events associated with a first sr-RD of peginterferon alfa in the overall analysis. Moreover, decreasing hemoglobin, platelet, and neutrophil levels at baseline were all significantly associated with earlier occurrence of sr-RD in the Cox proportional hazards analysis, both in the overall population and in the subgroup of noncirrhotic Caucasian patients with HCV G1 infection.

The relationship between hematological parameters and safety and efficacy outcomes is complex. Anemia is a common complication of dual peginterferon alfa/ribavirin treatment. For example, approximately one-third of patients have hemoglobin levels <10 g/dL at some point during a 48-week course of treatment [20]. Anemia is a common cause of dose reductions, particularly of ribavirin, and reduced exposure to ribavirin as a result of physician-initiated dose reductions and non-adherence by patients is associated with significant reductions in SVR24 rates [7–9].

Paradoxically, patients with large decreases in hemoglobin (e.g. >3 g/dL) have higher SVR24 rates than patients with lesser decreases in hemoglobin [9,21]. It has been suggested that the drop in hemoglobin is a marker of the strength of the response to treatment. This interpretation is supported by data that show that marginal decreases in platelet and white blood cell counts during treatment are associated with nonresponse [22].

The patient selection (SVR24 predictor) tool used in this analysis was based on one developed using data from Caucasian, treatment-naive G1 patients enrolled in a large noninterventional observational study (PROPHESYS) [11]. We included all parameters used in the original version [11] with the exception of AST, which was not recorded on the eCRF in our study. The incorporation of the predictive score in the current analysis shows that the impact of early sr-RD on SVR24 rates is most important in patients with an unfavorable baseline predictive score. For example, the occurrence of ≥1 sr-RD in the first 4 weeks of treatment in a patient with an unfavorable baseline score (<5) was associated with a significant reduction in the SVR24 rate (from 50.7% in patients with no sr-RD to 33.8% in patients with ≥1 sr-RD). In contrast, among patients with a favorable baseline score (≥5), the SVR24 rates in patients with an sr-RD (66.7%) and without one (73.8%) in the first 4 weeks of treatment were not significantly different. This may indicate that, in patients with favorable characteristics, dose reductions have only a minimal impact upon response rates.

The multiple logistic regression analysis of factors associated with SVR24 shows that achievement of an on-treatment virologic response (RVR or cEVR) was the most important positive predictor of SVR24. The occurrence of sr-RD during Weeks >4–12 was a significant negative predictor of SVR24. The occurrence of a first sr-RD in the first 4 weeks of treatment showed a similar trend but was not significant, which could be due to the small number of patients and slightly different response rates.In contrast, a first occurrence of an sr-RD after Week 12 was not a negative predictor for SVR24. Furthermore, missing ≥20% of the targeted days of treatment with ribavirin had a marked negative association with achievement of SVR24, while missing <20% of the targeted days of treatment with ribavirin did not have a negative effect on SVR24. This suggests that longer treatment interruptions or early permanent discontinuations due to safety reasons have a strong impact on SVR24 rates, while early dose reductions or short interruptions are less critical, subject to the condition that the patient received treatment for more than 80% of the target treatment days.

Patient selection is increasingly important in the treatment of chronic hepatitis C. Several DAAs are now available, and interferon-free regimens with consistently high cure rates are now a reality [2]. However, although recently approved and pending DAA-containing regimens are highly effective and have good safety profiles, they are unlikely to be universally available due to high acquisition costs [2,3,5]. For this reason dual peginterferon/ribavirin therapy is likely to remain a viable option in resource-limited settings. For clinicians who continue to use dual therapy in their practice, it is important that they remain able to make informed decisions about the suitability of treatment for a given patient. This analysis provides practical information that can be used to inform such decisions. An individual patient’s characteristics can be reviewed prior to baseline to evaluate whether they are likely to achieve an SVR24 with dual therapy, and whether they are likely to experience an sr-RD.

Limitations of this study include those typical of cohort studies and retrospective analyses. The number of non-Caucasian patients and patients with frank cirrhosis was low, a fact which places limits on the generalizability of the results. Host *IL28B* genotype is the most important baseline predictor of response to peginterferon alfa [12]. As such, host genotype provides very useful information when planning treatment with peginterferon alfa/ribavirin; however, *IL28B* genotype is not routinely available in resource-limited settings where dual therapy is most likely to be used. The patient selection tool of Ferenci et al. [11] does not include liver biopsy results, and analysis of subgroup 2 in the present study specifically excluded patients with cirrhosis. However, as patients with cirrhosis are a group in urgent need of treatment, it seems reasonable to say that these individuals should be given a high priority for treatment with DAA-containing regimens including interferon-free combinations, regardless of their baseline predictive score. Our application of the prediction score by Ferenci et al. [11] did not include one of the prediction factors (serum AST ratio at baseline); however, the modified score was still able to differentiate patients with high and low chances of SVR.

In conclusion, the results of GUARD-C show that it is possible to identify patients at risk of sr-RD by using simple and readily available data regarding baseline characteristics, and that it is possible to predict the extent of the risk for sr-RD. sr-RDs have a negative impact on the probability of achieving an SVR, especially if they occur within the first 12 weeks of treatment. Moreover, the results suggest that early dose reductions or interruptions are less critical than longer interruptions or permanent discontinuations for achievement of SVR24 in noncirrhotic Caucasian patients with G1 infection, provided that ribavirin is administered for >80% of the targeted number of treatment days. Finally, the modified baseline score will help to identify those patients with a high likelihood for SVR. The results will be of particular interest to clinicians with limited access to DAAs and those who continue to use dual therapy with peginterferon alfa-2a/ribavirin.

## Supporting Information

S1 File Ethics Committees. (DOCX)

S2 File Protocol. (PDF)

S3 File STROBE Checklist. (DOCX)

S4 File TREND statement. (DOCX)

S1 Table Factors considered in the multiple logistic regression analysis of SVR. (DOCX)

S2 Table Baseline characteristics of 2845 treatment-naive HCV mono-infected patients treated with peginterferon alfa-2a/ribavirin. (DOCX)

S3 Table Baseline characteristics of 336 treatment-naive HCV mono-infected patients treated with peginterferon alfa-2b/ribavirin. (DOCX)

S4 Table Reasons for premature withdrawal: treatment-naive HCV mono-infected patients treated with peginterferon alfa/ribavirin. (DOCX)

S5 Table Virologic response in 2845 treatment-naive HCV mono-infected patients treated with peginterferon alfa-2a/ribavirin. (DOCX)

S6 Table Virologic response in 336 treatment-naive HCV mono-infected patients treated with peginterferon alfa-2b/ribavirin. (DOCX)

S7 Table Distribution of patients and SVR24 rates according to baseline score. (DOCX)

S8 Table Reasons for first safety-related dose reduction or discontinuation (sr-RD): treatment-naive HCV mono-infected patients treated with peginterferon alfa/ribavirin. (DOCX)

S9 Table Safety: treatment-naive HCV mono-infected patients treated with peginterferon alfa/ribavirin. (DOCX)

## Abbreviations

cEVR: complete early virologic response
DAA: direct-acting antiviral
eCRF: electronic case report form
G: genotype
HCV: hepatitis C virus
pEVR: partial early virologic response
RVR: rapid virologic response
sr-RD: safety-related dose reductions or discontinuations
SVR: sustained virologic response

## References

[1] American Association for the Study of Liver Diseases and Infectious Diseases Society of America. Recommendations for testing, managing, and treating hepatitis C. Available: http://www.hcvguidelines.org. Accessed 24 Aug 2014. 10.1002/hep.31060 PMC9710295 31816111

[2] Hoofnagle JH, Sherker AH. Therapy for hepatitis C—the costs of success. N Engl J Med 2014;370:1552–1553. 10.1056/NEJMe1401508 24725236

[3] Muir AJ. The rapid evolution of treatment strategies for hepatitis C. Am J Gastroenterol 2014;109:628–635. 10.1038/ajg.2014.66 24732866

[4] Anonymous. Only just the beginning of the end of hepatitis C. Lancet 2014;383:281 10.1016/S0140-6736(14)60087-8 24461110

[5] Jayasekera CR, Barry M, Roberts LR, Nguyen MH. Treating hepatitis C in lower-income countries. N Engl J Med 2014;370:1869–1871. 10.1056/NEJMp1400160 24720680

[6] Ghany MG, Strader DB, Thomas DL, Seeff LB. Diagnosis, management, and treatment of hepatitis C: an update. Hepatology 2009;49:1335–1374. 10.1002/hep.22759 19330875 PMC7477893

[7] McHutchison JG, Manns M, Patel K, Poynard T, Lindsay KL, Trepo C, et al Adherence to combination therapy enhances sustained response in genotype-1-infected patients with chronic hepatitis C. Gastroenterology 2002;123:1061–1069. 12360468 10.1053/gast.2002.35950

[8] Reddy KR, Shiffman ML, Morgan TR, Zeuzem S, Hadziyannis S, Hamzeh FM, et al Impact of ribavirin dose reductions in hepatitis C virus genotype 1 patients completing peginterferon alfa-2a/ribavirin treatment. Clin Gastroenterol Hepatol 2007;5:124–129. 17196435 10.1016/j.cgh.2006.10.008

[9] Sievert W, Dore GJ, McCaughan GW, Yoshihara M, Crawford DH, Cheng W, et al Virological response is associated with decline in hemoglobin concentration during pegylated interferon and ribavirin therapy in hepatitis C virus genotype 1. Hepatology 2011;53:1109–1117. 10.1002/hep.24180 21480317

[10] Epstein M. Guidelines for good pharmacoepidemiology practices (GPP). Pharmacoepidemiol Drug Saf 2005;14:589–595. 15918159 10.1002/pds.1082

[11] Ferenci P, Aires R, Ancuta I, Arohnson A, Cheinquer H, Delic D, et al A tool for selecting patients with a high probability of sustained virological response to peginterferon alfa-2a (40kD)/ribavirin. Liver Int 2014;34:1550–1559. 10.1111/liv.12439 24329937

[12] Singal AK, Jampana SC, Anand BS. Peginterferon alfa-2a is superior to peginterferon alfa-2b in the treatment of naive patients with hepatitis C virus infection: meta-analysis of randomized controlled trials. Dig Dis Sci 2011;56:2221–2226. 10.1007/s10620-011-1765-0 21643737

[13] Ge D, Fellay J, Thompson AJ, Simon JS, Shianna KV, Urban TJ, et al Genetic variation in IL28B predicts hepatitis C treatment-induced viral clearance. Nature 2009;461:399–401. 10.1038/nature08309 19684573

[14] Manns MP, McHutchison JG, Gordon SC, Rustgi VK, Shiffman M, Reindollar R, et al Peginterferon alfa-2b plus ribavirin compared with interferon alfa-2b plus ribavirin for initial treatment of chronic hepatitis C: a randomised trial. Lancet 2001;358:958–965. 11583749 10.1016/s0140-6736(01)06102-5

[15] Fried MW, Shiffman ML, Reddy KR, Smith C, Marinos G, Gonçales FL Jr., et al Peginterferon alfa-2a plus ribavirin for chronic hepatitis C virus infection. N Engl J Med 2002;347:975–982. 12324553 10.1056/NEJMoa020047

[16] Hadziyannis SJ, Sette H Jr., Morgan TR, Balan V, Diago M, Marcellin P, et al Peginterferon-alpha2a and ribavirin combination therapy in chronic hepatitis C: a randomized study of treatment duration and ribavirin dose. Ann Intern Med 2004;140:346–355. 14996676 10.7326/0003-4819-140-5-200403020-00010

[17] Aronsohn A, Ancuta I, Caruntu F, Coppola C, Delic D, Digiacomo A, et al Impact of age on viral kinetics of peginterferon alfa-2a/ribavirin in chronic hepatitis C: final analysis from the PROPHESYS cohort. J Viral Hepat 2014;21:377–380. 10.1111/jvh.12179 24131506

[18] Negro F, Clement S. Impact of obesity, steatosis and insulin resistance on progression and response to therapy of hepatitis C. J Viral Hepat 2009;16:681–688. 10.1111/j.1365-2893.2009.01186.x 19732324

[19] Reddy KR, Shiffman ML, Rodriguez-Torres M, Cheinquer H, Abdurakhamov D, Bakulin I, et al Induction pegylated interferon alfa-2a and high dose ribavirin do not increase SVR in heavy patients with HCV genotype 1 and high viral loads. Gastroenterology 2010;139:1972–1983. 10.1053/j.gastro.2010.08.051 20816836

[20] McHutchison JG, Lawitz EJ, Shiffman ML, Muir AJ, Galler GW, McCone J, et al Peginterferon alfa-2b or alfa-2a with ribavirin for treatment of hepatitis C infection. N Engl J Med 2009;361:580–593. 10.1056/NEJMoa0808010 19625712

[21] Sulkowski MS, Shiffman ML, Afdhal NH, Reddy KR, McCone J, Lee WM, et al Hepatitis C virus treatment-related anemia is associated with higher sustained virologic response rate. Gastroenterology 2010;139:1602–1611. 10.1053/j.gastro.2010.07.059 20723545

[22] Lindsay KL, Morishima C, Wright EC, Dienstag JL, Shiffman ML, Everson GT, et al Blunted cytopenias and weight loss: new correlates of virologic null response to re-treatment of chronic hepatitis C. Clin Gastroenterol Hepatol 2008;6:234–241. 10.1016/j.cgh.2007.11.020 18237873

