# Supplementary material for: Impact of Safety-Related Dose Reductions or Discontinuations on Sustained Virologic Response in HCV-Infected Patients: Results from the GUARD-C Cohort
Source: PLoS One. 2016 Mar 28;11(3):e0151703. doi: 10.1371/journal.pone.0151703 (PMC4809570; doi:10.1371/journal.pone.0151703)
Supplement: S1 File — (DOCX) [file pone.0151703.s001.docx]

The following ethics committees/institutional review boards, listed by country, considered and approved the study protocol.

**Albania:** National Ethics Committee of Albania, Tirana.

**Algeria:** Comité d'Éthique Pour Les Essais Cliniques du Centre Hospitalo Universitaire de Beni Messous, Chéraga.

**Bahrain:** SMC Ethics Committee, Manama; Bahrain Defence Force, Royal Medical Services, Rifaa.

**Belgium:** Comité d'Ethique UCL - Saint-Luc, Bruxelles.

**Bosnia and Herzegovina:** Ethics Committee, University Clinical Center Tuzla, Tuzla; Ethics Committee of the Clinical Center Banja Luka, Banja Luka; Ethics Committee of the Clinical Center Mostar, Mostar; HA/Agency for Medicinal Products and Medicinal Devices of Bosnia and Herzegovina, Banja Luka; Ethics Committee of Clinical Center University Sarajevo, Sarajevo; Ethics Committee of Cantonal Hospital Zenica, Zenica.

**Brazil:** Comitê de Ética em Pesquisa Hospital Universitário Graffree e Guinle/HUGG/UNIRIO, Rio de Janeiro; CEP do Centro de Ciencas Médicas e Biológicas - PUC/SP, Sorocaba; Universidade Metropolitana de Santos – UNIMES, Santos; Comitê de Ética em Pesquisa – UNIFESP, São Paulo; Faculdade de Medicina de Botucatu/UNESP, Botucatu; Ethics Committee, Santa Casa de Misericordia do Rio de Janeiro, Rio de Janeiro; Monte Tabor - Hospital São Rafael, Comitê de Ética em Pesquisa em Seres Humanos, Salvador; Comitê de Ética em Pesquisa – UNIFESP, São Paulo; Comitê de Ética em Pesquisa em Seres, Humanos da UFTM - Universidade Federal do Triangulo Mineiro, Uberaba; Comitêe de Ética em Pesquisa em Seres Humanos do Hospital Geral de Nova lguaçu, Nova Iguacu; Comitê de Ética em Pesquisa em seres Humanos da Faculdade de Medicina de São José do Rio Preto, São José do Rio Preto; Comitê de Ética em Pesquisa da Faculdade de Medicina do ABC, Santo Andre; Comitê de Ética em Pesquisa da UFMG – COEP, Belo Horizonte; Centro de Referência e Treinamento DST/AIDS, São Paulo.

**Egypt:** Ethics Committee for Health Care Research (ECHCR), Cairo.

**Greece:** General Hospital of Rhodes Island Ethics Committee, Rhodes; University Hospital of Patra Ethics Committee, Patra; General Hospital Tzanio Ethics Committee, Piraeus; Hippokratio Hospital Ethics Committee, Athens; Elena Venizelou Hospital Ethics Committee, Athens; Geniko Kratiko Nosokomio Ni.K.A.Ias Pirea Aghios Panteleimon, Hospital Ethics Committee, Athens; University Hospital Of Alexandroupolis Ethics Committee, Alexandropoulis; Hippokratio Hospital Ethics Committee, Thessaloniki; Laiko General Hospital Ethics Committee, Athens; University Hospital Of Ioannina Ethics Committee, Ioannina; Evangelismos Hospital Ethics Committee, Athens; Konstantopouleio General Hospital of Agia Olga Ethics Committee, Athens; Euroclinic of Athens Hospital Ethics Committee, Athens; Poliklini Hospital Ethics Committee, Athens; Red Cross Hospital Ethics Committee, Athens; Anticancer Hospital Ag. Savas Hospital Ethics Committee, Athens.

**Hungary:** ETT TUKEB, Budapest.

**India**: Institutional Review Board Fortis Hospital Noida (FHIRB), Noida; Clinical Ethics Forum, Mumbai; Ethics Committee, Asian Institute of Gastroenterology, Hyderabad; PVS Memorial Hospital Ethics Committee, Kerala; Mahalasa Independent Ethics Committee, Pune; Institutional Ethics Committee (DTEC), Dayanand Medical College and Hospital, Ludhiana.

**Iran:** National Ethics Committee on Medical Scientific Researches, Tehran.

**Italy:** CE dell' Azienda Ospedaliero-Universitaria, AOU Ospedali Riuniti Umberto I, Lancisi, Salesi, Ancona; Comitato Etico, Azienda Policlinico Umberto I, Sapienza, Roma; Comitato Etico, ASL 1 Sassari, Sassari; Comitato Etico, Area Vasta Romagna/IRST, Meldola; Comitato Etico, Ospedale Sant' Anna - Area Como Lecco, Como; Comitato Etico Provinciale, di Modena, Modena; Comitato Etico per la sperimentazione dei Farmaci, AOU Pisana - CEAV Nord Ovest, Pisa; Comitato Etico Indipendente, Ospedale Fatebenefratelli e Oftalmico, Milano; Comitato Etico, AO Spedali Civili – CEP Brescia, Brescia; Comitato Etico, ASL RM H - Lazio 2, Albano Laziale; Comitato Etico per la Sperimentazione Clinica, Provincia Di Rovigo, Rovigo; Comitato Etico, Fondazione IRCCS Ca' Granda Ospedale Maggiore Policlinico – Milano Area B, Milano; Comitato Etico, ASL NA3 Sud, Brusciano; Comitato Etico AOU Policlinico P. Giaccone, Palermo; Comitato Etico AORN San Giuseppe Moscati, Avellino; Comitato Etico AO Treviglio AO Papa Giovanni XXIII, Treviglio; Comitato Etico AOU Cagliari, Cagliari; Comitato Etico, ASL TO 2 – CEI S.Luigi Gonzaga, Torino; Comitato Bioetico, ASP Ragusa - Catania 1, Ragusa; Comitato Etico Provinciale di Parma, Parma; Segreteria Scientifico-Amministrativa, CEAS Umbria, Ellera di Corciano; Comitato Etico ASL Frosinone - Lazio 2, Frosinone; Comitato Etico Indipendente, AO G. Brotzu - AOU Cagliari, Cagliari; Comitato Etico AOU Mater Domini – CER Area Centro, Catanzaro; Comitato Etico IRCCS Casa Sollievo della Sofferenza – CE Area 5, San Giovanno Rotondo; Comitato Etico per la Sperimentazione AOU Padova, Padova; Comitato Etico Azienda ASL di Latina – Lazio 2, Latina; Comitato Etico Indipendente, Policlinico S. Orsola-Malpighi, Bologna; Comitato Etico AO Ospedali Riuniti San Giovanni e Ruggi d'Aragona, Salerno; Ufficio Ricerche Cliniche, Ospedale San Raffaele – IRCCS Lombardia, Milano; Comitato Etico IRCCS INMI L. Spallanzani, Roma; Comitato Etico per la Sperimentazione Clinica Dei Farmaci, AUSL Pescara – CE Chieti Pescara, Pescara; Comitato Etico AOU Seconda Università di Napoli, Napoli; Comitato Etico IRCCS AOU.San Martino - IST – CER S.Martino, Genova; Comitato Etico AOU Maggiore della Carità – CEI Maggiore Carità, Novara; Comitato Etico AO Ospedale di Circolo Busto Arsizio – Area Varese, Busto Arsizio; Comitato Etico Università Cattolica S. Cuore – Policlinico Gemelli, Roma; Comitato Etico Ospedale Luigi Sacco – Milano Area A, Milano; Comitato Etico Interaziendale, AO Città della Salute e Della Scienza – CEI Mauriziano ASL TO1, Torino; Comitato Etico ARNAS Garibaldi – Catania 2, Catania; Comitato Etico Azienda Sanitaria Regionale del Molise, Campobasso; Comitato Etico AO Ospedali Riuniti – Area 1, Foggia; Comitato Etico AOU S.Maria della Misericordia – CERU Udine, Udine; Comitato Etico ASL NA 1, Napoli; Comitato Etico AOU Careggi – CEAVCE, Firenze; Comitato Etico AO San Paolo – Milano Area A, Milano; Comitato di Bioetica, Provincia Religiosa di S.Pietro Fatebenefratelli – Lazio 1, Roma; Comitato Etico Scientifico, AO Ospedale Niguarda Ca' Granda – Milano Area C, Milano; Comitato Etico, AO Spedali Civili – CEP Brescia, Brescia; Comitato Etico AUSL LECCE – Area 3, Lecce; Comitato Etico AOU San Luigi Gonzaga – CEI S.Luigi Gonzaga, Orbassano; Comitato Etico AO Domenico Cotugno, Napoli; Comitato Etico Indipendente, AO G. Rummo, Benevento; Comitato Etico AO SS Antonio e Biagio e Cesare Arrigo – CEI Alessandria, Alessandria; Comitato Etico ASL BR – Area 4, Brindisi; Comitato Etico AO Bianchi Melacrino Morelli – CER Area Sud, Reggio Calabria; Comitato Etico, Ospedale Valduce – Area Como Lecco, Como; Comitato Etico , AOU Policlinico G. Martino - CE MESSINA, Messina.

**Kuwait:** Joint Committee for the Protection of Human Subjects in Research, Safat.

**Lebanon:** Hotel Dieu de France Ethics Committee, Beirut; Saint Charles Hospital Ethics Committee, Baabda; Ethics Committee of Saida Governmental Hospital, Saida; Ethics Committee of Sahel General Hospital, Beirut; Ethics Committee, Haykel Hospital, Tripoli.

**FYR Macedonia:** Eticka komisija, Agencija za lekovi, Ministersvo za zdravstvo na Republika Makedonija, Skopje.

**Morocco:** Comité d'Ethique de la Recherche Biomédicale, Faculté de Médecine et de Pharmacie de Rabat, Rabat.

**Pakistan:** Ethical Review Committee, Faculty of Health Sciences, Medical College, Aga Khan University, Karachi; Ethics Committee of Isra University Hospital, Hyderabad-Sindh; IRB (Ethical Committee), Services Institute of Medical Sciences, Lahore; Foundation University Medical College, Rawalpindi; Ethical Review Committee DHQ Hospital, Faisalabad.

**Poland:** Komisja Bioetyczna, Uniwersytetu Mikolaja Kopernika w Toruniu, Bydgoszcz.

**Portugal:** Comissão de Ética, Hospital Garcia de Orta, Almada; Comissão de Ética, Centro Hospitalar de Coimbra, São Martinho do Bispo; Comissão de Ètica para a Saúde, Hospital de São João, Porto; Comissão de Ética, Hospital Joaquim Urbano, Porto; Comissão de Ética, Hospital Distrital de Santarém, Santarem; Comissão Ética, Centro Hospitalar do Baixo Alentejo, Beja; Comissão de Ética, Centro Hospitalar de Lisboa Ocidental, Lisboa; Comissão Ética, Hospital Infante D. Pedro, Aveiro; Comissão Ética, Centro Hospitalar Trás-os-Montes e Alto Douro, Vila Real; Comissão Ética, Hospital Distrital de Faro, Faro; Comissão Ética, Hospital Prof. Doutor Fernando Fonseca, Amadora; Comissão de Ética, Hospital Santa Maria, Lisboa; Comissão Ética, Hospital Curry Cabral, Lisboa;

**Qatar:** Medical Research Center, Hamad Medical Corporation, Doha.

**Romania:** Comisia Naţională de Bioetică a Medicamentului şi a Dispozitivelor Medicale, Bucharest.

**Serbia:** Clinical Center of Serbia Ethics Committee, Belgrade; Military Medical Academy Ethics Committee, Belgrade; Zvezdara-University Medical Center, Office for Human Research Protections, Belgrade; Nis Medical Center Ethics Committee, Nis; Vojvodina Medical Center Ethics Committee, Novi Sad; Kragujevac Medical Center Ethics Committee, Kragujevac.

**Slovakia:** Ethics Committee, F.D.Roosevelt Hospital, Banska Bystrica.

**South Korea:** Samsung Medical Center EC, Seoul; Severance Hospital–Yonsei University IRB, Seoul; Seoul National University Hospital IRB, Seoul; St. Vincent's Hospital IRB, Gyeonggi-do; Dong-A University Hospital IRB, Busan; Soonchunhyang University Bucheon Hospital IRB, Gyeonggi-do.

**UAE:** Sheikh Khalifa Medical City Institutional Review Board, Abu Dhabi; Medical Research Committee, Dubai Health Authority, Dubai.
