# Supplementary material for: Impact of Safety-Related Dose Reductions or Discontinuations on Sustained Virologic Response in HCV-Infected Patients: Results from the GUARD-C Cohort
Source: PLoS One. 2016 Mar 28;11(3):e0151703. doi: 10.1371/journal.pone.0151703 (PMC4809570; doi:10.1371/journal.pone.0151703)
Supplement: S2 File — (PDF) [file pone.0151703.s002.pdf]

F. HOFFMANN-LA ROCHE
OBSERVATIONAL PLAN

A Post Approval Safety Study (PASS):

Global Observational Cohort Study on the Prediction of Unwanted Adverse Effects in Individuals Infected with Chronic Hepatitis C Receiving a Long-Acting Interferon plus Ribavirin

GUARD-C

Observational Plan Approval

Observational Plan Version:	Version A
Date:	14 August 2009
International Medical Leader:	Andreas Tietz, MD
Biostatistician:	Diethelm Messinger

Confidentiality Statement

The information contained in this document, especially unpublished data, is the property of F. Hoffmann-La Roche Ltd/Inc/AG/Roche Global Business (or under its control), and therefore provided to you in confidence as an investigator, potential investigator or consultant, for review by you, your staff and an applicable Independent Ethics Committee/Institutional Review Board. It is understood that this information will not be disclosed to others without written authorization from Roche except to the extent necessary to obtain informed consent where local regulations allow or require it.

SYNOPSIS OF OBSERVATIONAL PLAN NUMBER MV22255

TITLE	Global Observational Cohort Study on the Prediction of Unwanted Adverse Effects in Individuals Infected with Chronic Hepatitis C Receiving a Long-Acting Interferon plus Ribavirin
ACRONYM	GUARD-C Study
SPONSOR	F. Hoffmann-La Roche
STUDY PHASE	Non–Interventional
INDICATION	Chronic hepatitis C (CHC)
OBJECTIVES	Primary<br>• To assess in routine clinical practice<br>➢ (a) **baseline** predictors for safety related dose reductions / treatment discontinuations and<br>➢ (b) the impact of safety related dose reductions / treatment discontinuations on sustained virological response (SVR)<br>in subjects with CHC virus infection receiving combination therapy with a long-acting interferon plus ribavirin according to local label<br>Secondary<br>• To assess in routine clinical practice **on-treatment** predictors for safety related dose reductions / treatment discontinuations<br>• To assess the association between the degree of dose reductions / treatment interruptions and SVR<br>• To evaluate and compare the predictive value of on-treatment virological response (rapid virological response [RVR], early virological response [EVR]) in naïve and treatment experienced subjects<br>• To assess the safety profile of extended treatment duration (in particular beyond 48 weeks) in subjects with CHC
TRIAL DESIGN	Prospective, international, multicenter, non-interventional cohort study in CHC subjects receiving combination therapy with a long-acting interferon plus ribavirin.<br>Dosing and treatment duration are at the discretion of the investigator in accordance with local labeling.
NUMBER OF SUBJECTS	3,000-4,000 subjects, including treatment experienced subjects (e.g. non-responder and relapser)
TARGET POPULATION	Adult (according to local legislation) subjects receiving treatment for CHC with a long-acting interferon plus ribavirin according to standard of care and in line with the current summary of product characteristics (SPC) / local labeling who have no contra-indication to long-acting interferon and ribavirin therapy as per the local label and who give consent to take part where local regulations allow or require it within up to 4 weeks after commencing treatment.

LENGTH OF STUDY	Subjects will be observed for the duration of their treatment with a long-acting interferon plus ribavirin and for up to 6 months thereafter, in line with the current summary of product characteristics (SPC) / local labeling.<br>First subject expected to be enrolled in Quarter IV, 2009.
END OF STUDY	End is expected in Quarter III, 2013 or when last subject completes observation, if earlier.
INVESTIGATIONAL MEDICAL PRODUCT(S)<br>DOSE/ ROUTE/ REGIMEN	Not applicable
NON-INVESTIGATIONAL MEDICAL PRODUCT(S)	Not applicable
DOSE/ ROUTE/ REGIMEN	According to the local label
ASSESSMENTS OF:	

Primary Variables:

- Time to first safety related dose reduction / treatment discontinuation of the long-acting interferon or ribavirin
- SVR rate defined as percentage of subjects with HCV RNA <50 IU/mL (as measured by a commercially available polymerase chain reaction [PCR] test in IU/mL) at 24 weeks post completion of the treatment period

Secondary Variables:

- Time to first safety related dose reduction / treatment discontinuation of the long-acting interferon
- Time to first safety related dose reduction / treatment discontinuation of ribavirin
- Degree of dose reductions expressed as percentage of the actual exposure in relation to the target exposure for
  a) the cumulative dose of long-acting interferon
  b) the cumulative dose of ribavirin
  c) the duration of treatment.
  d) each of the above restricted to the first 12 treatment weeks
- Percentage of missed treatment days in relation to the target number of treatment administrations for
  a) long-acting interferon
  b) ribavirin
  c) each of the above restricted to the first 12 treatment weeks
- Change in hemoglobin, neutrophil count and platelets from baseline to treatment weeks 2 and 4
- Change in body weight from baseline
- Virological response (HCV RNA <50 IU/mL) at various on-treatment time points and end of treatment
- Virological response during the first 12 weeks by the following disjunct categories
  - (a) Percentage of subjects with RVR defined as HCV RNA <50 IU/mL at study week 4

- (b) Percentage of subjects with complete early virological response (cEVR) defined as HCV RNA <50 IU/mL at study week 12, but no HCV RNA <50 IU/mL at week 4
- (c) Percentage of subjects with partial early virological response (pEVR) defined as at least a 2 log drop of HCV RNA at study week 12, but no HCV RNA <50 IU/mL at weeks 4 and 12
- (d) none of the above

- Serious and non-serious adverse event rate and profile separately for treatment naïve, treatment experienced and human immunodeficiency virus (HIV) co-infected subjects
- Serum alanine aminotransferase (ALT) and ALT ratio

- PHARMACOKINETICS/ PHARMACODYNAMICS	Not applicable
- PHARMACOECONOMICS/ QUALITY OF LIFE (QOL)	Not applicable

CLINICAL AND LABORATORY ASSESSMENTS TO BE DOCUMENTED IN THE eCRF

The following observations and assessments generally form part of routine clinical practice in management of CHC treatment. Where data are available in the medical record, these will be recorded in the electronic Case Report Form (eCRF).

- Demographics
  - age, gender, ethnic origin
  - height and body weight, the latter prior to, during and after treatment (see data collection overview)
- HCV disease characteristics
  - date and mode of infection
  - genotype
- Liver assessment
  - fibrosis assessment if available (e.g. by biopsy or liver elastography or indices calculated from blood tests); in the absence of biopsy best guess by investigator
  - in patients with cirrhosis: Child-Pugh Score
  - presence of esophageal varices
- Medical history
  - history of psychiatric symptoms / disease (e.g. depression), psychiatric medication during the preceding year and concomitant psychiatric medication
  - history of diabetes mellitus and concomitant medication
  - history of cardio-vascular disease and concomitant medication
  - history of pulmonary disease and concomitant medication
  - history of thyroid disease and concomitant medication
  - history of chronic skin disease including dermatologic extrahepatic manifestation of CHC

  - history of HIV co-infection, CD4 count, HIV RNA and concomitant anti-retroviral medication
  - history of hepatitis B virus (HBV) co-infection
  - smoking, drug use and alcohol consumption
  - pregnancy status
- Prior treatment for CHC (if applicable)
  - drug names, doses and duration as well as the virological response and reason for premature discontinuation (if applicable)
- Virology
  - quantitative HCV RNA by PCR Test in IU/mL (test name and lower limit of detection to be provided) prior to and during treatment (see data collection overview)
  - qualitative HCV RNA by PCR Test (test name and lower limit of detection in IU/mL to be provided) during and after treatment (see data collection overview)
- Clinical chemistry
  - creatinine at baseline
  - fasting insulin, glucose, high-density lipoprotein (HDL), low-density lipoprotein (LDL), cholesterol and triglycerides at baseline
  - ferritin and C-reactive protein (CRP) at baseline
  - serum ALT including information on the upper limit of normal of the respective test prior to, during and after treatment (see data collection overview)
  - thyroid stimulating hormone (TSH) prior to, during and after treatment (see data collection overview)
- Hematology
  - thrombin time at baseline
  - hemoglobin, platelets, neutrophils prior to, during and after treatment (see data collection overview)
- Immunology
  - alpha-fetoprotein at baseline
  - anti-nuclear antibodies (ANA), anti-mitochondrial antibodies (AMA), anti-smooth muscle antibodies (ASMA) and anti liver-kidney microsomal antibodies (ALKM) at baseline
- Long-acting interferon plus ribavirin therapy
  - actual doses (with brand name) received (including missed doses and interruptions) and reasons for dose changes
  - intended and actual duration of combination therapy
  - best guess of investigator regarding patient adherence
- Concomitant therapy
  - medication for concomitant diseases of special interest (see above, medical history)
  - use of growth factors (e.g. erythropoietin, granulocyte stimulating factor [GCSF]) and transfusions
  - use of methadone, other substitution therapy or iv drugs

	– prophylactic use of antibiotics (for the prevention of spontaneous bacterial peritonitis)<br>– concomitant medication for CHC (e.g. herbal drugs)<br>• Safety assessments<br>– serious and non-serious adverse events
STATISTICAL ANALYSES	Sample Size Calculation:<br><br>For the samples size calculation the following assumptions are made. The event rate of safety related dose reductions or treatment discontinuations is expected to be in the range of 20 to 40%. The standard deviation of an explanatory covariate X_1 of interest used in a Cox proportional hazard model is assumed to be in the range of 0.4 to 0.5 (possible after appropriate unit conversion). Furthermore, it is assumed that a risk reduction by 25% (equivalent to a hazard ratio of 0.75) for a 1-unit change of a covariate X_1 should be detected with 80% power. Since multiple covariates should be included into the Cox regression model an R^2 of 0.1 to 0.2 is assumed for the multiple regression of the covariate X_1 on other covariates. A sample size of 2,500 evaluable patients is required to detect a hazard ratio of 0.75 with 80% power at a significance level of 0.05 under the assumption that the standard deviation for a covariate of interest X_1 is 0.4, and R^2 of X_1 with other Xs is 0.2. In total 3,000 patients should be included to take into account patients with missing values, which can not be considered in the multivariable Cox regression model. The sample size required for other scenarios is given in Section 8.4.<br><br>Analysis Plan.<br>Cox proportional hazard models will be used to examine the predictive value of various covariates on the primary variable ‘time to the first safety related dose reduction or treatment discontinuation of the long-acting interferon or ribavirin’. The time will be calculated as days from first study treatment to the day of the first dose reduction or discontinuation due to safety reasons. If a patient discontinues the study treatment for any other reasons, then the time will be considered censored at the last treatment day.<br>Demographic data, baseline disease characteristics, other pre-study data (e.g. prior CHC treatment) and the initial interferon and ribavirin dose will be examined in the Cox proportional hazard model. For on-treatment predictors additional Cox proportion hazard models will be applied considering those variables as time-dependent covariates. Furthermore, early safety endpoints (e.g. change in neutrophil count from baseline to week 2) will be considered in a Cox regression landmark analysis together with other pre-treatment factors.<br>Multiple logistic regression (MLR) methods will be used to investigate the impact of time to safety related dose reductions or treatment discontinuation and other treatment exposure covariates on SVR, adjusted for relevant pre-treatment predictors of SVR. Since treatment discontinuations due to lack of EVR have a large impact on SVR rates,

	patients with treatment discontinuations due to insufficient virological response or other non-safety reasons will be excluded from the MLR analysis. A similar MLR analysis will investigate the impact of safety related dose reductions and treatment discontinuations within the first 12 weeks on EVR (at least a 2 log drop in HCV RNA at week 12).<br>Generalized additive models (GAM) and receiver operating characteristics (ROC) curve analysis will be used to search for appropriate cut-offs for continuous covariates which are predictive for SVR.<br>Virological response over time will be summarized for various time points and subgroups. Positive and negative predictive values of RVR and EVR on SVR will be analyzed for various subgroups.<br>Standard safety data analysis will be performed by treatment duration.

Data Collection Overview (as per standard of care)

Global Observational Cohort Study on the Prediction of Unwanted Adverse Effects in Individuals Infected with Chronic Hepatitis C Receiving a Long-Acting Interferon plus Ribavirin (GUARD-C Study)

**Assessment / Procedure** (optional, available data will be collected)	**Pre-Therapy**	**During Long-Acting Interferon plus Ribavirin Therapy (weeks)**							**Post Therapy (weeks)**
Weeks from start of therapy		**2**	**4**	**12**	**(24[3])**	**(48[4])**	**(72[5])**	**EoT[7]**	
Weeks from actual end of therapy									**24**
Informed consent (where local regulations allow or require it)[1]	×[1]								
Demographics	×								
Medical history	×								
Liver assessment (e.g. biopsy)	×								
Genotyping of HCV	×								
Prior treatment for CHC (if applicable)	×								
Selection criteria according to SPC/local labeling	×								
Evidence that female subject or female partner of male subject is not pregnant in accordance with local label[2]	×	Monthly home-based urine pregnancy tests in female subjects and female partners of male subjects in accordance with local label							
Chemistry	×								
Immunology	×								
Quantitative HCV RNA in IU/mL by PCR	×	×	×	×					
Qualitative HCV RNA by PCR		×[6]	×[6]	×[6]	×			×	×
Serum ALT	×		×	×	×	×	×	×	×
Hemoglobin, platelets, neutrophils	×	×	×	×	×	×	×	×	×
Thrombin time	×								
TSH	×			×	×	×	×	×	×
Body weight	×		×	×	×	×	×	×	×
Long-acting IFN and RBV exposure[8]		×	×	×	×	×	×	×	
Pre-defined concomitant medication[9]		×	×	×	×	×	×	×	×
Serious and non-serious Adverse Events		continuously							

1) Informed consent must be given before any data collection and may be given up to 4 weeks after treatment initiation
2) SPC wording for female subjects: "Pregnancy test immediately prior to initiation of therapy"
3) For patients receiving more than 24 weeks of therapy
4) For patients receiving more than 48 weeks of therapy
5) For patients receiving more than 72 weeks of therapy
6) Only if quantitative HCV RNA test is below level of quantification
7) EoT=End of therapy
8) As from start of therapy , i.e. start date and dose, intended treatment duration
9) As from start of therapy, documentation of ongoing medication (only psychiatric medication during preceding year)

TABLE OF CONTENTS .... Page

PART I: STUDY DESIGN AND CONDUCT 14

1. BACKGROUND AND RATIONALE 14
   - 1.1 Background 14
   - 1.2 Rationale 15
2. OBJECTIVES OF THE STUDY 16
   - 2.1 Primary Objective 16
   - 2.2 Secondary Objectives 16
3. COHORT STUDY DESIGN 16
   - 3.1 Overview of Cohort Study Design 16
   - 3.2 Number of Subjects Observed in the Cohort Study 17
   - 3.3 Centers 17
4. COHORT STUDY POPULATION 17
   - 4.1 Target Population 17
   - 4.2 Concomitant Medication and Treatment 18
5. ASSESSMENTS FOR DOCUMENTATION 19
   - 5.1 Data Collection Overview (as per standard of care) 19
   - 5.2 Pre-Therapy Data for Documentation in this Cohort Study 20
   - 5.3 Data Collected During Observation Period 21
   - 5.3.1 Laboratory Data for Documentation 21
   - 5.3.2 Documentation of Long-Acting Interferon plus Ribavirin Therapy 22
   - 5.3.3 Documentation of Pre-Defined Concomitant Medication 22
   - 5.3.4 Safety Assessments 23
6. ENDPOINTS OF THE STUDY 23
   - 6.1 Primary Variables 23
   - 6.2 Secondary Variables 23
7. SAFETY ISSUES 24
   - 7.1 Adverse Events and Laboratory Abnormalities 24
   - 7.1.1 Clinical Adverse Events 24
   - 7.1.1.1 Intensity 24
   - 7.1.1.2 Drug – Adverse Event relationship 25
   - 7.1.2 Laboratory Test Abnormalities 25
   - 7.2 Handling of Safety Parameters 26
   - 7.2.1 Serious Adverse Events (Immediately Reportable to Roche) 26

7.2.2 Treatment and Follow-up of Adverse Events 27
7.2.3 Follow-up of Abnormal Laboratory Test Values 27
7.2.4 Pregnancy 27
7.3 Premature Withdrawal 27

8. STATISTICAL CONSIDERATIONS AND ANALYTICAL PLAN 28

8.1 General 28
8.1.1 Analysis Time Windows 28
8.1.2 Types of Analyses 28
8.1.2.1 Definition of Analysis Populations 28
8.1.2.2 Exclusion of Data from Analysis 29
8.2 Primary and Secondary Study Variables 29
8.2.1 Primary Variables 29
8.2.2 Secondary Variables 30
8.3 Statistical and Analytical Methods 31
8.3.1 Analysis Plan 31
8.3.1.1 Analysis of Primary Safety Endpoint 31
8.3.1.2 Analysis of Secondary Safety Endpoint 32
8.3.1.3 Analysis of Primary Efficacy Endpoint 32
8.3.1.4 Analysis of Secondary Efficacy Endpoint 33
8.3.1.5 Subgroup Analyses 34
8.3.1.6 Standard Safety Data Analysis 35
8.3.1.7 Interim Analysis 36
8.4 Sample Size 36

9. DATA COLLECTION, MANAGEMENT AND QUALITY ASSURANCE 38

10. REFERENCES 39

PART II: ETHICS AND GENERAL STUDY ADMINISTRATION 44

11. ETHICAL ASPECTS 44

11.1 Guidelines for Epidemiological Studies 44
11.2 Informed Consent 44
11.3 Independent Ethics Committees/Institutional Review Board 44

12. CONDITIONS FOR MODIFYING THE OBSERVATIONAL PLAN 45

13. CONDITIONS FOR TERMINATING THE STUDY 45

14. STUDY DOCUMENTATION, eCRFS AND RECORD KEEPING 45

14.1 Investigator's Files / Retention of Documents 45
14.2 Source Documents and Background Data 46

14.3 Audits and Inspections 46
14.4 Electronic Case Report Forms........ 46
14.5 Contract Research Organizations 46

15. MONITORING THE STUDY 47

16. CONFIDENTIALITY OF TRIAL DOCUMENTS AND SUBJECT RECORDS........ 47

17. PUBLICATION OF DATA AND PROTECTION OF TRADE SECRETS........ 47

LIST OF TABLES Page

Table 1 Data collected from the pre-therapy period........ 20
Table 2 Laboratory assessments collected during observation period 22
Table 3 Analysis Time Windows 28
Table 4 Cross-tabulation of Early Virological Response and SVR 34
Table 5 Sample Size for Cox Proportional Hazard Analysis 37

LIST OF APPENDICES Page

Appendix 1 Child-Pugh Classification of Severity of Liver Disease 43

GLOSSARY OF ABBREVIATIONS

Ab	Antibody
AE	Adverse event
Ag	Antigen
ALKM	Anti liver-kidney microsomal antibodies
ALT (SGPT)	Alanine aminotransferase
AMA	Anti-mitochondrial antibodies
ANA	Anti-nuclear antibodies
ANOVA	Analysis of variance
AP	Alkaline phosphatase
ASMA	Anti-smooth muscle antibodies
AST (SGOT)	Aspartate aminotransferase
AZT	Azidothymidine
BP	Blood pressure
cEVR	Complete early virological response
CHB	Chronic hepatitis B
CHC	Chronic hepatitis C
CI	Confidence interval
CRA	Clinical Research Associate
CRP	C-reactive protein
CXR	Chest x-ray
d4T	Stavudine
ddl	Didanosine
eCRF	Electronic Case Report Form
EDC	Electronic Data Capture
EMEA	European Agency for the Evaluation of Medicinal Products
EVR	Early virological response
GAM	Generalized additive models
GPP	Good Pharmacoepidemiology Practices
GCP	Good Clinical Practice
GCSF	Granulocyte stimulating factor
HAART	Highly active antiretroviral therapy
HBV	Hepatitis B virus
HCC	Hepatocellular carcinoma
HCV	Hepatitis C virus
HDL	High-density lipoprotein
HIV	Human immunodeficiency virus
ICH	International Conference on Harmonization
IEC	Independent Ethics Committee

GLOSSARY OF ABBREVIATIONS (cont'd)

IFN	Interferon alpha
IRB	Institutional Review Board
IgM	Immunoglobulin M antibody
ITT	Intent-to-treat
iv	Intravenous
LDL	Low-density lipoprotein
LFT	Liver function test
µg	Microgram
MIU	Million International Units
mL	Milliliter
MLR	Multiple logistic regression
mRNA	Messenger ribonucleic acid
ng	Nanogram
PASS	Post Approval Safety Study
PCR	Polymerase chain reaction
PD	Pharmacodynamic
PE	Pharmacoeconomic
pEVR	Partial early virological response
PK	Pharmacokinetic
po	per os
PR	Pulse rate
QoL	Quality of life
RNA	Ribonucleic acid
ROC	Receiver operating characteristics
RVR	Rapid virological response
sAE	Serious adverse event
sc	Subcutaneous
SI	Système International
SPC	Summary Of Product Characteristics
SSRI	Selective Serotonin Reuptake Inhibitors
SVR	Sustained virological response
t.b.d.	To be determined
tiw	Three times per week
TSH	Thyroid stimulating hormone
ULN	Upper limit of normal
WBC	White blood count

PART I: STUDY DESIGN AND CONDUCT

1. BACKGROUND AND RATIONALE

1.1 Background

Chronic infection with hepatitis C virus (HCV) is a global health problem affecting 170–200 million people worldwide, including up to 4 million people in the United States and approximately 8–9 million in Europe [1, 2, 3]. The natural course of the disease leads to progressive fibrosis and eventually cirrhosis of the liver in up to 20% of infected individuals, and hepatocellular carcinoma in up to 2.5% [4]. Patients with cirrhosis have a high risk of progression to hepatic failure and HCV infection is the most common indication for liver transplantation [5, 6]. The global prevalence of Chronic Hepatitis C (CHC) is estimated to be 3% ranging from 0.1–1% in Western Europe and up to 4.9% in some regions of Eastern Europe [3]. Although the incidence of CHC continues to decrease, the overall prevalence is predicted to remain relatively constant for several years to come [7]. In persons 30–49 years of age, the prevalence of the disease is 3–fold higher than in other age groups, therefore the morbidity and mortality associated with CHC is expected to increase substantially between 2009 and 2019 [8].

The elimination of the virus, defined as the absence of HCV RNA in serum by a sensitive test at the end of treatment and 6 months later (sustained virological response [SVR]), is the therapeutic goal of CHC treatment [9, 10, 11] and is generally regarded as a cure [10, 11].

Interferon alpha (IFN) was the first drug shown to have activity against HCV. However, the effectiveness of IFN monotherapy in the treatment of CHC infection was generally unsatisfactory resulting in an overall SVR rate of <20% [12, 13]. Combination therapy with IFN plus ribavirin improved SVR rates but overall rates remained below 50% [14, 15, 16]. However, following the introduction of long-acting interferons (e.g. peginterferon alfa-2a and alfa 2b) in combination with ribavirin further advances have been made in efficacy with rates of SVR now up to 66% with this treatment regimen [14, 15, 17].

These achievements are reflected in current treatment guidelines issued by the liver societies of the USA (American Association for the Study of Liver Diseases) [9, 11, 18], Asia (Asian Pacific Association for the Study of the Liver) [19] and in Europe [20] with combination therapy with pegylated interferon alfa plus ribavirin now considered standard of care for CHC.

The recommended treatment duration among patients undergoing a first course of treatment with peginterferon alfa plus ribavirin for those infected with HCV genotype 2/3 is 24 weeks and 48 weeks for patients infected with HCV genotype 1/4 [21]. However, recent data have shown that patients with a low viral load at baseline and a rapid viral response defined as undetectable HCV at week 4 of treatment, may benefit from an abbreviated duration of treatment of 16 weeks (genotype 2/3) or 24 weeks (genotypes 1/4) [22, 23, 24, 25, 26, 27]. Furthermore recently the EMEA (European Agency for the Evaluation of Medicinal Products) approved a 72 week treatment duration with peginterferon alfa-2a plus ribavirin for treatment experienced patients infected with HCV genotype 1 [22, 28].

Pivotal trials with long-acting interferons in combination with ribavirin have demonstrated that as many as 30% of patients initiating treatment discontinue treatment prior to completing scheduled treatment for a variety of reasons including adverse events [14, 15, 21]. An even higher proportion of patients require dose reductions, primarily to manage adverse events and laboratory abnormalities [14, 15, 21].

Not all patients with diagnosed infection with CHC undergo treatment with a long-acting interferon in combination with ribavirin. Possible reasons for treatment barriers include the presence of co-morbidities including psychiatric symptoms, active substance use, socio-economic reasons and patient preference to delay treatment which may reflect concerns related to the expected side effect profile [29, 30, 31, 32].

Physicians may be reluctant to initiate treatment in patients with a perceived higher risk of developing side effects (requiring dose reductions or treatment discontinuation), i.e. elderly patients, patients with cirrhosis, a history of psychiatric disease or insulin resistance or subjects actively using drugs. However, data on treatment response from these patient groups show that many can be successfully treated once treatment adherence of >80% can be achieved e.g. by early management of side effects or – particularly in patients actively using drugs or on methadone programs – directly observed administration of the medicine [33, 34, 35, 36, 37, 38, 39, 40].

Early on-treatment response defined as an undetectable HCV RNA at week 4 or 12 is a further instrument to detect patients with high chances of achieving an SVR and may encourage them to finalize the prescribed treatment course.

1.2 Rationale

Data available from controlled clinical trials in CHC demonstrate that adherence to therapy is an important factor for achieving SVR [41, 42, 43]. On the other hand the known side effect profile of interferon based therapy often requires dose reductions or even interruptions, e.g. due to hematological abnormalities such as neutropenia or anemia [44]. Recently it has been shown that awareness of the side effect profile and early interventions can avoid dose reductions, e.g. administration of Selective Serotonin Reuptake Inhibitors (SSRI) for depression [36, 37], use of erythropoietin to control anemia [45], availability of medical personnel to counsel patients, provide personal advice or directly observe therapy. A recent review shows the lack of data on patient adherence in the field of HCV therapy [46].

The rationale of the present study is to collect information from real world daily clinical practice on dose reductions/interruptions/missed doses due to side effects and to correlate such dose modifications with treatment outcome. Although it may seem likely that such dose reductions will have an impact on SVR, recent publications have shown that patients suffering from anemia, neutropenia or weight loss during therapy (and eventually requiring dose reductions) had higher chances to achieve an SVR than patients without such adverse drug reactions [45, 46].

A further rationale is to identify baseline or on-treatment predictors for dose reductions due to side effects thus assisting physicians in the future to make treatment decisions for individual patients. The data base might also provide valuable insight on how side effects are managed in clinical practice.

Finally this study intends to document (in an international non-interventional cohort) the adverse event profile of prolonged treatment duration of up to 72 weeks in treatment experienced patients. This is a commitment to the EMEA on approval of the new indication for peginterferon alfa-2a [22].

2. OBJECTIVES OF THE STUDY

2.1 Primary Objective

To assess in routine clinical practice

(a) baseline predictors for safety related dose reductions / treatment discontinuations

and

(b) the impact of safety related dose reductions / treatment discontinuations on SVR

in subjects with CHC receiving combination therapy with a long-acting interferon plus ribavirin according to local label.

2.2 Secondary Objectives

- To assess in routine clinical practice on-treatment predictors for safety related dose reductions / treatment discontinuations
- To assess the association between the degree of dose reductions / treatment interruptions and SVR
- To evaluate and compare the predictive value of on-treatment virological response (rapid virological response [RVR], early virological response [EVR]) in naïve and treatment experienced subjects
- To assess the safety profile of extended treatment duration (in particular beyond 48 weeks) in subjects with CHC

3. COHORT STUDY DESIGN

3.1 Overview of Cohort Study Design

This is a prospective, international, multicenter, observational, non-interventional cohort study in subjects with CHC receiving therapy with a long-acting interferon plus ribavirin.

A cohort of adult subjects who are receiving therapy with a long-acting interferon plus ribavirin for CHC according to standard of care and in line with local labeling will be followed for the duration of their treatment with a long-acting interferon plus ribavirin and for up to 24 weeks after therapy was terminated.

Dosing and treatment duration of the long-acting interferon plus ribavirin are at the discretion of the investigator in accordance with local clinical practice and local labeling.

During treatment, laboratory and clinical assessments are routinely performed in accordance with current guidelines and local standard of care. During this cohort study, available data from the medical record relevant for safety and treatment outcome will be documented in the electronic Case Report Form (eCRF) during therapy with a long-acting interferon plus ribavirin and for up to 24 weeks after treatment was terminated (please refer to Data Collection Overview, Section 5.1).

The end of study will be the date of the last visit of the last subject undergoing the study. Last subject, last visit is **either** the date of the last subject visit to complete the study, **or** the date at which the last data point from the last subject, which is required for statistical analysis (i.e. key safety and efficacy results for decision making) was received, whichever is the later date.

3.2 Number of Subjects Observed in the Cohort Study

Subjects qualifying for observation in the cohort will be assigned to individual study subject numbers in the order of enrollment. Each participating center will be identified by a unique center number assigned by Roche or its designee. In order to obtain data from 2,500 subjects eligible for the Cox regression model for the primary endpoint (see Section 8.4), ~3,000 subjects will be observed. Should fewer subjects than expected with sufficient data for planned analysis be available from routine clinical practice, up to 4,000 subjects might be observed.

For each new subject, the investigator or designee will create a new subject file on the web-based eCRF and a subject number will automatically be allocated by the system.

3.3 Centers

This study will be conducted at ~400 centers in approximately 15 countries.
Further countries and centers may be added or substituted if underperforming.

4. COHORT STUDY POPULATION

4.1 Target Population

Adult subjects receiving treatment for CHC with a long-acting interferon plus ribavirin according to standard of care and in line with the current summary of product characteristics (SPC) / local labeling and who have no contra-indication to long-acting interferon and ribavirin therapy as per the local label are eligible for observation in this cohort if the following applies:

- Adult (according to local legislation) male or female subjects CHC receiving combination therapy with a long-acting interferon plus ribavirin
- Quantifiable serum HCV RNA by polymerase-chain reaction (PCR) before initiation of treatment
- Subjects with no contra-indications to long-acting interferon and ribavirin therapy as detailed in the label
  [e.g.: pregnancy; breast-feeding; hypersensitivity to the active substance, to alpha interferons, ribavirin, or to any of the excipients; autoimmune hepatitis; severe hepatic dysfunction or

decompensated cirrhosis of the liver; a history of severe pre-existing cardiac disease, including unstable or uncontrolled cardiac disease in the previous six months; hemoglobinopathies (e.g. thalassemia, sickle-cell anemia); initiation of therapy with long-acting interferon plus ribavirin is contraindicated in HIV/HCV patients with cirrhosis and a Child-Pugh score ≥6 (appendix 1)]

- Subjects without end stage renal disease
- Subjects without major organ transplantation
- Subjects should not receive concomitant therapy with telbivudine (because concomitant peginterferon therapy is contraindicated according to the telbivudine label)
- Subjects with HIV/HCV co-infection should not receive concomitant therapy with azidothymidine (AZT=Zidovudin), didanosine (ddl) and stavudine (d4T) (for safety reasons)
- Female subjects not pregnant (pregnancy test immediately prior to initiation of combination therapy) or breast-feeding when combination treatment commenced
- Male partners of women who are pregnant should not receive ribavirin
- All fertile males and females receiving ribavirin must be using two forms of effective contraception during treatment and during the 6 months after treatment end
- Written informed consent where local regulations allow or require it within up to 4 weeks after commencing treatment

4.2 Concomitant Medication and Treatment

Concomitant medication for pre-defined concomitant diseases (please refer to Section 5.3.3) administered from first day of treatment with long-acting interferon/ribavirin and during long-acting interferon/ribavirin treatment will be documented in the eCRF together with the reason for prescription (i.e. disease being treated).

Should a subject receive concomitant medication for liver disease (e.g. herbal drugs) during the observation period this should be documented with type of drug and duration.

5. ASSESSMENTS FOR DOCUMENTATION

5.1 Data Collection Overview (as per standard of care)

Global Observational Cohort Study on the Prediction of Unwanted Adverse Effects in Individuals Infected with Chronic Hepatitis C Receiving a Long-Acting Interferon plus Ribavirin (GUARD-C Study)

**Assessment / Procedure** (optional, available data will be collected)	**Pre-The-rapy**	**During Long-Acting Interferon plus Ribavirin Therapy (weeks)**							**Post Therapy (weeks)**
Weeks from start of therapy		**2**	**4**	**12**	**(24[3])**	**(48[4])**	**(72[5])**	**EoT[7]**	
Weeks from actual end of therapy									**24**
Informed consent (where local regulations allow or require it)[1]	×[1]								
Demographics	×								
Medical history	×								
Liver assessment (e.g. biopsy)	×								
Genotyping of HCV	×								
Prior treatment for CHC (if applicable)	×								
Selection criteria according to SPC/local labeling	×								
Evidence that female subject or female partner of male subject is not pregnant in accordance with local label[2]	×	Monthly home-based urine pregnancy tests in female subjects and female partners of male subjects in accordance with local label							
Chemistry	×								
Immunology	×								
Quantitative HCV RNA in IU/mL by PCR	×	×	×	×					
Qualitative HCV RNA by PCR		×[6]	×[6]	×[6]	×			×	×
Serum ALT	×		×	×	×	×	×	×	×
Hemoglobin, platelets, neutrophils	×	×	×	×	×	×	×	×	×
Thrombin time	×								
TSH	×			×	×	×	×	×	×
Body weight	×		×	×	×	×	×	×	×
Long-acting IFN and RBV exposure[8]		×	×	×	×	×	×	×	
Pre-defined concomitant medication[9]		×	×	×	×	×	×	×	×
Serious and non-serious Adverse Events		continuously							

[1] Informed consent must be given before any data collection and may be given up to 4 weeks after treatment initiation
[2] SPC wording for female subjects: "Pregnancy test immediately prior to initiation of therapy"
[3] For patients receiving more than 24 weeks of therapy
[4] For patients receiving more than 48 weeks of therapy
[5] For patients receiving more than 72 weeks of therapy
[6] Only if quantitative HCV RNA test is below level of quantification
[7] EoT=End of therapy
[8] As from start of therapy, i.e. start date and dose, intended treatment duration
[9] As from start of therapy, documentation of ongoing medication (only psychiatric medication during preceding year)

5.2 Pre-Therapy Data for Documentation in this Cohort Study

The following data collection chart (Table 1) represents assessments that are routinely performed as part of current standard of care in the majority of centers prior to the initiation of therapy with long-acting interferons plus ribavirin. Data available from information documented in the subject's medical records will be entered into the eCRF. Data may be documented retrospectively up to 4 weeks after treatment initiation.

Table 1 Data collected from the pre-therapy period

Demographics	Age (date of birth), gender, ethnic origin, height and body weight
HCV disease characteristics	Probable mode and assumed year of HCV infection (injection drug use, sexual, occupational, transfusion, perinatal, unknown, other)
Medical History	Psychiatric symptoms / disease (e.g. depression), psychiatric medication during the preceding year<br>Diabetes mellitus, cardio-vascular disease, pulmonary disease, thyroid disease<br>Chronic skin disease including dermatologic extrahepatic manifestation of CHC<br>Hepatitis B virus (HBV) co-infection<br>Human immunodeficiency virus (HIV) co-infection<br>Alcohol consumption (regular alcohol consumption, average number of units/drinks per week)<br>Smoking (never, previous, if current smoker average number of cigarettes, cigars or pipes per day)<br>Drug use (never, previous, current)<br>Pregnancy status
Liver assessment (if available):	Results from liver biopsy and documentation of the histopathological scoring system used (e.g. METAVIR, Ishak, Knodell, Scheuer)<br>From non-invasive methods:<br>- liver elastography (specify kPa assessed by FibroScan)<br>- indices calculated from blood tests<br>Categorization of patients as cirrhotic (including transition to cirrhosis) or non-cirrhotic, based on all information available. In the absence of biopsy best guess by investigator<br>Presence of esophageal varices (yes/no/unknown)<br>In subjects with cirrhosis: Child-Pugh Score
Prior medication for HCV (if applicable)	Prior treatment for CHC (e.g. standard interferon, pegylated interferon, ribavirin) with drug names, start of therapy, dosage regimen (start and end

	dose), cessation of therapy, as well as the virological response (relapse, breakthrough, non-response) and reason for premature discontinuation (lack of efficacy, safety reasons, other), if applicable
Virology	HCV genotype<br>Quantitative HCV RNA by PCR Test in IU/mL (test name and lower limit of detection to be provided)<br>In subjects with HIV/HCV co-infection: HIV RNA
Immunology	Anti-nuclear antibodies (ANA), anti-mitochondrial antibodies (AMA), anti-smooth muscle antibodies (ASMA) and anti liver-kidney microsomal antibodies (ALKM)<br>Alpha-fetoprotein<br>In subjects with HIV/HCV co-infection: CD4 count
Hematology	Hemoglobin, platelets, neutrophils<br>Thrombin time
Clinical Chemistry	Alanine aminotransferase (ALT) including information on the upper limit of normal of the respective test<br>Creatinine<br>Thyroid stimulating hormone (TSH)<br>Fasting insulin, glucose, high-density lipoprotein (HDL), low-density lipoprotein (LDL), cholesterol and triglycerides<br>Ferritin and C-reactive protein (CRP)

5.3 Data Collected During Observation Period

5.3.1 Laboratory Data for Documentation

During therapy with long-acting interferons plus ribavirin, laboratory assessments are routinely performed in accordance with current guidelines and local standard of care. When performed during the observational period, available results from the range of assessments described below (Table 2) will be documented in the eCRF (please refer to Data Collection Overview, Section 5.1). Most data will be documented around weeks 2, 4, 12, 24, 48 and 72 of therapy (depending on prescribed duration of treatment regimen), at end of treatment (e.g. week 24, 48 or 72), and 24 weeks after actual treatment termination.

Table 2 Laboratory assessments collected during observation period

Virology	Quantitative HCV RNA by PCR Test in IU/mL (along with test name and lower limit of detection) at weeks 2, 4 and 12<br>(Should results on quantitative HCV RNA from further time points be available [e.g. week 8] they should be documented in the eCRF)<br>Qualitative HCV RNA by PCR Test (along with test name and lower limit of detection in IU/mL) at week 24, at end of treatment and 24 weeks after treatment discontinuation. In addition qualitative test results should be documented at weeks 2, 4 and 12 if quantitative HCV RNA test is below level of quantification at these time points.
Hematology	Hemoglobin, platelets, neutrophils at each visit time window.
Clinical Chemistry	Serum ALT including information on the upper limit of normal of the respective test at each visit time window (apart from treatment week 2).<br>TSH at weeks 12, 24, and every 6 months thereafter during therapy and at end of follow-up.

5.3.2 Documentation of Long-Acting Interferon plus Ribavirin Therapy

The starting dose, dose adjustments, missed doses, dosing interruptions, as well as the intended and actual treatment duration for the long-acting interferon and for ribavirin will be documented in the eCRF including reasons for dose changes and duration of therapy that is shorter or longer than originally planned.

The overall adherence will be calculated for each subject. In addition the investigator will be asked to document his/her best guess regarding patient adherence.

The brand names for the long-acting interferon and for ribavirin will be documented.

5.3.3 Documentation of Pre-Defined Concomitant Medication

Pre-defined concomitant medication prescribed for concomitant diseases of special interest, treatment for anemia and opioid substitution will be documented in the eCRF from start of therapy with long-acting interferon plus ribavirin:

- Concomitant medication for CHC (e.g. herbal drugs) with start and end date
- Psychiatric medication
- Anti-diabetics
- Cardio-vascular drugs

- Pulmonary drugs
- Medication for thyroid disorders
- Prophylactic use of antibiotics (for the prevention of spontaneous bacterial peritonitis)
- Methadone or other substitution therapy and use of iv drugs
- Concomitant use of growth factors (e.g. erythropoietin, granulocyte stimulating factor [GCSF]) and transfusions
- For subjects with HIV/HCV co-infection: Documentation of type and duration of HAART (highly active anti-retroviral therapy)

5.3.4 Safety Assessments

- Body weight at each visit time window (apart from week 2).
- Clinical adverse events (AEs), serious and non-serious, will be recorded in the eCRF during the total observation period of up to 2 years, with investigator assessment of intensity (mild, moderate, severe) and relationship to therapy (i.e., related or unrelated)
  (Please refer to section 7)

6. ENDPOINTS OF THE STUDY

6.1 Primary Variables

- Time to first safety related dose reduction / treatment discontinuation of the long-acting interferon or ribavirin
- SVR rate defined as percentage of subjects with HCV RNA <50 IU/mL (as measured by a commercially available PCR test in IU/mL) at 24 weeks post completion of the treatment period

6.2 Secondary Variables

- Time to first safety related dose reduction / treatment discontinuation of the long-acting interferon
- Time to first safety related dose reductions or treatment discontinuations of ribavirin
- Degree of dose reductions expressed as percentage of the actual exposure in relation to the target exposure for
  a) the cumulative dose of long-acting interferon
  b) the cumulative dose of ribavirin
  c) the duration of treatment.
  d) each of the above restricted to the first 12 treatment weeks
- Percentage of missed treatment days in relation to the target number of treatment administrations for
  a) long-acting interferon
  b) ribavirin
  c) each of the above restricted to the first 12 treatment weeks

- Change in hemoglobin, neutrophil count and platelets from baseline to treatment weeks 2 and 4
- Change in body weight from baseline
- Virological response (HCV RNA <50 IU/mL) at various on-treatment time points and end of treatment.
- Virological response during the first 12 weeks by the following disjunct categories
  - (a) Percentage of subjects with RVR defined as HCV RNA <50 IU/mL at study week 4
  - (b) Percentage of subjects with complete early virological response (cEVR) defined as HCV RNA <50 IU/mL at study week 12, but no HCV RNA <50 IU/mL at week 4
  - (c) Percentage of subjects with partial early virological response (pEVR) defined as at least a 2 log drop of HCV RNA at study week 12, but no HCV RNA <50 IU/mL at weeks 4 and 12
  - (d) none of the above
- Serious and non-serious adverse event rate and profile separately for treatment naïve, treatment experienced and HIV co-infected subjects
- Serum ALT and ALT ratio

7. SAFETY ISSUES

7.1 Adverse Events and Laboratory Abnormalities

7.1.1 Clinical Adverse Events

According to the International Conference of Harmonization (ICH), an Adverse Event (AE) is any untoward medical occurrence in a patient or clinical investigation subject administered a pharmaceutical product and which does not necessarily have to have a causal relationship with this treatment. An AE can therefore be any unfavorable and unintended sign, symptom, or disease temporally associated with the use of a medicinal product, whether or not considered related to the medicinal product. Pre-existing conditions which worsen during a study are to be reported as AE.

Clinical adverse events (AEs) encountered during the observational study will be reported on the AE pages of the eCRF. Intensity of adverse events will be graded on a three-point scale (mild, moderate, severe) and reported in detail as indicated on the eCRF. Relationship of the adverse event to the treatment should also be assessed.

7.1.1.1 Intensity

Mild:	discomfort noticed but no disruption of normal daily activity
Moderate:	discomfort sufficient to reduce or affect normal daily activity
Severe:	incapacitating with inability to work or perform normal daily activity

7.1.1.2 Drug – Adverse Event relationship

The causality relationship of long-acting interferon and/or ribavirin to the AE will be assessed by the Investigator as either:
Yes (related) or No (not related).

If there is a reasonable suspected causal relationship to the long-acting interferon and/or ribavirin, i.e. there are facts (evidence) or arguments to suggest a causal relationship, drug-event relationship should be assessed as Yes.

The following criteria should be considered in order to assess the relationship as Yes:

- Reasonable temporal association with drug administration
- It may or may not have been produced by the patient’s clinical state, environmental or toxic factors, or other modes of therapy administered to the patient.
- Known response pattern to suspected drug
- Disappears or decreases on cessation or reduction in dose
- Reappears on rechallenge

The following criteria should be considered in order to assess the relationship as No:

- It is clearly due to extraneous causes
- It does not follow a reasonable temporal sequence from administration of the drug.
- It may readily have been produced by the patient’s clinical state, environmental or toxic factors, or other modes of therapy administered to the patient.
- It does not reappear or worsen when the drug is readministered.

7.1.2 Laboratory Test Abnormalities

Laboratory test results will be recorded on the laboratory results form of the eCRF.

Study monitors will review eCRF entries on an ongoing basis. Unexplained abnormal laboratory test values should be repeated immediately if medically relevant and followed until either they have returned to the normal range and/or are adequately explained.

Any laboratory result abnormality fulfilling the criteria for a serious adverse event (SAE) should be immediately reported on an SAE reporting form, in addition to being recorded as an AE in the eCRF (see also Section 7.2.1 on SAE reporting).

Laboratory test value abnormalities should not be reported as adverse events on the "Adverse Event" electronic form of the eCRF unless the abnormal laboratory assessment meets any one of the criteria listed below:

- is considered to be an SAE
- results in discontinuation from study treatment
- results in a requirement for a change in concomitant therapy (e.g. addition of, interruption of, discontinuation of, or any other change in a concomitant medication, therapy or treatment)

7.2 Handling of Safety Parameters

7.2.1 Serious Adverse Events (Immediately Reportable to Roche)

ANY CLINICAL ADVERSE EVENT OR ABNORMAL LABORATORY TEST VALUE THAT IS **SERIOUS** (INCLUDING DEATH, OVERDOSE OR CONGENITAL ANOMALY) OCCURRING DURING THE COURSE OF THE STUDY, IRRESPECTIVE OF THE TREATMENT RECEIVED BY THE PATIENT, MUST BE REPORTED TO ROCHE (or designee) ON AN SAE REPORTING FORM WITHIN **ONE** WORKING DAY OF PARTICIPATING PHYSICIAN BECOMING AWARE OF EVENT,

FAX NO: +49 621 879 86 36

The definition and reporting requirements of the ICH Guideline for Clinical Safety Data Management, Definitions and Standards for Expedited Reporting, Topic E2 will be adhered to.

A serious adverse event is any experience that suggests a significant hazard, contraindication, side effect or precaution. With respect to human clinical experience, this includes any experience which

- is fatal (results in death; NOTE: death is an outcome, not an event);
- is life-threatening (NOTE: the term "life-threatening" refers to an event in which the patient was at immediate risk of death at the time of the event; it does not refer to an event which could hypothetically have caused a death had it been more severe);
- requires inpatient hospitalization or prolongation of an existing hospitalization;
- results in persistent or significant disability/ incapacity;
- is a congenital anomaly/ birth defect;
- is medically significant or requires intervention to prevent one or other of the outcomes listed above.

Medical and scientific judgment should be exercised in deciding whether expedited reporting is appropriate in other situations, such as important medical events that may not be immediately life-threatening or result in death or hospitalization but may jeopardize the patient or may require intervention to prevent one of the outcomes listed in the definitions above. These situations should also usually be considered serious.

The term **severe** is a measure of **intensity**, thus a severe adverse event is not necessarily serious. For example, nausea of several hours' duration may be rated as severe, but may not be clinically serious. A **death** occurring during the study, including the observational plan-defined follow-up, which comes to the attention of the investigator, whether considered treatment-related or not, must be reported.

Such preliminary reports will be followed by detailed descriptions later which will include copies of hospital case reports, autopsy reports and other documents when requested and applicable.

Related serious adverse events must be collected and reported regardless of the time elapsed from the last study drug administration, even if the study has been closed.

For serious and all other AEs, the following must be assessed and recorded on the AE page of the eCRF: intensity, relationship to long-acting interferon and/or ribavirin, action taken regarding long-acting interferon and/or ribavirin, and outcome to date.

The investigator must notify the Institutional Review Board (IRB) [Independent Ethics Committee (IEC)] of a serious adverse event in writing as soon as it is practical and in accordance with international and local laws and regulations.

Note: The definitions for and procedures for reporting SAEs to Health Authorities will be taken from the ICH guidelines. The definition of "expectedness" will include those events encountered for long-acting interferon and ribavirin in the respective SPCs / local labeling.

7.2.2 Treatment and Follow-up of Adverse Events

Patients experiencing AEs should be treated by accepted clinical procedures. All AEs should be followed until resolved or stabilized.

7.2.3 Follow-up of Abnormal Laboratory Test Values

In the event of unexplained abnormal laboratory test values, the tests should be repeated immediately if medically relevant and followed up until they have returned to the normal range and/or an adequate explanation of the abnormality is found. If a clear explanation is established it should be recorded on the eCRF.

7.2.4 Pregnancy

According to label pregnancy is to be strictly avoided during treatment with the combination of a long-acting interferon plus ribavirin. However, if a female subject becomes pregnant during the study she must be instructed to stop taking the trial medication and immediately inform the investigator. Pregnancies occurring up to 24 weeks after discontinuation of therapy must also be reported to the investigator. The investigator should report all pregnancies within 24 hours to Roche using the Study Pregnancy Reporting form.

The investigator should counsel the patient, discuss the risks of continuing with the pregnancy and the possible effects on the fetus. Monitoring of the patient should continue until conclusion of the pregnancy. Information on the outcome of the pregnancy must be provided to the sponsor.

Pregnancy occurring in the partner of a male patient participating in the study should also be reported to the investigator immediately. If the partner consents, this information will also be reported to Roche and the partner should be counseled and followed as described above. Such male patients may not continue to receive ribavirin.

7.3 Premature Withdrawal

Subjects have the right to withdraw from the cohort study at any time for any reason. The investigator also has the right to withdraw subjects from the cohort study if it is in the best interest of the subject.

8. STATISTICAL CONSIDERATIONS AND ANALYTICAL PLAN

8.1 General

8.1.1 Analysis Time Windows

Due to the non-interventional design of the study, the time points for laboratory measurements are not mandatory. However, the most recent available measurements prior to start of treatment, and all available measurements after start of treatment and up to 24 weeks post-treatment (for details see Section 5) should be entered into the eCRF. These measurements will be assigned to the following time windows (Table 3):

Table 3 Analysis Time Windows

Phase	Time point	Time Window
Pre-Therapy	Baseline	Last value prior to start of study treatment (i.e. ≤study day 1)
Treatment	Weeks from start of treatment	Study day during treatment period (Study day 1 = first treatment day)
	2	12 - 18
	4	26 - 32
	12	72 - 99
	24	156 - 183
	48	324 - 351
End of treatment (EoT virological response)	Actual end of treatment	Study day of last dose ± 28 days
Post-Treatment (SVR)	24 weeks after actual end of treatment	≥140 days after last dose of treatment

If for other time points (e.g. 12 weeks post-treatment) a relevant number of data will be available then further time windows could be defined in the statistical analysis plan.

8.1.2 Types of Analyses

8.1.2.1 Definition of Analysis Populations

Intent-to-Treat (ITT) analysis population is defined to include all subjects who received at least one dose of a long-acting interferon plus ribavirin. The primary and all other secondary efficacy parameters will be analyzed using the ITT population.

Standard analysis population will exclude a treated subject if the subject meets any of the criteria listed in Section 8.1.2.2. The main focus of this population is to perform statistical analyses in subjects with sufficiently complete data (no early dropout, no missing key information) and who will fulfill those inclusion criteria, which can be considered essential for conclusions regarding efficacy. The primary and all other secondary efficacy parameters will be analyzed using the standard population.

Safety analysis population is defined to include only subjects who received at least one dose of a long-acting interferon plus ribavirin and have at least one post-baseline safety assessment.

8.1.2.2 Exclusion of Data from Analysis

The following subjects will be excluded from the standard analysis:

1. HCV RNA <600 IU/mL (by PCR) at last measurement prior to commencing therapy
2. Subjects with missing baseline data (age, gender, weight, height, genotype, quantitative HCV RNA, information whether treatment naïve or treatment experienced)
3. Patients with no post-baseline HCV RNA assessment
4. Post-treatment HCV RNA test for SVR with a detection limit >50 IU/mL
5. Subjects who are co-infected with HBV
6. Subjects with a labeled contra-indication to therapy with long-acting interferon or ribavirin
7. Starting doses and intended treatment durations, which are markedly lower than recommended by the package inserts (details will be specified in the Statistical Analysis Plan – SAP)
8. Insufficient treatment information (details will be specified in the SAP).

The following patients will be excluded from the safety analysis:

1. Patients who never took any long-acting interferon or ribavirin
2. Patients without any post-baseline safety assessment

Additional criteria might be considered when writing the Statistical Analysis Plan.

8.2 Primary and Secondary Study Variables

8.2.1 Primary Variables

In accordance with the two primary objectives of the trial (see Section 2.1), two primary variables are defined:

- Time from start of study treatment to first safety related dose reduction or treatment discontinuation of long-acting interferon or ribavirin is the primary safety endpoint.

Dose reductions or treatment discontinuations are safety related, if the reason was an adverse event or a laboratory abnormality. Dose reductions and discontinuation for other reasons (e.g. dose reductions by error or non-compliance, and treatment discontinuations due to withdrawn consent or insufficient efficacy) will not be considered as safety related.

- SVR rate defined as percentage of subjects with HCV RNA <50 IU/mL (as measured by a commercially available PCR test) at their last HCV RNA measurement at least 20 weeks (i.e. ≥140 days) post completion of the actual treatment period is the primary efficacy endpoint.

8.2.2 Secondary Variables

Secondary safety endpoints are as follows:

- Time from start of study treatment to first safety related dose reduction / treatment discontinuation of the long-acting interferon
- Time from start of study treatment to first safety related dose reduction or treatment discontinuation of ribavirin
- Degree of dose reductions expressed as percentage of the actual exposure in relation to the target exposure for
  a) the cumulative dose of long-acting interferon
  b) the cumulative dose of ribavirin
  c) the duration of treatment.
  d) each of the above restricted to the first 12 treatment weeks
- Percentage of missed treatment days in relation to the target number of treatment administrations for
  a) long-acting interferon
  b) ribavirin
  c) each of the above restricted to the first 12 treatment weeks
- Change in hemoglobin, neutrophil count and platelets from baseline to treatment weeks 2 and 4
- Change in body weight from baseline
- Serious and non-serious adverse event rate and profile separately for treatment naïve, treatment experienced and HIV co-infected subjects
- Serum ALT and ALT ratio

Secondary efficacy endpoints are as follows:

- Virological response (HCV RNA <50 IU/mL) at various on-treatment time points and end of treatment.
- Virological response during the first 12 weeks categorized as follows
  - (a) Percentage of subjects with RVR defined as HCV RNA <50 IU/mL at study week 4 (i.e. HCV RNA value <50 IU/mL within study days 26-32)
  - (b) Percentage of subjects with complete early virological response (cEVR) defined as HCV RNA <50 IU/mL at study week 12 (study day 72– 99), but no HCV RNA <50 IU/mL at week 4 (study day 26-32)

- (c) Percentage of subjects with partial early virological response (pEVR) defined as at least a 2 log drop of HCV RNA at study week 12 (study day 72– 99), but no HCV RNA <50 IU/mL at weeks 4 (study day 26-32) and 12 (study day 72– 99)
- (d) none of the above

8.3 Statistical and Analytical Methods

8.3.1 Analysis Plan

Demographic data, disease characteristics and other pre-study data will be summarized as mean, standard deviation, standard error of the mean, median, interquartile range and range for continuous variables, and in tables of frequencies and percentages for categorical variables.

The primary and secondary efficacy endpoints will be analyzed both for the ITT and the standard population. The primary and secondary safety endpoints will be analyzed for the safety population.

8.3.1.1 Analysis of Primary Safety Endpoint

Cox proportional hazard models will be used to examine the predictive value of various baseline factors on the primary safety variable 'time to first safety related dose reduction or treatment discontinuation of the long-acting interferon or ribavirin'. The time will be calculated as days from first study treatment to the day of the first dose reduction or discontinuation due to safety reasons. If a patients discontinues the study treatment for any other reasons prior to occurrence of the target event, then the time will be considered censored at the last treatment day.

Demographic data, baseline disease characteristics and other pre-study data listed in Table 1 will be examined in the Cox proportional hazard model as potential explanatory factors for time to first safety related dose reduction or treatment discontinuation. In addition, the country or region of the subject, the initial interferon dose and the initial ribavirin dose in the current study will be considered as explanatory baseline factors.

Various on-treatment factors (e.g. hemoglobin values during the study, or dose changes due to other reasons than safety) could have an impact on the risk for dose reductions or discontinuations due to safety reasons. Therefore additional Cox proportion hazard models will be applied considering those variables as time-dependent covariates.

Furthermore early safety endpoints like changes in hemoglobin, neutrophil and platelet counts from baseline to weeks 2 and 4 will be considered in a Cox regression landmark analysis together with other relevant pre-treatment factors. The landmark will be set at the upper time window of the week 4 interval specified in Table 3.

Final Cox proportional models will be developed using the following approach. In the first step all relevant explanatory covariates will be examined in univariate models. In the following stepwise and backward selection procedures only those factors will be considered, which were significant at a level of 0.25 in the univariate analysis. This will allow to exclude irrelevant factors with a high number of missing values, because in the stepwise or backward selection process only subjects with a complete set of explanatory variables can be included. In the stepwise model building process, a variable will be added to the model if the adjusted chi-square statistic is significant at the 0.1 level and a variable will be deleted from the model (both for

stepwise and backward selection) if the Wald chi-square statistic is not significant at the 0.05 level.

8.3.1.2 Analysis of Secondary Safety Endpoint

Time to event data like 'time to first safety related dose reduction or discontinuation of the long-acting interferon' will be analyzed with the same method, as described for the primary safety endpoint.

The main focus of several secondary safety variables specified in Section 8.2.2 is to analyze their impact on SVR. These analyses are described in Section 8.3.1.3. If one of these secondary safety variables is identified to have a strong negative impact on SVR (e.g. percentage of missed doses within the first 12 weeks), further explanatory analyses could be performed to identify possible predictors for this on-treatment variable (e.g. using multiple [logistic] regression analysis).

In addition, continuous secondary safety variables (exposure data and safety laboratory data) will be summarized using simple descriptive statistics (mean, standard deviation, standard error of the mean, median, interquartile range, range and 95% confidence intervals for the mean), whereas appropriate frequency tables will be provided for categorical and ordinal safety variables.

For further standard safety analysis (e.g. adverse events or laboratory abnormalities) see Section 8.3.1.6.

8.3.1.3 Analysis of Primary Efficacy Endpoint

The primary efficacy objective of the trial is to investigate the impact of safety related dose reductions or treatment discontinuations on SVR. SVR is defined as HCV RNA <50 IU/mL at the last post-treatment measurement at least 140 days after discontinuation of study treatment.

Since treatment discontinuations due to lack of EVR have a large impact on SVR rates, patients with treatment discontinuations due to insufficient virological response or other non-safety reasons will be excluded from the statistical analysis regarding the primary efficacy objective. Multiple logistic regression (MLR) methods with SVR as dependent variable and various baseline and treatment exposure covariates will be used to investigate their impact on SVR. The following exposure covariates will be investigated:

- Time from start of study treatment to first safety related dose reduction or treatment discontinuation of long-acting interferon or ribavirin
- Time from start of study treatment to first safety related dose reduction or treatment discontinuation of long-acting interferon
- Time from start of study treatment to first safety related dose reduction or treatment discontinuation of ribavirin
- Percentage of treatment exposure of long-acting interferon in relation to the target dose
- Percentage of treatment exposure of ribavirin in relation to the target dose
- Percentage of treatment duration in relation to the target treatment duration

- Percentage of missed long-acting interferon treatment days in relation to the target number of administrations
- Percentage of missed ribavirin treatment days in relation to the target number of treatment days
- Any of the variables above restricted to the first 12 weeks of treatment

Other treatment exposure covariates could be created during the analysis or specified in the statistical analysis plan. Generalized additive models (GAM) and receiver operating characteristics (ROC) curve analysis will be used to search for appropriate cut-offs of the above mentioned covariates for prediction of SVR.

8.3.1.4 Analysis of Secondary Efficacy Endpoint

Since treatment discontinuation is usually recommended in case of lack of virological response at week 12, and thus in those patients no SVR can be expected, it will be investigated, whether dose reductions in the first 12 weeks have an impact on EVR at week 12 (at least a 2 log drop in HCV RNA from baseline). The same type of analysis specified in Section 8.3.1.3 for SVR will be performed to analyze the impact of early safety related dose reductions and treatment discontinuations on EVR.

In addition to SVR the virological response (HCV RNA <50 IU/mL) over time will be summarized for the relevant time points specified in Table 3 and various subgroups specified in Section 8.3.1.5. Due to the non-interventional design of the study HCV RNA measurements might be missing at various time points. The following replacement method will be used for the determination of virological response (HCV RNA <50 IU/mL) during the treatment period in case of missing measurements: If the last available HCV RNA measurement and the next available HCV RNA measurement is <50 IU/mL, then it is assumed that the HCV RNA value was also <50 IU/mL at the interim time point with the missing measurement. For a missing HCV RNA measurement at end of treatment (EoT) a backward imputation approach will be used, i.e. for patients with SVR and a missing EoT measurement, HCV RNA <50 IU/mL will be assumed at EoT. In all other cases, subjects without measurements at the respective time points will be considered non-responders.

The positive and negative predictive values of RVR or EVR on the achievement of SVR will be analyzed for various subgroups as specified in Section 8.3.1.5:

The following criteria of RVR and EVR will be explored:

- HCV RNA <50 IU/mL at week 4 (study day 26-32)
- HCV RNA <50 IU/mL at week 12 (study day 72-99)
- HCV RNA <50 IU/mL or unquantifiable (below lower limit of quantification) or at least a 2 log drop from baseline at week 12 (study day 72-99).

The probability that the subject who develops an EVR will achieve SVR is called the positive predictive value (PPV) of the EVR for SVR. The probability that the subject who fails to develop

an EVR also will fail to achieve SVR is called the negative predictive value (NPV) of the EVR for SVR. An example using data is given in Table 4, PPV is calculated as a/(a+c). NPV is calculated as d/(b+d).

Table 4 Cross-tabulation of Early Virological Response and SVR

Number of subjects	Clearance	No clearance	Total
EVR			
YES	a	c	a + c
NO	b	d	b + d
TOTAL	a + b	c + d	a + b + c + d

Note: a, b, c, d are number of subjects in each cell

For all PPV and NPV 95% confidence intervals will be calculated.

In addition to the PPV and NPV for the variables listed above the PPV for virological response during the first 12 weeks categorized as follows will be determined.

a) Percentage of subjects with RVR defined as HCV RNA <50 IU/mL at study week 4 (i.e. HCV RNA value <50 IU/mL within study days 26-32)

b) Percentage of subjects with complete early virological response (cEVR) defined as HCV RNA <50 IU/mL at study week 12 (study day 72–99), but no HCV RNA <50 IU/mL at week 4 (study day 26-32)

c) Percentage of subjects with partial early virological response (pEVR) defined as at least a 2 log drop of HCV RNA at study week 12 (study day 72–99), but no HCV RNA <50 IU/mL at weeks 4 (study day 26-32) and 12 (study day 72–99)

d) none of the above

8.3.1.5 Subgroup Analyses

The following subgroup analyses will be performed for the primary efficacy endpoint and key secondary efficacy endpoints:

- Genotype 1, 2, 3, 4 and other
- Gender (male vs. female)
- Age groups
- Body weight groups
- BMI (<25, 25-30, >30)

- Treatment naïve patients vs. pegylated interferon plus ribavirin experienced patients vs. other INF experienced patients
- Patients with relapse to prior therapy vs. patients with breakthrough to prior therapy vs. non-response to prior therapy
- Subjects with and without cirrhosis /transition to cirrhosis
- Subjects with HIV co-infection
- High vs. low HCV RNA level at baseline using appropriate cut-offs (400,000 and 800,000 IU/mL)
- ALT ratio (<1, 1-3, >3)
- Appropriate combinations of the factors above, if the sample size is sufficient (e.g. patients with non-response to prior pegylated interferon plus ribavirin therapy by genotype).

Further subgroups might be specified in the Statistical Analysis Plan.

8.3.1.6 Standard Safety Data Analysis

Adverse events (AEs) will be assigned preferred terms and categorized into body systems according to the Medical Dictionary for Drug Regulatory Affairs (MedDRA) classification of the World Health Organization (WHO) terminology.

The proportion of subjects with AEs will be calculated by dividing the number of subjects who experienced the AE by the number of subjects evaluable for safety analysis. AEs will be summarized by treatment duration (e.g. 24 vs. 48 vs. 72 weeks) and level of pre-treatment (treatment naïve, treatment experienced), body system and event within each body system. Further subgroup analyses for safety (e.g. non-responders to previous pegylated interferon plus ribavirin combination therapy) will be specified in the statistical analysis plan.

Laboratory safety variables will be analyzed according to Roche’s “International Guideline for the Handling and Reporting of Laboratory Data” [48]. These values will be converted from any units not Systeme International (SI) to SI units. Lab data will therefore be processed and reported in terms of SI units. For those safety laboratory tests lacking sufficiently common procedures and having a wide variation of normal ranges at the sites, such as enzyme tests (ALT and TSH), the results will be transformed to the Roche standard reference range at the time of conversion to SI units in order to allow comparisons of patients from different centers. Since the standard reference ranges of most of these parameters have a lower limit of zero, only the upper limits of the ranges will be used as specified in the following equation:

$$R_T = R_U \frac{S_H}{I_H}$$

Where: R_T = transformed result

R_U = untransformed result

S_H = upper limit of Roche standard reference range

I_H = upper limit of investigator's reference range

Changes in ALT and ALT-ratio from baseline will be summarized by the disease group using descriptive statistical methods.

Laboratory parameters for which the normal range is usually differentiated by sex, such as hemoglobin, the values will be transformed to the male reference range using the following equation:

$$R_T = M_L + \frac{R_U - F_L}{F_H - F_L} (M_H - M_L)$$

Where: R_T = transformed result

R_U = untransformed result

M_L and M_H = lower and upper limits of Roche's male reference range

F_L and F_H = lower and upper limit of Roche's female reference range

Changes in laboratory from baseline and abnormalities in laboratory data will be summarized using descriptive statistical methods.

8.3.1.7 Interim Analysis

It is planned to perform interim analyses, each considering only those subjects, who have started long-acting interferon plus ribavirin treatment prior to an interim specific enrollment cut-off. Furthermore, only those study variables will be analyzed in the interim analysis, which could have been measured prior to the interim specific data cut-off. For example a first interim analysis will focus on the impact of safety related dose reductions and discontinuations within the first 12 weeks of treatment on EVR (HCV RNA <50 IU/mL at week 12).

8.4 Sample Size

For the samples size calculation the following assumptions are made. The event rate of safety related dose reductions or treatment discontinuations is expected to be in the range of 20 to 40%. The standard deviation of an explanatory covariate X_1 of interest (e.g. gender or body weight) is assumed to be in the range of 0.4 to 0.5. For a binary covariate X_1 the standard deviation is the square root of p(1-p) and thus between 0.4 and 0.5 if the proportion p of either of the binary

values of X_1 is between 0.2 and 0.5 in the population analyzed. For a continuous covariate recorded in a certain unit (e.g. body weight in kg) the SD can be converted to a value from 0.4 to 0.5 by changing the unit of the covariate X_1 (e.g. body weight per 0.5 kg or 5 kg). Furthermore, it is assumed that a risk reduction by 25% (equivalent to a hazard ratio of 0.75 or a regression coefficient of ln(HR) = -0.2877) for a 1 unit change of a covariate X_1 should be detected with 80% power. Since multiple covariates should be included into the Cox regression model an R^2 of 0.1 to 0.2 is assumed for the multiple regression of the covariate X_1 on other covariates included. The sample size required for the various scenarios is given in Table 5.

Table 5 Sample Size for Cox Proportional Hazard Analysis

Basic assumptions: alpha = 0.05, power = 80%, hazard ratio = 0.75

SD of explanatory covariate X_1	R^2 of X_1 with other Xs.	Event rate for X_1 (%)	N Patients
**0.4**	**0.2**	40	1853
		**30**	**2470**
		20	3705
0.4	0.1	40	1647
		30	2196
		20	3293
0.5	0.2	40	1186
		30	1581
		20	2371
0.5	0.1	40	1054
		30	1405
		20	2108

In summary, a sample size of 2,500 evaluable patients is required to detect a hazard ratio of 0.75 with 80% power at a significance level of 0.05 under the assumption that the standard deviation for a covariate of interest X_1 is 0.4, and R^2 of X_1 with other Xs is 0.2. In total 3,000 patients should be included to take into account patients with missing values, which can not be considered in the multivariable Cox regression model.

If the Cox regression analysis will be performed in subgroups of the total study population, then only larger effects can be detected when all other assumptions remain unchanged. For example a sample size of 426 evaluable patients is needed to detect a hazard ratio of 0.5 with 80% power at a significance level of 0.05 under the assumption that the standard deviation for a covariate of interest X_1 is 0.4, and R^2 of X_1 with other Xs is 0.2.

9. DATA COLLECTION, MANAGEMENT AND QUALITY ASSURANCE

Data for this study will be recorded via an Electronic Data Capture (EDC) system using web-based electronic Case Report Forms (eCRFs). It will be transcribed by the site from the paper source documents onto the eCRF.

Accurate and reliable data collection will be assured by verification and cross–check of the eCRFs against the investigator's records by the study monitor (source document verification) following Good Pharmacoepidemiology Practices (GPP) Guidelines [49]. Source document verification will be conducted in 10 – 15% of observed subjects in each participating center.

A comprehensive validation check program utilizing front-end checks in the eCRF and back-end checks in the database will verify the data. Discrepancies and queries will be generated accordingly in the eCRF for online resolution by the investigator at the site.

In addition the eCRF data will be reviewed on an ongoing basis for medical and scientific plausibility.

10. REFERENCES

1. Lavanchy D. Global surveillance and control of hepatitis C: report of a WHO consultation. J Viral Hepat 1999;6(1):35-47.

2. Consensus statement: EASL international consensus conference on hepatitis C. J Hepatology 1999;30:956-61.

3. Eurosurveillance 8(5), 99-118. 5-5-2003.

4. Hoofnagle JH. Course and outcome of hepatitis C. Hepatology 2002;36(5 Suppl 1):S21-9.

5. Marcellin P. Hepatitis C: the clinical spectrum of the disease. J Hepatol 1999;31(Suppl 1):9-16.

6. Brown RS. Hepatitis C and liver transplantation. Nature 2005;436(7053):973-8.

7. DeFrancesco R, Migliaccio G. Challenges and successes in developing new therapies for hepatitis C. Nature 2005;436(7053):953-60.

8. Williams I. Epidemiology of hepatitis C in the United States. Am J Med 1999;107(6B):2S-9.

9. Strader DB, Wright T, Thomas DL, Seeff LB. AASLD practice guidelines: diagnosis, management, and treatment of hepatitis C. Hepatology 2004;39:1147-70.

10. Dienstag LJ, McHutchison JG. American Gastroenterological Association technical review on the management of hepatitis C. Gastroenterology 2006;130:231–264.

11. Ghany MG, Strader DB, Thomas DL, Seeff LB. AASLD practice guidelines diagnosis, management, and treatment of hepatitis C: an update. Hepatology 2009:49:1335-1374.

12. McHutchison JG, Gordon SC, Schiff ER, Shiffman ML, Lee WM, Rustgi VK, et al. Interferon alfa-2b alone or in combination with ribavirin as initial treatment for chronic hepatitis C. Hepatitis Interventional Therapy Group. N Engl J Med 1998;339:1485-1492.

13. Zeuzem S, Feinman SV, Rasenack J, Heathcote EJ, Lai MY, Gane E, et al. Peginterferon α-2a in patients with chronic hepatitis C. N Engl J Med. 2000; 343: 1666-1672.

14. Fried MW, Shiffman ML, Reddy KR, Smith C, Marinos G, Goncales FG, et al. for the PEGASYS® International Study Group. Peginterferon alfa-2a plus ribavirin for chronic hepatitis C virus infection. N Engl J Med 2002;347(13):975-982.

15. Manns MP, McHutchison JG, Gordon SC, Rustgi VK, Shiffman M, Reindollar R, et al. PegInterferon alfa-2b plus ribavirin compared with interferon alfa-2b plus ribavirin for initial treatment of chronic hepatitis C: a randomized trial. The Lancet 2001;358:958-65.

16. Poynard T, McHutchison J, Goodman Z, Ling M, Albrecht J. Is "À la carte" combination interferon alfa-2b plus ribavirin regimen possible for the first line treatment in patients with chronic hepatitis C? Hepatology 2000;31:211-8.

17. Zeuzem S, Pawlotsky JM, Lukasiewicz E, von Wagner M, Goulis I, Lurie Y, et al. DITTO-HCV Study Group. International, multicenter, randomized, controlled study comparing dynamically individualized versus standard treatment in patients with chronic hepatitis C. J Hepatol 2005 Aug;43(2):250-7.

18. Yee HS, Currie SL, Darling JM, Wright TL. Management and treatment of hepatitis C viral infection: recommendations from the Department of Veterans Affairs Hepatitis C Resource Center program and the National Hepatitis C Program office. Am J Gastroenterol 2006 Oct;101(10):2360-78.

19. McCaughan GW, Omata M, Amarapurkar D, Bowden S, Chow WC, Chutaputti A. Asian Pacific Association for the Study of the Liver consensus statements on the diagnosis, management and treatment of hepatitis C virus infection. Asian Pacific Association for the Study of the Liver (APASL) Hepatitis C Working Party. J Gastroenterol Hepatol. 2007;22(5):615-33.

20. Manns MP, Wedemeyer H, Cornberg M. Treating viral hepatitis C: efficacy, side effects, and complications. Gut 2006 Sep;55(9):1350-9.

21. Hadziyannis SJ, Sette H, Jr., Morgan TR, Balan V, Diago M, Marcellin P, et al. Peginterferon-alpha 2a and ribavirin combination therapy in chronic hepatitis C: a randomized study of treatment duration and ribavirin dose. Ann Intern Med 2004 Mar 2;140(5):346-55.

22. European Public Assessment Report: summary of product characteristics. European Medicines Agency; 2009 May 11. Report No.: 08/04/2009 Pegasys-H-C-395-II-38.

23. European Public Assessment Report: Summary of product characteristics. European Medicines Agency; 2008 Aug 22. Report No.: 05/12/2008 Pegintron-H-C-280-II-82.

24. Shiffman ML, Suter F, Bacon BR, Nelson D, Harley H, Sola R, et al. for the ACCELERATE investigators. Peginterferon alfa-2a and ribavirin for 16 or 24 weeks in HCV genotype 2 or 3. N Engl J Med 2007 Jul 12;357(2):124-34.

25. von Wagner M, Huber M, Berg T, Hinrichsen H, Rasenack J, Heintges T, et al. Peginterferon-alpha-2a (40KD) and ribavirin for 16 or 24 weeks in patients with genotype 2 or 3 chronic hepatitis C. Gastroenterology 2005 Aug;129(2):522-7.

26. Ferenci P, Laferl H, Scherzer TM, Gschwantler M, Maieron A, Brunner H, et al. Peginterferon alfa-2a and ribavirin for 24 weeks in hepatitis C type 1 and 4 patients with rapid virological response. Gastroenterology 2008 Aug;135(2):451-8.

27. Zeuzem S, Buti M, Ferenci P, Sperl J, Horsmans Y, Cianciara J, et al. Efficacy of 24 weeks treatment with peginterferon alfa-2b plus ribavirin in patients with chronic hepatitis C infected with genotype 1 and low pretreatment viremia. J Hepatol 2006 Jan;44(1):97-103.

28. Jensen DM, Marcellin P, Freilich B, Andreone P, Di BA, Brandao-Mello CE, et al. Re-treatment of patients with chronic hepatitis C who do not respond to peginterferon-alpha2b: a randomized trial. Ann Intern Med 2009 Apr 21;150(8):528-40.

29. Morrill JA, Shrestha M, Grant RW. Barriers to the treatment of hepatitis C. Patient, provider, and system factors. J Gen Intern Med 2005 Aug;20(8):754-8.

30. Dharia TD, Malet PF, Cuthbert J. Efficacy of Chronic Hepatitis C (CHC) Treatment in an Urban, County Hospital Population Using Strict Screening Criteria. Hepatology 2008; 48 Suppl 4: 903A (AASLD 2008, Abstract 1333).

31. Khokhar OS, Lewis JH. Reasons why patients infected with chronic hepatitis C virus choose to defer treatment: do they alter their decision with time? Dig Dis Sci 2007 May;52(5):1168-76.

32. Zehnter E, Mauss S, Boeker K, Lutz T, Racky S, Schmidt W. Treatment of chronic hepatitis C with peginterferon alfa-2a (40kd) (PEG) and ribavirin (RBV) in naïve patients with HIV-HCV co-infection in the real-life setting in Germany. Hepatology 2007;46 Suppl 4:362A (AASLD 2007, Abstract 276).

33. Reddy KR, Messinger D, Popescu M, Hadziyannis SJ. Peginterferon alfa-2a (40kDa) and ribavirin: comparable rates of sustained virological response in sub-sets of older and younger HCV genotype 1 patients. J Viral Hepat 2009 Mar 25.

34. Ikeda K, Arase Y, Kawamura Y, Yatsuji H, Sezaki H, Hosaka T, et al. Necessities of interferon therapy in elderly patients with chronic hepatitis C. Am J Med 2009 May;122(5):479-86.

35. Bruno S, Hadziyannis SJ, Shiffman M, Messinger D, Marcellin P. Baseline and on-treatment factors associated with high rates of sustained virological response in patients with and without cirrhosis following treatment with peginterferon alfa-2a and ribavirin. J Hepatol 2008; 48 Suppl 2: S289 (EASL 2008, Abstract 774).

36. Schaefer M, Sarkar R; Spengler U; Schlaepfer T; Ockenga J, Friebe A. Es-Citalopram for the prevention of PEG-IFN-α and Ribavirin associated depression in HCV-infected patients without psychiatric risk factors (CIPPAD-study). Hepatology 2008; 48 Suppl 4: 119A (AASLD 2008, Abstract 270).

37. Bezemer G, Van Gool AR, Drenth JP, Hansen BE, Droogleever HA, Fortuyn CJ. A double blind, placebo-controlled trial with escitalopram to prevent psychiatric adverse events during treatment with pegylated interferon-alpha and ribavirin for chronic hepatitis C: The "Prevention Of Psychiatric Side effects (POPS)-study" Hepatology 2008; 48 Suppl 4: 1139A (AASLD 2008, Abstract 1858).

38. Lang JP, Melin P, Ouzan D, Rotily M, Fontanges T, Marcellin P. Assessment of the impact of psychiatric disorders on safety, compliance, and sustained virological response after hepatitis C treatment (CHEOBS). Hepatology 2007; 46 Suppl 4:353A (AASLD 2007, Abstract 258).

39. Melin P, Lang J, Ouzan D, Chousterman M, Varastet M, M. Rotily M. Impact of the use of drugs and substitution treatments on the antiviral treatment of chronic hepatitis C (HCV): analysis of compliance, virological response and quality of life (Cheobs). Hepatology 2008; 48 Suppl 4: 852A (AASLD 2008, Abstract 1222).

40. Mauss S, Hüppe D, Zehnter E, Manns MP, Teuber G. Opioid maintenance therapy is not associated with treatment failure to hepatitis C therapy in a large German multicentre cohort. Hepatology 2007; 46 Suppl 4:351A (AASLD 2007, Abstract 253).

41. Reddy KR, Shiffman ML, Morgan TR, Zeuzem S, Hadziyannis S, Hamzeh FM, et al. Impact of ribavirin dose reductions in hepatitis C virus genotype 1 patients completing peginterferon alfa-2a/ribavirin treatment. Clin Gastroenterol Hepatol 2007 Jan;5(1):124-9.

42. Shiffman ML, Ghany MG, Morgan TR, Wright EC, Everson GT, Lindsay KL, et al. Impact of reducing peginterferon alfa-2a and ribavirin dose during retreatment in patients with chronic hepatitis C. Gastroenterology 2007 Jan;132(1):103-12.

43. McHutchison JG, Manns M, Patel K, Poynard T, Lindsay KL, Trepo C, et al. Adherence to combination therapy enhances sustained response in genotype-1-infected patients with chronic hepatitis C. Gastroenterology 2002 Oct;123(4):1061-9.

44. Reau N, Hadziyannis SJ, Messinger D, Fried MW, Jensen DM. Early predictors of anemia in patients with hepatitis C genotype 1 treated with peginterferon alfa-2a (40KD) plus ribavirin. Am J Gastroenterol 2008 Aug;103(8):1981-8.

45. Sulkowski M, Shiffman M, Afdhal N, Reddy R, McCone J, Lee W. Hemoglobin decline is associated with SVR among HCV genotype 1—infected persons treated with peginterferon (PEG)/ribavirin (RBV): analysis from the IDEAL study. J Hepatol 2009;50 Suppl 1: S51 (EASL 2009, Abstract 126).

46. Weiss JJ, Brau N, Stivala A, Swan T, Fishbein D. Adherence to medication for chronic hepatitis C: Building on the model of human immunodeficiency virus antiretroviral adherence research. Aliment Pharmacol Ther 2009 Mar 26;30:1,14-27.

47. Pearlman B, Ehleben C, Lovett L. Hepatitis C Virus (HCV) Virologic Response Correlates With Therapy-Induced Cytopenias And Side Effects. Hepatology 2008;48 Suppl 4: 870A (AASLD 2008, Abstract 1262).

48. Roche Pharma Global Development PD GCP supporting document gcp_spt000144: International standard for the handling and reporting of laboratory data - Clinical Operating Guideline No. 3007. December 13, 2005.

49. Epstein M. International Society of Pharmacoepidemiology. Guidelines for good pharmacoepidemiology practices (GPP). Pharmacoepidemiol Drug Saf 2005 Aug;14(8):589-95.

Appendix 1 Child-Pugh Classification of Severity of Liver Disease

Modified Assessment

Assessment	*Degree of abnormality*	*Score*
Encephalopathy	None<br>Grade 1-2<br>Grade 3-4*	1<br>2<br>3
Ascites	Absent<br>Slight<br>Moderate	1<br>2<br>3
S-Bilirubin (mg/dl)<br><br><br>SI unit = µmol/l)	<2<br>2.0-3<br>>3<br><br><34<br>34-51<br>>51	1<br>2<br>3<br><br>1<br>2<br>3
S-Albumin (g/dl)	>3.5<br>3.5-2.8<br><2.8	1<br>2<br>3
INR	<1.7<br>1.7-2.3<br>>2.3	1<br>2<br>3

*grading according to Trey, Burns and Saunders (1996)

Grade **A (mild)**: Total score of 5 or 6

Grade **B (moderate)**: Total score of 7 to 9

Grade **C (severe)**: Total score of 10 to 15

PART II: ETHICS AND GENERAL STUDY ADMINISTRATION

11. ETHICAL ASPECTS

11.1 Guidelines for Epidemiological Studies

The guidelines for good pharmacoepidemiology practices (GPP) in non-interventional studies will be respected as well as recommendations for non-interventional trials and principles of epidemiology studies [49]. This trial is not in the scope of Good Clinical Practice (GCP) studies.

11.2 Informed Consent

It is the responsibility of the investigator, or a person designated by the investigator (if acceptable by local regulations), to obtain written informed consent where local regulations allow or require it from each subject participating in this cohort study, after adequate explanation of the aims, methods, anticipated benefits, and potential hazards of the observation. The investigator or designee must also explain that the subjects are completely free to refuse to enter the cohort study or to withdraw from it at any time, for any reason. The eCRF for this study contains a section for documenting informed subject consent, and this must be completed appropriately where local regulations allow or require it. Informed consent may be given up to 4 weeks after treatment initiation.

Subjects must agree to the anonymous data collection, the pooling of data with similar scientific data, and the possibility of monitoring activities between his/her subject file and the eCRF by Roche personnel or Roche contracted monitors and regulatory personnel.

Appropriate forms for subject information and obtaining written informed consent will be provided by the investigator or by Roche/designee.

11.3 Independent Ethics Committees/Institutional Review Board

<u>Independent Ethics Committees (non-US)</u>

This observational plan and any accompanying material provided to the subject (such as subject information sheets or descriptions of the study used to obtain informed consent) as well as any advertising or compensation given to the subject, will be submitted by the investigator to an Independent Ethics Committee (IEC). Approval from the committee must be obtained before starting the study, and should be documented in a letter to the investigator specifying the date on which the committee met and granted the approval.

Any modifications made to the observational plan after receipt of the IEC approval must also be submitted by the investigator to the Committee in accordance with local procedures and regulatory requirements.

When no local review board exists, the investigator is expected to submit the observational plan to a regional committee. If no regional committee exists, Roche will assist the investigator in submitting the observational plan to the European Ethics Review Committee.

12. CONDITIONS FOR MODIFYING THE OBSERVATIONAL PLAN

Observational plan modifications to ongoing studies must be made only after consultation between an appropriate representative of the sponsor and the investigator. Observational plan modifications must be prepared by a representative of the sponsor and initially reviewed and approved by the International Medical Leader and Biostatistician.

All observational plan modifications must be submitted to the appropriate IEC or Institutional Review Board (IRB) for information and approval in accordance with local requirements, and to Regulatory Agencies if required. Approval must be awaited before any changes can be implemented, except for changes necessary to eliminate an immediate hazard to trial subjects, or when the change(s) involves only logistical or administrative aspects of the trial (e.g. change in monitor(s), change of telephone number(s).

13. CONDITIONS FOR TERMINATING THE STUDY

Both the sponsor and the investigator reserve the right to terminate the study at any time. Should this be necessary, both parties will arrange the procedures on an individual study basis after review and consultation. In terminating the study, Roche and the investigator will assure that adequate consideration is given to the protection of the subject's interests.

14. STUDY DOCUMENTATION, eCRFS AND RECORD KEEPING

14.1 Investigator's Files / Retention of Documents

The Investigator must maintain adequate and accurate records to enable the conduct of this cohort study and the study data to be subsequently verified. These documents should be classified into two different separate categories (1) Investigator's Study File, and (2) subject clinical source documents.

The Investigator's Study File will contain the observational plan/amendments, IEC/IRB and governmental approval with correspondence, sample informed consent, staff curriculum vitae and authorization forms and other appropriate documents/correspondence etc.

Subject clinical source documents would include subject hospital/clinic records, physician's and nurse's notes, appointment book, original laboratory reports, ECG, EEG, X-ray, pathology and special assessment reports, signed informed consent forms, consultant letters, and subject screening and enrollment logs. The Investigator must keep these two categories of documents on file according to local regulations after completion or discontinuation of the study. After that period of time the documents may be destroyed, subject to local regulations.

Should the Investigator wish to assign the study records to another party or move them to another location, Roche must be notified in advance.

If the Investigator can not guarantee this archiving requirement at the investigational site for any or all of the documents, special arrangements must be made between the Investigator and Roche to store these in a sealed container(s) outside of the site so that they can be returned sealed to the Investigator in case of a regulatory audit. Where source documents are required for the continued care of the subject, appropriate copies should be made for storing outside of the site.

14.2 Source Documents and Background Data

The investigator shall supply the sponsor on request with any required background data from the study documentation or clinic records. This is particularly important when errors in data transcription are suspected. In case of special problems and/or governmental queries or requests for audit inspections, it is also necessary to have access to the complete study records, provided that subject confidentiality is protected.

14.3 Audits and Inspections

The investigator should understand that source documents for this trial should be made available to appropriately qualified personnel from the Roche Research Quality Assurance Unit or its designees, or to health authority inspectors after appropriate notification. The verification of the data entered into the eCRF data must be by direct inspection of source documents.

14.4 Electronic Case Report Forms

Data for this study will be entered into an eCRF via a web-based EDC system. For each subject enrolled, an eCRF must be completed by the principal investigator or an authorized delegate from the study staff. This also applies to records for those subjects who fail to complete the study. If a subject withdraws from the study, the reason must be noted on the eCRF.

The investigator should ensure the accuracy, completeness and timeliness of the data reported to the sponsor on the eCRF and in all required reports.

Access to the EDC system will be controlled through the use of unique individual user names and passwords for all entitled study personnel. The investigator will have continual oversight of the electronically reported data of his/her site. Study Clinical Research Associate (CRA) will review study data in the EDC system on an ongoing basis to identify and address data quality issues (e.g. observational plan violations) and to ensure the site is continuously entering data into the EDC system. During site monitoring visits, the CRA will verify the data contained in the EDC system against the source documentation and medical records maintained by the investigator.

The system will maintain a full audit trail of entered and changed data along with data on the user.

14.5 Contract Research Organizations

This observational plan was set up in cooperation with Dr. med. Ulrike Hauf-Zachariou, Dip.Pharm.Med., MFPM RCP (UK), IST GmbH, Soldnerstrasse 1, 68219 Mannheim, Germany, tel.: +49 621 8798622, fax:+49 621 8798690.

15. MONITORING THE STUDY

It is understood that the responsible Roche monitor (or designee) will contact and visit the investigator regularly and will be allowed, on request, to inspect the various records of the trial provided that subject confidentiality is maintained in accord with local requirements.

It will be the monitor's responsibility to inspect the electronic Case Report Forms for all subjects at regular intervals throughout the study, to verify the adherence to the observational plan and the completeness, consistency and accuracy of the data being entered on them. The monitor should have access to laboratory test reports and other subject records needed to verify the entries on the eCRF. The investigator (or his/her deputy) agrees to cooperate with the monitor to ensure that any problems detected in the course of these monitoring visits are resolved.

Source data verification may be conducted in 10-15% of subjects in each participating center. The verification of the eCRF data must be by direct inspection of source documents for the selected subjects.

16. CONFIDENTIALITY OF TRIAL DOCUMENTS AND SUBJECT RECORDS

The investigator must assure that subjects' anonymity will be maintained and that their identities are protected from unauthorized parties. On eCRFs or other documents submitted to the sponsor, subjects should not be identified by their names, but by an identification code. The investigator should keep a subject enrollment log showing codes, names and addresses. The investigator should maintain documents not for submission to Roche, e.g., subjects' written consent forms, in strict confidence.

17. PUBLICATION OF DATA AND PROTECTION OF TRADE SECRETS

The results of this study may be published or presented at scientific meetings. If this is foreseen, the investigator agrees to submit all manuscripts or abstracts to Roche prior to submission. This allows the sponsor to protect proprietary information and to provide comments based on information from other studies that may not yet be available to the investigator.

In accord with standard editorial and ethical practice, Roche will generally support publication of multicenter trials only in their entirety and not as individual center data. In this case, a coordinating investigator will be designated by mutual agreement.

Any formal publication of the study in which input of Roche personnel exceeded that of conventional monitoring will be considered as a joint publication by the investigator and the appropriate Roche personnel. Authorship will be determined by mutual agreement.
