# Supplementary material for: Impact of Safety-Related Dose Reductions or Discontinuations on Sustained Virologic Response in HCV-Infected Patients: Results from the GUARD-C Cohort
Source: PLoS One. 2016 Mar 28;11(3):e0151703. doi: 10.1371/journal.pone.0151703 (PMC4809570; doi:10.1371/journal.pone.0151703)
Supplement: S3 File — (DOCX) [file pone.0151703.s003.docx]

STROBE Statement—checklist of items that should be included in reports of observational studies

|  | Item No. | Recommendation | Page  No. | Relevant text from manuscript |
| --- | --- | --- | --- | --- |
| **Title and abstract** | 1 | (*a*) Indicate the study’s design with a commonly used term in the title or the abstract | 1 (Title)  3 (abstract) | Results from the GUARD-C Cohort  The GUARD-C cohort… |
|  |  | (*b*) Provide in the abstract an informative and balanced summary of what was done and what was found | Page 3 | A total of 3181 HCV-mono-infected treatment-naive patients were assigned…  See entire abstract |
| Introduction | | | |  |
| Background/rationale | 2 | Explain the scientific background and rationale for the investigation being reported | Page 4 |  |
| Objectives | 3 | State specific objectives, including any prespecified hypotheses | Page 4 | Herein, we report the results of a large, international, noninterventional cohort (GUARD-C) that was undertaken with the objective of identifying baseline predictors of safety-related dose reductions or discontinuations (sr-RD) to manage adverse events and laboratory abnormalities and their impact on SVR rates in patients receiving peginterferon alfa/ribavirin in routine clinical practice. |
| Methods | | | |  |
| Study design | 4 | Present key elements of study design early in the paper | Page 5 | Study Design  GUARD-C is an international, prospective, observational cohort study in patients with chronic hepatitis C receiving peginterferon alfa/ribavirin combination therapy (ClinicalTrials.gov Identifier: NCT01344889). The study was conducted in 25 countries in Europe, Asia, North Africa, the Middle East, and South America (Albania, Algeria, Bahrain, Belgium, Bosnia-Herzegovina, Brazil, Egypt, Greece, Hungary, India, Iran, Italy, Kuwait, Lebanon, Former Yugoslav Republic of Macedonia, Morocco, Pakistan, Poland, Portugal, Qatar, Romania, Serbia, Slovakia, South Korea, and the United Arab Emirates). |
| Setting | 5 | Describe the setting, locations, and relevant dates, including periods of recruitment, exposure, follow-up, and data collection | Page 5  Page 10-11 | GUARD-C is an international, prospective, observational cohort study in patients with chronic hepatitis C receiving peginterferon alfa/ribavirin combination therapy (ClinicalTrials.gov Identifier: NCT01344889). The study was conducted in 25 countries in Europe, Asia, North Africa, the Middle East, and South America (Albania, Algeria, Bahrain, Belgium, Bosnia-Herzegovina, Brazil, Egypt, Greece, Hungary, India, Iran, Italy, Kuwait, Lebanon, Former Yugoslav Republic of Macedonia, Morocco, Pakistan, Poland, Portugal, Qatar, Romania, Serbia, Slovakia, South Korea, and the United Arab Emirates).  Overall, a total of 3181 treatment-naive patients with HCV mono-infection were enrolled in 25 countries, assigned to a planned treatment duration of 24 or 48 weeks and treated with peginterferon alfa/ribavirin and followed up between October 2009 and June 2013 |
| Participants | 6 | (*a*) *Cohort study*—Give the eligibility criteria, and the sources and methods of selection of participants. Describe methods of follow-up | Page 5 | Adult patients (males and non-pregnant females) with chronic hepatitis C and quantifiable serum hepatitis C virus (HCV) RNA levels receiving treatment with peginterferon alfa/ribavirin according to the standard of care and the product license were eligible for enrolment after informed consent. Patients with contraindications to peginterferon alfa/ribavirin or with end-stage renal disease, and/or recipients of major organ transplants were not eligible for the study. Enrolment in the study and the dose and duration of treatment were at the discretion of the investigator. |
|  |  | (*b*) *Cohort study*—For matched studies, give matching criteria and number of exposed and unexposed  *Case-control study*—For matched studies, give matching criteria and the number of controls per case |  | Not applicable |
| Variables | 7 | Clearly define all outcomes, exposures, predictors, potential confounders, and effect modifiers. Give diagnostic criteria, if applicable | Page 6 | The primary efficacy endpoint was SVR24, defined as HCV RNA <50 IU/mL after 24 weeks of untreated follow-up (≥140 days after last dose of treatment). Patients with missing SVR24 values were considered nonresponders. Relapse was defined as HCV RNA ≥50 IU/mL during untreated follow-up in a patient with an end-of-treatment virologic response.  Rapid virologic response (RVR) was defined as a virologic response (HCV RNA <50 IU/mL) by Week 4. Complete early virologic response (cEVR) was defined as a virologic response by Week 12, but no RVR. Partial early virologic response (pEVR) was defined as a ≥2-log10 reduction in HCV RNA by Week 12 in a patient with no RVR or cEVR.  The primary safety endpoint of the trial was the time to first sr-RD of peginterferon alfa or ribavirin. Patients were categorized by the time to first occurrence of sr-RD (no sr-RD or ≥1 sr-RD in the first 4 or 12 weeks of treatment) in order to assess the impact of sr-RDs on SVR24 rates. |
| Data sources/ measurement | 8* | For each variable of interest, give sources of data and details of methods of assessment (measurement). Describe comparability of assessment methods if there is more than one group | Page 6 | The primary efficacy endpoint was SVR24, defined as HCV RNA <50 IU/mL after 24 weeks of untreated follow-up (≥140 days after last dose of treatment). Patients with missing SVR24 values were considered nonresponders. Relapse was defined as HCV RNA ≥50 IU/mL during untreated follow-up in a patient with an end-of-treatment virologic response.  Rapid virologic response (RVR) was defined as a virologic response (HCV RNA <50 IU/mL) by Week 4. Complete early virologic response (cEVR) was defined as a virologic response by Week 12, but no RVR. Partial early virologic response (pEVR) was defined as a ≥2-log10 reduction in HCV RNA by Week 12 in a patient with no RVR or cEVR.  The primary safety endpoint of the trial was the time to first sr-RD of peginterferon alfa or ribavirin. The time will be calculated as days from first study treatment to the day of the first dose reduction or discontinuation due to safety reasons. If a patient discontinues the study treatment for any other reasons, then the time will be considered censored at the last treatment day. Patients were categorized by the time to first occurrence of sr-RD (no sr-RD or ≥1 sr-RD in the first 4 or 12 weeks of treatment) in order to assess the impact of sr-RDs on SVR24 rates. |
| Bias | 9 | Describe any efforts to address potential sources of bias |  | Comment: Cox proportional hazards analyses were performed for the primary endpoint to adjust for confounding factors.  For the primary efficacy endpoint, SVR, patients with missing assessment were considered to be non-responders to avoid overestimating response rates. |
| Study size | 10 | Explain how the study size was arrived at | Page 7 | It was assumed that the rate of sr-RD would be in the range of 20 to 40%; that the standard deviation of explanatory covariates included in the Cox proportional hazard model would range from 0.4 to 0.5 (after conversion of units) and that a risk reduction of 25% (equivalent to a hazard ratio of 0.75) for a 1-unit change in a covariate should be detected with 80% power. Since multiple covariates were to be included into the Cox regression model an R² of 0.1 to 0.2 was assumed for the multiple regression of a covariate on other covariates. On the basis of these assumptions, a sample size of 2,500 evaluable patients would be required to detect a hazard ratio of 0.75 with 80% power at a significance level of 0.05. To account for patients with missing values, a sample size of 3,000 patients was planned. |

Continued on next page

| Quantitative variables | 11 | Explain how quantitative variables were handled in the analyses. If applicable, describe which groupings were chosen and why | Page 7-8 | A total of 4453 patients were enrolled in GUARD-C and 4354 received at least one dose of study drug; however, this analysis was restricted to treatment-naive HCV mono-infected patients who were assigned to 24 or 48 weeks of treatment with peginterferon alfa-2a or -2b plus ribavirin (Fig 1). Patients with acute hepatitis C were excluded, as were those patients with chronic hepatitis C who were assigned to treatment durations longer than 48 weeks, those who had received prior treatment for chronic hepatitis C, those who had received other treatment regimens or had switched between treatment regimens, and/or those patients who were co-infected with hepatitis B virus or HIV. Sub-analyses were performed in two sub-groups: 1) treatment-naive HCV mono-infected genotype (G) 1 patients (cirrhotic and noncirrhotic); and 2) treatment-naive HCV mono-infected Caucasian, noncirrhotic G1 patients, both populations of which were assigned to 48 weeks of treatment with peginterferon alfa-2a/ribavirin. The rationale for these sub-analyses is that G1 is the most common and difficult-to-treat HCV genotype, and that 48 weeks is the recommended duration of treatment with peginterferon alfa/ribavirin in patients with G1 infection.(6) A patient selection tool for Caucasian, noncirrhotic G1 patients treated for 48 weeks with peginterferon alfa-2a/ribavirin has been developed by Ferenci et al.(11); thus, the analysis in subgroup 2 was intended to evaluate this patient selection tool in a separate population. Moreover, a meta-analysis has shown that there is a significant difference in SVR rates between patients treated with peginterferon alfa-2a and those treated with alfa-2b,(12) and between different races (especially if host IL28B genotype is unknown).(13) |
| --- | --- | --- | --- | --- |
| Statistical methods | 12 | (*a*) Describe all statistical methods, including those used to control for confounding | Pages 7-10 |  |
|  |  | (*b*) Describe any methods used to examine subgroups and interactions | Page 8 | Numbers and frequencies of variables of interest were calculated for subgroup analyses and associations between variables of interest (e.g. SVR), were investigated by tests of association (Pearson chi-square, Fisher’s exact test). |
|  |  | (*c*) Explain how missing data were addressed | Page 8-9 | Comment: For the primary safety endpoint (time to first sr-RD), censoring was used. Patients without event were censored at the last day of treatment. For the primary efficacy endpoint, SVR, patients with missing HCV RNA assessment were considered non-responders.  See page 8-9: The time was censored at the last treatment day for patients who did not have any sr-RD.  See page 6: Patients with missing SVR24 values were considered nonresponders. |
|  |  | (*d*) *Cohort study*—If applicable, explain how loss to follow-up was addressed  *Case-control study*—If applicable, explain how matching of cases and controls was addressed  *Cross-sectional study*—If applicable, describe analytical methods taking account of sampling strategy |  | Comment as noted above: Patients lost to follow-up were censored at the date of last treatment for the primary safety endpoint if they were lost to follow-up before an sr-RD. For the primary efficacy endpoint they were considered to be non-responders if no HCV RNA assessment was available for SVR. |
|  |  | (*e*) Describe any sensitivity analyses |  | Comment: No sensitivity analyses were performed. |
| Results | | | | |
| Participants | 13* | (a) Report numbers of individuals at each stage of study—eg numbers potentially eligible, examined for eligibility, confirmed eligible, included in the study, completing follow-up, and analysed | Page 7-8  Figure 1 |  |
|  |  | (b) Give reasons for non-participation at each stage | S4 Table  Page 14 |  |
|  |  | (c) Consider use of a flow diagram | Figure 1 | Figure 1 |
| Descriptive data | 14* | (a) Give characteristics of study participants (eg demographic, clinical, social) and information on exposures and potential confounders | Table 2 | Table 2 |
|  |  | (b) Indicate number of participants with missing data for each variable of interest | Caption to Fig 3 | Patients with missing data (n = 300) for risk factors were excluded (BMI n=18; HCV genotype n=23; baseline hemoglobin n=45; baseline platelets n=56; baseline neutrophils n=245). |
|  |  | (c) *Cohort study*—Summarise follow-up time (eg, average and total amount) | Page 6 | Comment: Follow-up was fixed at 24 weeks after end of treatment.  See page 6: The primary efficacy endpoint was SVR24, defined as HCV RNA <50 IU/mL after 24 weeks of untreated follow-up (≥140 days after last dose of treatment). |
| Outcome data | 15* | *Cohort study*—Report numbers of outcome events or summary measures over time | Page 14  Tables S5 & S6  Page 14  Table S7 | In the overall population, stratified by HCV genotype, the SVR24 rates for patients with HCV G1, 2, 3, and 4 infection were 46.1% (754/1634), 77.1% (279/362), 68.0% (514/756), and 51.3% (203/396), respectively. Virologic response and relapse rates by treatment regimen and genotype are presented in Tables S5 and S6.  The incidences of sr-RD of peginterferon alfa in the overall population, and in subgroups 1 and 2 were 16.9%, 21.3%, and 18.4%, respectively, and the incidences of sr-RD for ribavirin were 21.8%, 29.5%, and 28.5%, respectively (Table S7). |
|  |  | *Case-control study—*Report numbers in each exposure category, or summary measures of exposure |  |  |
|  |  | *Cross-sectional study—*Report numbers of outcome events or summary measures |  |  |
| Main results | 16 | (*a*) Give unadjusted estimates and, if applicable, confounder-adjusted estimates and their precision (eg, 95% confidence interval). Make clear which confounders were adjusted for and why they were included | Tables S5 and S6 |  |
|  |  | (*b*) Report category boundaries when continuous variables were categorized |  |  |
|  |  | (*c*) If relevant, consider translating estimates of relative risk into absolute risk for a meaningful time period |  |  |

Continued on next page

| Other analyses | 17 | Report other analyses done—eg analyses of subgroups and interactions, and sensitivity analyses | Pages 15-17  Figure 2B  Figure 4B  Figure 5  Figure 6 |  |
| --- | --- | --- | --- | --- |
| Discussion | | | | |
| Key results | 18 | Summarise key results with reference to study objectives | Page 19 | this analysis from GUARD-C shows that baseline factors can be used to identify patients at increasing risk for sr-RD, and that sr-RD have an impact on SVR24 rates. Factors associated with sr-RD in the overall population (G1–6) included female sex, older age, lower body mass index, HCV G1/4 vs. 2/3, the presence of cardiovascular and pulmonary disease and low hematological indices (hemoglobin, neutrophils, platelets). The same factors were associated with sr-RD in Caucasian noncirrhotic HCV G1 patients with the exception of age (and genotype). To our knowledge, this association between sr-RD and pre-existing pulmonary and cardiovascular disease is a novel finding.  Not surprisingly, the incidence of sr-RD increased in proportion to the number of risk factors for sr-RD. Among patients with no risk factors the incidence of sr-RD was approximately 7% and among those individuals with at least six risk factors for sr-RD the incidence was approximately 74%. Conversely, SVR24 rates were highest in patients with no risk factors for sr-RD (72.1%) and were lowest in patients with at least six risk factors for sr-RD (26.1%). |
| Limitations | 19 | Discuss limitations of the study, taking into account sources of potential bias or imprecision. Discuss both direction and magnitude of any potential bias | Pages 22-23 | Limitations of this study include those typical of cohort studies and retrospective analyses. The number of non-Caucasian patients and patients with frank cirrhosis was low, a fact which places limits on the generalizability of the results. Host IL28B genotype is the most important baseline predictor of response to peginterferon alfa.(12) As such, host genotype provides very useful information when planning treatment with peginterferon alfa/ribavirin; however, IL28B genotype is not routinely available in resource-limited settings where dual therapy is most likely to be used. The patient selection tool of Ferenci et al.(11) does not include liver biopsy results, and analysis of subgroup 2 in the present study specifically excluded patients with cirrhosis. However, as patients with cirrhosis are a group in urgent need of treatment, it seems reasonable to say that these individuals should be given a high priority for treatment with DAA-containing regimens including interferon-free combinations, regardless of their baseline predictive score. |
| Interpretation | 20 | Give a cautious overall interpretation of results considering objectives, limitations, multiplicity of analyses, results from similar studies, and other relevant evidence | Pages 18-19 | The safety and efficacy of dual peginterferon alfa/ribavirin therapy observed in GUARD-C are consistent with the results of previous large randomized trials and cohort studies. The overall SVR24 rates of 55.8% (all genotypes combined) and 46.1% in the G1 population is very similar to that achieved in the Phase 3 registration trials.(15–17) The higher SVR24 rate in G1 patients assigned to 48 weeks’ treatment with peginterferon alfa-2a/ribavirin (47.3%) than in those receiving peginterferon alfa-2b/ribavirin (28.8%) supports the results of a comprehensive meta-analysis of randomized trials.(11) The overall rate of treatment withdrawal among G1 patients assigned to 48 weeks of treatment with peginterferon alfa-2a/ribavirin was approximately 25% in the present study, which is similar to the rate reported in Phase 3 studies of peginterferon alfa-2a/ribavirin (≈30%).(16,17) In this subgroup, the most common reasons for withdrawal of treatment with peginterferon alfa-2a in the present study were lack of efficacy (≈10%) and safety (≈6%). |
| Generalisability | 21 | Discuss the generalisability (external validity) of the study results | Pages 22-25 | Patient selection is increasingly important in the treatment of chronic hepatitis C. Several DAAs are now available, and interferon-free regimens with consistently high cure rates are now a reality.(2) However, although recently approved and pending DAA-containing regimens are highly effective and have good safety profiles, they are unlikely to be universally available due to high acquisition costs.(2,3,5) For this reason dual peginterferon/ribavirin therapy is likely to remain a viable option in resource-limited settings. For clinicians who continue to use dual therapy in their practice, it is important that they remain able to make informed decisions about the suitability of treatment for a given patient. This analysis provides practical information that can be used to inform such decisions. An individual patient’s characteristics can be reviewed prior to baseline to evaluate whether they are likely to achieve an SVR24 with dual therapy, and whether they are likely to experience an sr-RD. |
| Other information | |  | | |
| Funding | 22 | Give the source of funding and the role of the funders for the present study and, if applicable, for the original study on which the present article is based | Provided via Submission system as per journal requirements | This study was funded by F. Hoffmann-La Roche Ltd.  F. Hoffmann-La Roche participated in the design and conduct of the study, in the analysis and interpretation of the data and in the preparation and review of the manuscript before submission.  Third-party medical writing assistance, provided by Health Interactions, but not editorial content development sufficient to meet International Committee of Medical Journal Editors (ICMJE) authorship criteria, was funded by F. Hoffmann-La Roche Ltd. |

*Give information separately for cases and controls in case-control studies and, if applicable, for exposed and unexposed groups in cohort and cross-sectional studies.

**Note:** An Explanation and Elaboration article discusses each checklist item and gives methodological background and published examples of transparent reporting. The STROBE checklist is best used in conjunction with this article (freely available on the Web sites of PLoS Medicine at http://www.plosmedicine.org/, Annals of Internal Medicine at http://www.annals.org/, and Epidemiology at http://www.epidem.com/). Information on the STROBE Initiative is available at www.strobe-statement.org.
