# Supplementary material for: Impact of Safety-Related Dose Reductions or Discontinuations on Sustained Virologic Response in HCV-Infected Patients: Results from the GUARD-C Cohort
Source: PLoS One. 2016 Mar 28;11(3):e0151703. doi: 10.1371/journal.pone.0151703 (PMC4809570; doi:10.1371/journal.pone.0151703)
Supplement: S1 Table — (DOCX) [file pone.0151703.s005.docx]

**S1 Table. Factors considered in the multiple logistic regression analysis of SVR.**

| **Demographic factors** | Gender |
| --- | --- |
|  | Age |
|  | Weight |
|  | Body mass index |
|  | Height |
|  | Race |
|  | Country |
| **Baseline characteristics** | HCV genotype |
|  | HCV RNA concentration in serum |
|  | Duration of infection |
|  | Cirrhosis (yes or no) |
|  | Regular alcohol consumption |
|  | Drug use |
|  | Smoking |
| **Laboratory data** | BL ALT ratio |
|  | BL platelets count |
|  | BL neutrophils count |
|  | BL hemoglobin |
|  | BL prothrombin time |
| **Concomitant diseases at baseline** | Psychiatric disease/symptoms |
|  | Diabetes mellitus |
|  | Cardiovascular disease |
|  | Pulmonary disease |
|  | Thyroid disease |
|  | Chronic skin disease |
| **Treatment characteristics** | Intended treatment duration (24 or 48 weeks) |
|  | Type of peginterferon (alfa-2a vs. alfa-2b) |
|  | Time from start of study treatment to first sr-RD of peginterferon or ribavirin |
|  | Time from start of study treatment to first sr-RD of peginterferon |
|  | Time from start of study treatment to first sr-RD of ribavirin |
|  | Percentage of treatment exposure of peginterferon in relation to target dose |
|  | Percentage of treatment exposure of ribavirin in relation to target dose |
|  | Percentage of treatment duration in relation to the target treatment duration |
|  | Percentage of missed peginterferon treatment administrations in relation to the target number of administrations |
|  | Percentage of missed ribavirin treatment days in relation to the target number of treatment days |
|  | On-treatment response |

sr-RD, safety-related dose reduction or discontinuation
