# Supplementary material for: Impact of Safety-Related Dose Reductions or Discontinuations on Sustained Virologic Response in HCV-Infected Patients: Results from the GUARD-C Cohort
Source: PLoS One. 2016 Mar 28;11(3):e0151703. doi: 10.1371/journal.pone.0151703 (PMC4809570; doi:10.1371/journal.pone.0151703)
Supplement: S2 Table — (DOCX) [file pone.0151703.s006.docx]

**S2 Table. Baseline characteristics of 2845 treatment-naive HCV mono-infected patients treated with peginterferon alfa-2a/ribavirin.**

|  | **G1** | **G2** | **G3** | **G4** | **G5/6** | **Unknown** |
| --- | --- | --- | --- | --- | --- | --- |
| **Characteristic** | **(n=1508)** | **(n=294)** | **(n=671)** | **(n=341)** | **(n=10)** | **(n=21)** |
| **Male sex, n (%)** | 809 (53.6) | 142 (48.3) | 491 (73.2) | 262 (76.8) | 8 (80.0) | 18 (85.7) |
| **Mean ± SD age, years** | 47.4 ± 12.5 | 55.7 ± 12.9 | 39.3 ± 10.6 | 43.1 ± 10.1 | 43.5 ± 18.9 | 41.6 ± 11.4 |
| **Race, n (%)** |  |  |  |  |  |  |
| Caucasian/White | 1398 (92.7) | 246 (83.7) | 492 (73.3) | 300 (88.0) | 5 (50.0) | 15 (71.4) |
| Black | 20 (1.3) | 2 (0.7) | 6 (0.9) | 29 (8.5) | – | – |
| Asian/Oriental | 77 (5.1) | 46 (15.6) | 173 (25.8) | 12 (3.5) | 5 (50.0) | 6 (28.6) |
| Other | 13 (0.9) | – | – | – | – | – |
| **Mean ± SD body mass index, kg/m^2^** | 25.9 ± 4.2 | 25.7 ± 4.2 | 25.7 ± 4.4 | 28.0 ± 4.8 | 25.4 ± 4.9 | 29.4 ± 4.1 |
| **Method to assess liver fibrosis, n (%)** |  |  |  |  |  |  |
| Biopsy | 923 (61.2) | 30 (10.2) | 217 (32.3) | 163 (47.8) | 6 (60.0) | 13 (61.9) |
| Noninvasive method | 345 (22.9) | 159 (54.1) | 172 (25.6) | 107 (31.4) | 2 (20.0) | 2 (9.5) |
| Best guess/Not assessed | 240 (15.9) | 105 (35.7) | 282 (42.0) | 71 (20.8) | 2 (20.0) | 6 (28.6) |
| **Result of liver fibrosis assessment, n (%)^a^** |  |  |  |  |  |  |
| Transition to cirrhosis/cirrhosis | 461/1505 (30.6) | 49/293 (16.7) | 100/669 (14.9) | 74/340 (21.8) | 9/10 (90.0) | 3/21 (14.3) |
| No transition to cirrhosis/cirrhosis | 1044/1505 (69.4) | 244/293 (83.3) | 569/669 (85.1) | 266/340 (78.2) | 1/10 (10.0) | 18/21 (85.7) |
| **Mean ± SD HCV RNA level, log_10_ IU/mL** | 5.86 ± 0.80 | 5.83 ± 0.94 | 5.70 ± 0.90 | 5.57 ± 0.91 | 6.15 ± 0.73 | 5.87 ± 0.98 |
| **HCV RNA >800,000 IU/mL, n (%)** | 792/1497 (52.9) | 169/291 (58.1) | 304/654 (46.5) | 136/341 (39.9) | 6/10 (60.0) | 12/20 (60.0) |
| **Mean ± SD hemoglobin conc, g/L** | 151.6 ± 13.5 | 149.1 ± 12.9 | 150.2 ± 14.5 | 150.7 ± 14.5 | 159.9 ± 12.7 | 154.8 ± 11.4 |
| **Mean ± SD neutrophil count x 10^9^/L** | 3.6 ± 1.8 | 3.2 ± 1.2 | 4.0 ± 1.6 | 3.4 ± 1.6 | 3.9 ± 1.8 | 3.1 ± 1.4 |
| **Mean ± SD platelet count x 10^9^/L** | 204.2 ± 67.2 | 201.6 ± 74.7 | 209.1 ± 66.9 | 199.4 ± 64.1 | 222.4 ± 49.2 | 190.2 ± 53.7 |
| **Mean ± SD ALT activity, IU/L** | 116.1 ± 95.5 | 120.5 ± 138.0 | 137.9 ± 103.7 | 99.0 ± 74.1 | 92.3 ± 45.5 | 115.2 ± 68.0 |
| **Mean ± SD ALT ratio** | 2.1 ± 1.7 | 2.2 ± 2.5 | 2.5 ± 1.9 | 1.8 ± 1.3 | 1.7 ± 0.8 | 2.1 ± 1.2 |
| **ALT ratio >3, n (%)** | 264/1477 (17.9) | 60/291 (20.6) | 171/662 (25.8) | 48/337 (14.2) | 1/10 (10.0) | 5/20 (25.0) |
| **Assigned treatment duration, n (%)** |  |  |  |  |  |  |
| 24 weeks | 11 (0.7) | 288 (98.0) | 636 (94.8) | 3 (0.9) | – | 6 (28.6) |
| 48 weeks | 1497 (99.3) | 6 (2.0) | 35 (5.2) | 338 (99.1) | 10 (100) | 15 (71.4) |

^a^By biopsy and noninvasive testing or best guess by investigator.
