# Supplementary material for: Impact of Safety-Related Dose Reductions or Discontinuations on Sustained Virologic Response in HCV-Infected Patients: Results from the GUARD-C Cohort
Source: PLoS One. 2016 Mar 28;11(3):e0151703. doi: 10.1371/journal.pone.0151703 (PMC4809570; doi:10.1371/journal.pone.0151703)
Supplement: S3 Table — (DOCX) [file pone.0151703.s007.docx]

**S3 Table. Baseline characteristics of 336 treatment-naive HCV mono-infected patients treated with peginterferon alfa-2b/ribavirin.**

|  | **G1** | **G2** | **G3** | **G4** | **Unknown** |
| --- | --- | --- | --- | --- | --- |
| **Characteristic** | **(n=126)** | **(n=68)** | **(n=85)** | **(n=55)** | **(n=2)** |
| **Male sex, n (%)** | 64 (50.8) | 27 (39.7) | 60 (70.6) | 48 (87.3) | 1 (50.0) |
| **Mean ± SD age, years** | 51.8 ± 12.1 | 55.5 ± 11.5 | 42.2 ± 10.5 | 41.3 ± 11.5 | 29.0 ± 0 |
| **Race, n (%)** |  |  |  |  |  |
| Caucasian/White | 117 (92.9) | 67 (98.5) | 72 (84.7) | 53 (96.4) | 2 (100) |
| Black | 2 (1.6) | – | – | 1 (1.8) | – |
| Asian/Oriental | 6 (4.8) | 1 (1.5) | 13 (15.3) | 1 (1.8) | – |
| Other | 1 (0.8) | – | – | – | – |
| **Mean ± SD body mass index, kg/m^2^** | 25.1 ± 3.7 | 25.05 ± 3.6 | 24.5 ± 3.8 | 26.2 ± 3.8 | 20.2 ± 1.3 |
| **Method to assess liver fibrosis, n (%)** |  |  |  |  |  |
| Biopsy | 50 (39.7) | 8 (11.8) | 26 (30.6) | 42 (76.4) | 1 (50.0) |
| Noninvasive method | 53 (42.1) | 32 (47.1) | 30 (35.3) | 4 (7.3) | 1 (50.0) |
| Best guess/Not assessed | 23 (18.3) | 28 (41.2) | 29 (34.1) | 9 (16.4) | – |
| **Result of liver fibrosis assessment, n (%)^a^** |  |  |  |  |  |
| Transition to cirrhosis/cirrhosis | 33/126 (26.2) | 9/68 (13.2) | 21/85 (24.7) | 7/55 (12.7) | – |
| No transition to cirrhosis/cirrhosis | 93/126 (73.8) | 59/68 (86.8) | 64/85 (75.3) | 48/55 (87.3) | 2/2 (100) |
| **Mean ± SD HCV RNA level, log_10_ IU/mL** | 5.79 ± 0.90 | 5.76 ± 0.95 | 5.68 ± 0.97 | 5.71 ± 0.71 | 5.26 ± 0.96 |
| **HCV RNA >800,000 IU/mL, n (%)** | 61/123 (49.6) | 35/67 (52.2) | 35/77 (45.5) | 24/55 (43.6) | 1/2 (50.0) |
| **Mean ± SD hemoglobin conc, g/L** | 153.5 ± 12.7 | 149.0 ± 14.5 | 152.5 ± 14.3 | 153.0 ± 14.8 | 162.8 ± 6.7 |
| **Mean ± SD neutrophil count x 10^9^/L** | 3.5 ± 1.3 | 3.5 ± 1.4 | 3.8 ± 1.7 | 3.3 ± 1.5 | 5.7 ± 3.0 |
| **Mean ± SD platelet count x 10^9^/L** | 205.2 ± 69.3 | 215.1 ± 80.2 | 207.5 ± 66.5 | 214.3 ± 55.0 | 275.5 ± 51.6 |
| **Mean ± SD ALT activity, IU/L** | 107.5 ± 84.8 | 84.2 ± 60.5 | 140.1 ± 115.7 | 88.1 ± 60.4 | 84.9 ± 8.3 |
| **Mean ± SD ALT ratio** | 2.0 ± 1.5 | 1.5 ± 1.1 | 2.6 ± 2.1 | 1.6 ± 1.1 | 1.5 ± 0.2 |
| **ALT ratio >3, n (%)** | 16/125 (12.8) | 6/66 (9.1) | 24/84 (28.6) | 3/54 (5.6) | – |
| **Assigned treatment duration, n (%)** |  |  |  |  |  |
| 24 weeks | 1 (0.8) | 67 (98.5) | 78 (91.8) | – | 1 (50.0) |
| 48 weeks | 125 (99.2) | 1 (1.5) | 7 (8.2) | 55 (100) | 1 (50.0) |

^a^By biopsy and noninvasive testing or best guess by investigator.
