# Supplementary material for: Impact of Safety-Related Dose Reductions or Discontinuations on Sustained Virologic Response in HCV-Infected Patients: Results from the GUARD-C Cohort
Source: PLoS One. 2016 Mar 28;11(3):e0151703. doi: 10.1371/journal.pone.0151703 (PMC4809570; doi:10.1371/journal.pone.0151703)
Supplement: S4 Table — (DOCX) [file pone.0151703.s008.docx]

**S4 Table Reasons for premature withdrawal: treatment-naive HCV mono-infected patients treated with peginterferon alfa/ribavirin.**

| **Reason for withdrawal, n (%)** | **All patients assigned to 24 or 48 weeks’ treatment with PegIFN alfa-2a or -2b plus RBV (N=3181)** | **Subgroup 1 Genotype 1 patients assigned to 48 weeks’ treatment with PegIFN alfa-2a/RBV (n=1497)** | **Subgroup 2 Noncirrhotic genotype 1 Caucasian patients assigned to 48 weeks’ treatment with PegIFN alfa-2a/RBV (n=951)** |
| --- | --- | --- | --- |
| **From PegIFN alfa** |  |  |  |
| Safety^a^ | 175 (5.5) | 96 (6.4) | 53 (5.6) |
| Insufficient therapeutic response | 301 (9.5) | 199 (13.3) | 99 (10.4) |
| Early/good response | 103 (3.2) | 32 (2.1) | 24 (2.5) |
| Other^b^ | 230 (7.2) | 103 (6.9) | 60 (6.3) |
| Total | 809 (25.4) | 430 (28.7) | 236 (24.8) |
| **From ribavirin** |  |  |  |
| Safety^a^ | 192 (6.0) | 104 (6.9) | 57 (6.0) |
| Insufficient therapeutic response | 300 (9.4) | 198 (13.2) | 99 (10.4) |
| Early/good response | 100 (3.1) | 30 (2.0) | 22 (2.3) |
| Other^b^ | 236 (7.4) | 105 (7.0) | 62 (6.5) |
| Total | 828 (26.0) | 437 (29.2) | 1. 5.2) |

^a^Adverse events / intercurrent illness.

^b^Includes failure to return; refused treatment, withdrew consent, or did not cooperate; administrative; and other.
