# Supplementary material for: Impact of Safety-Related Dose Reductions or Discontinuations on Sustained Virologic Response in HCV-Infected Patients: Results from the GUARD-C Cohort
Source: PLoS One. 2016 Mar 28;11(3):e0151703. doi: 10.1371/journal.pone.0151703 (PMC4809570; doi:10.1371/journal.pone.0151703)
Supplement: S5 Table — (DOCX) [file pone.0151703.s009.docx]

**S5 Table. Virologic response in 2845 treatment-naive HCV mono-infected patients treated with peginterferon alfa-2a/ribavirin.**

| **Genotype and response, n (%; 95% CI)** | **24 weeks PegIFN alfa-2a/RBV** | **48 weeks PegIFN alfa-2a/RBV** |
| --- | --- | --- |
| **Genotype 1** | n=11 | n=1497 |
| Week 4 VR | 4 (36.4; 10.9, 69.2) | 196 (13.1; 11.4, 14.9) |
| Week 12 VR | 8 (72.7; 39.0, 94.0) | 884 (59.1; 56.5, 61.6) |
| EOT VR | 10 (90.9; 58.7, 99.8) | 995 (66.5; 64.0, 68.9) |
| SVR24 | 9 (81.8; 48.2, 97.7) | 708 (47.3; 44.7, 49.9) |
| Relapse, n/N (%; 95% CI)^a^ | 1/10 (10.0; 0.3, 44.5) | 212/911 (23.3; 20.6, 26.2) |
| **Genotype 2** | n=288 | n=6 |
| Week 4 VR | 196 (68.1; 62.3, 73.4) | 5 (83.3; 35.9, 99.6) |
| Week 12 VR | 247 (85.8; 81.2, 89.6) | 5 (83.3; 35.9, 99.6 ) |
| EOT VR | 260 (90.3; 86.3, 93.4) | 5 (83.3; 35.9, 99.6) |
| SVR24 | 224 (77.8; 72.5, 82.4) | 3 (50.0; 11.8, 88.2) |
| Relapse, n/N (%; 95% CI)^a^ | 18/241 (7.5; 4.5, 11.5) | 1/4 (25.0; 0.6, 80.6) |
| **Genotype 3** | n=636 | n=35 |
| Week 4 VR | 334 (52.5; 48.6, 56.5) | 19 (54.3; 36.6, 71.2) |
| Week 12 VR | 432 (67.9; 64.1, 71.5) | 27 (77.1; 59.9, 89.6) |
| EOT VR | 541 (85.1; 82.1, 87.7) | 29 (82.9; 66.4, 93.4) |
| SVR24 | 442 (69.5; 65.8, 73.1) | 18 (51.4; 34.0, 68.6) |
| Relapse, n/N (%; 95% CI)^a^ | 51/486 (10.5; 7.9, 13.6) | 5/23 (21.7; 7.5, 43.7) |
| **Genotype 4** | n=3 | n=338 |
| Week 4 VR | 2 (66.7; 9.4, 99.2) | 119 (35.2; 30.1, 40.6) |
| Week 12 VR | 2 (66.7; 9.4, 99.2) | 250 (74.0; 68.9, 78.6) |
| EOT VR | 2 (66.7; 9.4, 99.2) | 236 (69.8; 64.6, 74.7) |
| SVR24 | 2 (66.7; 9.4, 99.2) | 178 (52.7; 47.2, 58.1) |
| Relapse, n/N (%; 95% CI)^a^ | 0/2 (0.0; 0.0, 84.2) | 27/203 (13.3; 9.0, 18.8) |

CI, confidence interval; SVR, sustained virologic response; VR, virologic response

Data from patients infected with G5, or 6 (n = 10) and unknown genotypes (n = 21) are not presented.

^a^Calculations of relapse rates are restricted to patients with an end-of-treatment virologic response who had an HCV RNA test result in the SVR24 time window or whose last follow-up HCV RNA test did not show VR. Data are presented as: patients with relapse during follow-up (i.e. no SVR24) / total number of patients with an end-of-treatment virologic response as described above.
