# Supplementary material for: Impact of Safety-Related Dose Reductions or Discontinuations on Sustained Virologic Response in HCV-Infected Patients: Results from the GUARD-C Cohort
Source: PLoS One. 2016 Mar 28;11(3):e0151703. doi: 10.1371/journal.pone.0151703 (PMC4809570; doi:10.1371/journal.pone.0151703)
Supplement: S6 Table — (DOCX) [file pone.0151703.s010.docx]

**S6 Table. Virologic response in 336 treatment-naive HCV mono-infected patients treated with peginterferon alfa-2b/ribavirin.**

| **Genotype and response, n (%; 95% CI)** | **24 weeks PegIFN alfa-2b/RBV** | **48 weeks PegIFN alfa-2b/RBV** |
| --- | --- | --- |
| **Genotype 1** | n=1 | n=125 |
| Week 4 VR | 1 (100; 2.5, 100) | 17 (13.6; 8.1, 20.9) |
| Week 12 VR | 1 (100; 2.5, 100) | 65 (52.0; 42.9, 61.0) |
| EOT VR | 1 (100; 2.5, 100) | 71 (56.8; 47.6, 65.6) |
| SVR24 | 1 (100; 2.5, 100) | 36 (28.8; 21.1, 37.6) |
| Relapse, n/N (%; 95% CI)^a^ | 0/1 (0.0; 0.0, 97.5) | 25/60 (41.7; 29.1, 55.1) |
| **Genotype 2** | n=67 | n=1 |
| Week 4 VR | 51 (76.1; 64.1, 85.7) | 0 |
| Week 12 VR | 63 (94.0; 85.4, 98.3) | 0 |
| EOT VR | 62 (92.5; 83.4, 97.5) | 1 (100; 2.5, 100) |
| SVR24 | 51 (76.1; 64.1, 85.7) | 1 (100; 2.5, 100) |
| Relapse, n/N (%; 95% CI)^a^ | 5/55 (9.1; 3.0, 20.0) | 0/1 (0.0; 0.0, 97.5) |
| **Genotype 3** | n=78 | n=7 |
| Week 4 VR | 50 (64.1; 52.4, 74.7) | 4 (57.1; 18.4, 90.1) |
| Week 12 VR | 66 (84.6; 74.7, 91.8) | 4 (57.1; 18.4, 90.1) |
| EOT VR | 68 (87.2; 77.7, 93.7) | 5 (71.4; 29.0, 96.3) |
| SVR24 | 51 (65.4; 53.8, 75.8) | 3 (42.9; 9.9, 81.6) |
| Relapse, n/N (%; 95% CI)^a^ | 10/61 (16.4; 8.2, 28.1) | 2/5 (40.0; 5.3, 85.3) |
| **Genotype 4** | n=0 | n=55 |
| Week 4 VR | – | 17 (30.9; 19.1, 44.8) |
| Week 12 VR | – | 28 (50.9; 37.1, 64.6) |
| EOT VR | – | 35 (63.6; 49.6, 76.2) |
| SVR24 | – | 23 (41.8; 28.7, 55.9) |
| Relapse, n/N (%; 95% CI)^a^ | – | 5/27 (18.5; 6.3, 38.1) |

CI, confidence interval; SVR, sustained virologic response; VR, virologic response

Data from patients infected with unknown genotypes (n=2) are not presented.

^a^Calculations of relapse rates are restricted to patients with an end-of-treatment virologic response who had an HCV RNA test result in the SVR24 time window or whose last follow-up HCV RNA test did not show VR. Data are presented as: patients with relapse during follow-up (i.e. no SVR24) / total number of patients with an end-of-treatment virologic response as described above.
