# Supplementary material for: Impact of Safety-Related Dose Reductions or Discontinuations on Sustained Virologic Response in HCV-Infected Patients: Results from the GUARD-C Cohort
Source: PLoS One. 2016 Mar 28;11(3):e0151703. doi: 10.1371/journal.pone.0151703 (PMC4809570; doi:10.1371/journal.pone.0151703)
Supplement: S7 Table — (DOCX) [file pone.0151703.s011.docx]

**S7 Table. Distribution of patients and SVR24 rates according to baseline score.**

| **Baseline score** | **Number of patients with score, n/N (%)** | **SVR n/N (%)^a^** |
| --- | --- | --- |
| **0** | 30/917 (3.3) | 6/30 (20) |
| **1** | 197/917 (21.5) | 91/197 (46.2) |
| **2** | 173/917 (18.9) | 82/173 (47.4) |
| **3** | 206/917 (22.5) | 107/206 (51.9) |
| **4** | 150/917 (16.3) | 85/150 (56.7) |
| **5** | 92/917 (10.0) | 64/92 (69.6) |
| **6** | 46/917 (5.0) | 35/46 (76.1) |
| **7** | 19/917 (2.1) | 15/19 (78.9) |
| **8** | 4/917 (0.4) | 4/4 (100) |

^a^P<0.0001
