# Supplementary material for: Impact of Safety-Related Dose Reductions or Discontinuations on Sustained Virologic Response in HCV-Infected Patients: Results from the GUARD-C Cohort
Source: PLoS One. 2016 Mar 28;11(3):e0151703. doi: 10.1371/journal.pone.0151703 (PMC4809570; doi:10.1371/journal.pone.0151703)
Supplement: S8 Table — (DOCX) [file pone.0151703.s012.docx]

**S8 Table. Reasons for first safety-related dose reduction or discontinuation (sr-RD): treatment-naive HCV mono-infected patients treated with peginterferon alfa/ribavirin.**

| **Reason for (sr-RD),^a^ n (%)** | **All patients assigned to 24 or 48 weeks’ treatment with PegIFN alfa-2a or -2b plus RBV (N=3181)** | **Subgroup 1 Genotype 1 patients assigned to 48 weeks’ treatment with PegIFN alfa-2a/RBV (n=1497)** | **Subgroup 2 Noncirrhotic genotype 1 Caucasian patients assigned to 48 weeks’ treatment with PegIFN alfa-2a/RBV (n=951)** |
| --- | --- | --- | --- |
| **Patients with an sr-RD of PegIFN alfa** | 538 (16.9) | 319 (21.3) | 175 (18.4) |
| Total no. of events (1^st^ sr-RD of PegIFN alfa) | 672 | 393 | 223 |
| Incidence of individual events,^b^ n (%) |  |  |  |
| Neutropenia | 218 (6.9) | 137 (9.2) | 77 (8.1) |
| Thrombocytopenia | 110 (3.5) | 79 (5.3) | 35 (3.7) |
| Anemia | 39 (1.2) | 17 (1.1) | 9 (0.9) |
| Asthenia | 32 (1.0) | 22 (1.5) | 14 (1.5) |
| **Patients with an sr-RD of ribavirin** | 694 (21.8) | 441 (29.5) | 271 (28.5) |
| Total no. of events (1^st^ sr-RD of ribavirin) | 822 | 511 | 314 |
| Incidence of individual events,^b^ n (%) |  |  |  |
| Anemia | 417 (13.1) | 282 (18.8) | 173 (18.2) |
| Weight decreased | 65 (2.0) | 47 (3.1) | 32 (3.4) |
| Asthenia | 28 (0.9) | 17 (1.1) | 1. 1.2) |

^a^Multiple occurrences of the same adverse event/laboratory abnormality in one individual counted only once. All laboratory abnormalities and adverse events documented as main or additional reason are counted, i.e. a patient may have had multiple reasons.

^b^Events that occurred in ≥1% of patients of at least one population or subpopulation are shown.
