# Supplementary material for: Impact of Safety-Related Dose Reductions or Discontinuations on Sustained Virologic Response in HCV-Infected Patients: Results from the GUARD-C Cohort
Source: PLoS One. 2016 Mar 28;11(3):e0151703. doi: 10.1371/journal.pone.0151703 (PMC4809570; doi:10.1371/journal.pone.0151703)
Supplement: S9 Table — (DOCX) [file pone.0151703.s013.docx]

**S9 Table. Safety: treatment-naive HCV mono-infected patients treated with peginterferon alfa/ribavirin.**

|  | **All patients assigned to 24 or 48 weeks’ treatment with PegIFN alfa-2a or -2b plus RBV (N=3181)** | **Subgroup 1 Genotype 1 patients assigned to 48 weeks’ treatment with PegIFN alfa-2a/RBV (n=1497)** | **Subgroup 2 Noncirrhotic genotype 1 Caucasian patients assigned to 48 weeks’ treatment with PegIFN alfa-2a/RBV (n=951)** |
| --- | --- | --- | --- |
| **Deaths, n (%)** | 11^a^ (0.3) | 3^b^ (0.2) | 2^c^ (0.2) |
| **Patients with AEs leading to discontinuation of PegIFN alfa** | 178 (5.6) | 97 (6.5) | 54 (5.7) |
| **Patients with ≥1 SAE, n (%)** | 189 (5.9) | 101 (6.7) | 57 (6.0) |
| **No. of SAEs** | 242 | 130 | 69 |
| **Patients with ≥1 AE, n (%)** | 2263 (71.1) | 1163 (77.7) | 720 (75.7) |
| **No. of AEs** | 9931 | 5578 | 3384 |
| **Incidence of individual AEs^d^** |  |  |  |
| Anemia | 799 (25.1) | 517 (34.5) | 302 (31.8) |
| Asthenia | 515 (16.2) | 316 (21.1) | 201 (21.1) |
| Weight decreased | 497 (15.6) | 304 (20.3) | 193 (20.3) |
| Neutropenia | 484 (15.2) | 320 (21.4) | 189 (19.9) |
| Thrombocytopenia | 405 (12.7) | 300 (20.0) | 174 (18.3) |
| Pyrexia | 392 (12.3) | 176 (11.8) | 106 (11.1) |
| Fatigue | 380 (11.9) | 188 (12.6) | 117 (12.3) |
| Headache | 327 (10.3) | 180 (12.0) | 105 (11.0) |
| Leukopenia | 294 (9.2) | 207 (13.8) | 1. 7.0) |

^a^Causes of death include: gastrointestinal hemorrhage, upper gastrointestinal hemorrhage, or esophageal hemorrhage (n=3); accident or road traffic accident (n=2); completed suicide (n=2); cardiac failure or myocardial infarction (n=2); hepatic failure (n=1); acute pancreatitis (n=1); portal vein thrombosis (n=1); septic shock (n=1). The number of causes of death (n=13) exceeds the number of deaths (n=11) because both primary and underlying causes of death are presented.

^b^Causes of death include: cardiac failure (n=1); myocardial infarction (n=1); esophageal hemorrhage (n=1).

^c^Causes of death include: cardiac failure (n=1); myocardial infarction (n=1).

^d^AEs that occurred in ≥10% of patients in at least 1 group.
